# Anaerobic prokaryotic processes drive manganese release in a drinking water reservoir

**DOI:** 10.3389/fmicb.2025.1671749

**Published:** 2025-10-15

**Authors:** Lea Hahn, Solveig Tabea Vriesen, Gabriele Packroff, Jutta Meier, Werner Manz

**Affiliations:** ^1^Department of Biology, Institute for Integrated Natural Sciences, University of Koblenz, Koblenz, Germany; ^2^Wahnbach Reservoir Association (Wahnbachtalsperrenverband – WTV), Siegburg, Germany

**Keywords:** manganese redox cycling, sediment biogeochemistry, 16S rRNA gene sequencing, prokaryotic metabolic processes, drinking water reservoir

## Abstract

**Introduction:**

Elevated manganese (Mn) concentrations in drinking water reservoirs present challenges for raw water treatment. During thermal stratification, a shift from oxic to anoxic conditions at the sediment-water interface intensifies the release of dissolved Mn into the water column.

**Methods:**

High-throughput amplicon sequencing of 16S rRNA genes was used to identify the prokaryotic community in sediments of the Wahnbach Reservoir, examining abundance, diversity, and potential metabolic processes concerning key physicochemical parameters. In addition, cultivation-based methods clarified the role of Mn cycling and related biogeochemical processes.

**Results:**

Sediment analyses revealed high sedimentary Mn contents and elevated Mn^2+^ concentrations in pore water. Bioinformatic analysis of 16S rRNA gene sequences revealed a diverse prokaryotic community involved in Mn cycling and competing redox processes, both in sediment samples and enrichment cultures selective for Mn-transforming organisms. Dominant metabolic processes included anaerobic respiration, such as methanogenesis and the reduction of Mn, Fe, sulfate, as well as nitrate, alongside oxidative processes like nitrification and methanotrophy. Cultivation-based approaches confirmed the relevance of these processes and uncovered interconnections among them through the enrichment of specific genera, including *Rhodoferax*, a typical Mn reducer, *Ellin6067*, an ammonium oxidizer, and the methanogen *Methanosarcina*.

**Discussion:**

Seasonal oxygen depletion promotes the release of Mn and Fe from sediments, with Mn(IV) and Fe(III) reduction occurring under increasingly reducing conditions and contributing to metal cycling and redox zonation. This study highlights the dynamic interaction between physicochemical gradients and prokaryotic community structure that drives Mn transformation in stratified freshwater systems.

## 1 Introduction

Manganese is an essential trace element crucial for a wide range of metabolic and enzymatic processes across all domains of life (Friedman et al., 2024; Li and Yang, 2018). As the second most abundant trace metal after iron, Mn naturally occurs in oxidation states +II, +III, and +IV (Sujith and Bharathi, 2011). It predominantly exists in solid form, commonly as oxides, carbonates, silicates, or sulfides (Bryant et al., 2012; Ehrlich and Newman, 2008; LaRowe et al., 2021; Okita and Shanks, 1992; Schaller and Wehrli, 1997). Among these, Mn(IV) (oxyhydr)oxides are particularly reactive, functioning as powerful natural oxidants. They play a key role in redox transformation and, due to their negatively charged surfaces, strongly influence the mobility and bioavailability of trace metals and contaminants by binding metal cations (Godwin et al., 2020; Lovley, 1991; Tebo et al., 2004). In anoxic environments, Mn(IV) (oxyhydr)oxides are reduced either abiotically or via microbial mediation, resulting in the release of soluble Mn^2+^ (Davison, 1993; Giles et al., 2016; LaRowe et al., 2021; Munger et al., 2019).

Microbial Mn(II) oxidation plays a central role in Mn cycling by accelerating oxidation rates by decreasing the activation energy (Carmichael and Bräuer, 2015; Diem and Stumm, 1984; Godwin et al., 2020; Gounot, 1994; Tebo et al., 2004). This process may occur directly via specific proteins or enzymes, such as multicopper oxidases (Corstjens et al., 1997; Gounot, 1994; Kurdi et al., 2022; Tebo et al., 2004; Watanabe et al., 2024), or indirectly through the action of microbially produced oxidants like hydrogen peroxide and free radicals (Carmichael and Bräuer, 2015; Gounot, 1994). Mn(II) can also serve as an electron donor in aerobic respiration, and in combination with CO_2_ fixation, it supports a chemolithoautotrophic lifestyle (Gotore et al., 2025; Yu and Leadbetter, 2020). Under anoxic conditions, Mn(IV) minerals are reduced. Given its high redox potential, Mn(IV) acts as a favorable terminal electron acceptor during anaerobic respiration (Davison, 1993). Microorganisms can couple Mn(IV) reduction to the oxidation of organic compounds, methane, ammonium, and sulfide (Godwin et al., 2020; Gounot, 1994; Lovley, 1991; Wang et al., 2022). Overall, microbially mediated Mn redox transformation not only provides energy for microbial metabolism but may also serve protective functions, including shielding against toxic metals, ultraviolet radiation, and reactive oxygen species (Horsburgh et al., 2002; Sujith and Bharathi, 2011).

Redox conditions in freshwater systems, such as lakes and reservoirs, vary considerably across the water column and sediments, with oxygen availability acting as a key driver (Davison, 1993; Lau et al., 2016; Schaller and Wehrli, 1997). During thermal stratification, the development of an anoxic hypolimnion promotes anaerobic microbial processes, leading to the accumulation of dissolved Mn^2+^ and Fe^2+^, along with the release of other nutrients (Davison, 1993; Lau et al., 2016; Lovley, 1991; Wang et al., 2022). Although Fe is typically more abundant, Mn can reach comparable concentrations under reducing conditions (Canfield et al., 2005). Despite its lower overall abundance, Mn(IV) plays a pivotal role in redox cycling, acting as a key electron acceptor alongside nitrate, Fe(III), and sulfate (Emerson, 2000; Gounot, 1994; Lovley, 1991; Wang et al., 2022; Zhang K. et al., 2022). Importantly, dissolved Mn^2+^ serves as a more sensitive redox indicator than Fe^2+^, as Mn(IV) oxides are reduced at higher redox potentials than their Fe(III) counterparts (Davison, 1993; Sikora et al., 2017).

Dissolved Mn is widespread in water supplies, including reservoirs and groundwater, and frequently exceeds guideline limits set by the World Health Organization (WHO) and the European Drinking Water Directive (European Parliament and Council of the European Union, 2020; Frisbie et al., 2012; WHO, 2021). Elevated Mn concentrations not only cause aesthetic issues, such as a brown discoloration, but also create technical challenges due to the oxidation of Mn^2+^ during water processing (Li et al., 2022; Tobiason et al., 2016; WHO, 2021; Zhou et al., 2020). In drinking water distribution systems (DWDS), Mn often accumulates as Mn(IV) oxide deposits, which can stimulate biofilm formation and comprise water quality (Li et al., 2022; Zhou et al., 2020). Effective Mn removal requires tailored physical, chemical, or biological treatment strategies adapted to the specific water chemistry and operational conditions of the treatment plant (Tobiason et al., 2016).

The Wahnbach Reservoir, a major drinking water source in North Rhine-Westphalia, Germany, is routinely monitored with high temporal and spatial resolution of the water column to ensure water quality (Herschel, 1995). Over recent decades, shifts in key physicochemical parameters have been documented, affecting both (micro)biological activity and overall biogeochemical cycling within the reservoir (Herschel, 1995; Willmitzer et al., 2015). Notable changes include rising surface water temperatures, earlier and prolonged periods of thermal stratification, less or delayed mixing events, and altered nutrient fluxes (Delpla et al., 2009; Firoozi et al., 2020; Leveque et al., 2021; Willmitzer et al., 2015). During summer stagnation, hypolimnetic measurements revealed declining O_2_ levels alongside elevated Mn^2+^ concentrations. Despite these observations, the role of sedimentary Mn reservoirs and their interactions with microbial communities remains poorly understood and has received limited scientific attention to date (Herschel, 1995).

This study explores Mn redox cycling in the Wahnbach Reservoir, emphasizing the sediment’s dual role as both a source of dissolved Mn^2+^ and a reservoir of Mn(IV) oxides that support Mn-transforming microbial communities at the oxic-anoxic sediment-water interface. Water and sediment core samples were collected from three sampling locations at different water depths and on three sampling dates, capturing a range of redox conditions. Depth profiles of Mn, Fe, and other key parameters were quantified in sediments and pore waters. The prokaryotic community was characterized via 16S rRNA gene amplicon sequencing from both native sediments and enrichment cultures targeting Mn-transforming microorganisms. Enrichments were established using dilution series in selective media, enabling quantification by the most probable number (MPN) method. Microbial metabolisms were inferred from sequence data, and to assess the relative abundance of Mn-transforming prokaryotes, a curated list of 161 relevant genera was compiled.

## 2 Materials and methods

### 2.1 Study site and sampling

**Figure 1:**
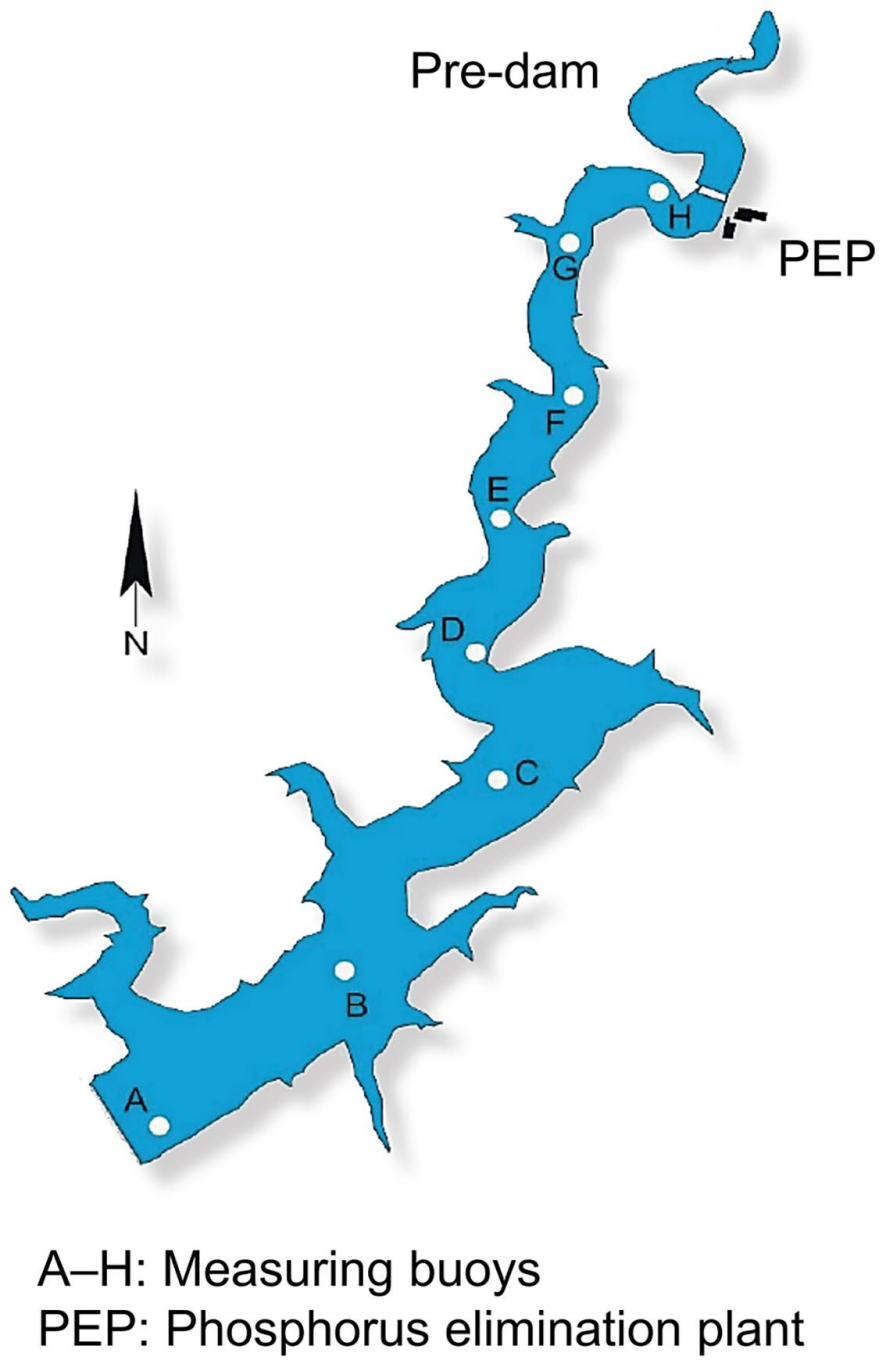
Schematic map with top view of the Wahnbach Reservoir. It shows the pre-dam, phosphorus elimination plant (PEP), and measuring buoys A–H (https://www.wahnbach.de).

The Wahnbach Reservoir, located near Siegburg in North Rhine-Westphalia, Germany,^1^ served as our study site. Managed by the Wahnbach Reservoir Association (Wahnbachtalsperrenverband – WTV), this reservoir supplies drinking water to over 800,000 residents, primarily drawing from the reservoir itself, with groundwater supplementation. Covering a catchment area of 71.5 km^2^, the area is dominated by over 50% agriculture, 22% forestry, and scattered small residential zones. The reservoir reaches a maximum depth of 46 m, stretches 5.8 km in length, and holds up to 41.3 · 10^6^ m^3^ of water, delivering 80,000 m^3^ of raw water daily for drinking water production. The primary inflow is the Wahnbach stream, with a phosphorus elimination plant (PEP) located between the pre-dam and the main reservoir. Classified as oligo- to slightly mesotrophic and monomictic, the Wahnbach Reservoir undergoes thermal stratification in summer and mixing beginning in autumn. Sampling was conducted at three sampling locations: buoy A (deepest point near the dam, max. 46 m), buoy E (mid-reservoir, max. 20 m), and buoy H (just behind the pre-dam and PEP, max. 9.5 m) (Figure 1). Raw water extraction occurs at buoy A approximately 2 m above the sediment surface. The WTV routinely monitors physical, chemical, and biological parameters using weekly multiparameter probe measurements, in conjunction with monthly laboratory analyses. The dataset presented here comprises water samples collected in 2022 on July 12, September 13, and October 11 at buoy A, and July 5, September 6, and October 5 at buoy E. Water samples were collected at buoy H only on July 5 as the sampling location could not be reached by boat in September and October due to low water levels. Measurements with a multiparameter probe were conducted the following day. In addition, 1-L water samples (two per buoy and sampling date) were collected in acid-rinsed bottles during regular sampling for subsequent DNA extraction.

Sediment cores were retrieved in July, September, and October 2022 using Corer USC 06000 or USC 09000 (Uwitec GmbH, Mondsee, AT). On July 14, four cores each were obtained at buoys E and H, and one at buoy A. On September 1, four cores were collected only at buoy A. On October 13, six cores were retrieved at buoy A, four at buoy E, and none at buoy H due to low water level. Immediately after retrieval, redox measurements were performed in one core. The remaining cores were promptly sectioned into 1-cm slices for the upper 4 cm and into 2-cm slices below 4 cm. Cores from buoy A and those from buoy E in October were sectioned to a depth of 16 cm, while all others were processed to 10 cm. Sediment slices were transferred into airtight containers under anoxic conditions using Thermo Scientific^™^ Oxoid AnaeroGen 2.5-L bags (Thermo Fisher Scientific Inc., Waltham, MA, US), cooled with ice packs, and transported to the laboratory. Subsampling was conducted within 24 h in an anaerobic chamber (Coy Laboratory Products Inc., Grass Lake, MI, US). Pore water was extracted only from buoy A cores (one in September, two in October) by centrifugation outside the anaerobic chamber (3,864 x *g*, 20 min, 20 °C). Supernatants were immediately returned to the anaerobic chamber and filtered through 0.2-μm membrane filter. Subsamples for DNA extraction were stored at −80 °C, and DNA was extracted from 100–200 mg aliquots within 4 months (July), 17 months (September), and 16 months (October) of sampling. Sediment samples intended for Mn and Fe extractions were stored at −20 °C and processed within one week.

### 2.2 Physicochemical analysis

In the water column, temperature (°C), pH, and dissolved O_2_ (mM, saturation in %) were measured *in situ* using a multiparameter probe CTD90 (Sea and Sun Technology GmbH, Trappenkamp, DE). Acid-soluble and dissolved Mn and Fe concentrations (μM) were quantified according to DIN EN ISO 11885:2009-09 by inductively coupled plasma optical emission spectroscopy (ICP-OES) using a Varian 720 ES (Varian Inc., Palo Alto, CA, US). Total organic carbon (TOC; μM) was measured following DIN EN 1484:2019-04 by infrared spectroscopy with a DIMATOC^®^ 2000 (Dimatec Analysentechnik GmbH, Essen, DE). Particulate organic carbon (POC; μM) was determined from 1-L water samples filtered through pre-combusted glass fiber filters (GF/C; 480 °C, 4 h), compressed into pellets, and analyzed via combustion at 1,000 °C using a TruSpec^®^ Micro Analyzer (Leco Corporation, St. Joseph, MI, US). Dissolved organic carbon (DOC; μM) was calculated as the difference between TOC and POC. Sulfate (SO_4_^2−^; μM) was determined according to DIN EN ISO 11885:2009-09, measured by ICP-OES with a Varian 720 ES (Varian Inc., Palo Alto, CA, US). Nitrite (NO_2_^−^; μM) and nitrate (NO_3_^−^; μM) were measured following DIN EN ISO 13395:1996-12, while ammonium (NH_4_^+^; μM) was analyzed according to DIN EN ISO 11732:2005-05, and soluble inorganic phosphate (PO_4_^3−^; μM) was determined according to DIN EN ISO 15681-2:2019-05, each quantified by continuous flow analysis (CFA) using a SAN++^®^ Series CFA (Skalar Analytical B. V., Breda, NL).

In sediment samples, the redox potential (Eh; mV) was measured in 1-cm increments using a probe designed and applied according to Geist and Auerswald (2007). To determine the following sediment geochemistry, two cores each from buoys E and H and one core from buoy A were used in July, one core from buoy A in September, and three cores each from buoys A and E in October. Sediment mass density (g mL^−1^) was determined by weighing 2 mL of fresh sediment (fresh weight, FW; g). Dry weight (DW; g) and corresponding water content (%) as loss-on-drying (LOD; %; 105 °C, 24 h) were measured. Ash-free dry weight (AFDW; g) was obtained as loss-on-ignition (LOI; %; 519 °C, 5 h) and used to estimate organic matter content. Mn and Fe in sediment samples were extracted using two methods: (i) acid ammonium oxalate (using a solution of 113 mM (NH_4_)_2_C_2_O_4_ · H_2_O and 87 mM C_2_H_2_O_4_ · 2H_2_O, pH 3.25) and (ii) dithionite-citrate bicarbonate (using a buffer of 239 mM C_6_H_5_Na_3_O_7_ · 2H_2_O and 200 mM NaHCO_3_, ~1 g Na_2_S_2_O_4_ per sample, and a solution of 50 mM MgSO_4_ · 7H_2_O) (Canfield et al., 1993; Piper et al., 1984). Extracts were diluted in 2% (v/v) HNO_3_ and analyzed for Mn and Fe concentrations (mM) by atomic absorption spectrometry (AAS) using an AAnalyst 400 AA Spectrometer equipped with a Lumina™ hollow cathode lamp (Series N30502XX) (PerkinElmer Inc., Hopkinton, MA, US). Quantification was performed against standards for Mn (Supelco^®^ 1.19789) and Fe (Supelco^®^ 1.19781) obtained from Sigma-Aldrich Chemie GmbH (Taufkirchen, DE). Results were reported in g kg^−1^_DW_ or mM (mmol L^−1^ fresh sediment).

In sediment pore water samples, pH was measured immediately after extraction using an inoLab pH 720 pH meter (WTW, Xylem Analytics Germany Sales GmbH & Co. KG, Weilheim, DE). For the determination of dissolved Mn^2+^ and Fe^2+^ (μM), aliquots were diluted in 2% (v/v) HNO_3_ and stored at 4 °C until analysis by AAS. Pore water samples collected in October for the analysis of NO_2_^−^ (μM), NO_3_^−^ (μM), NH_4_^+^ (μM), and PO_4_^3−^ (μM) were stored at −20 °C. Concentrations were measured by a CFA system from Bran+Luebbe GmbH (Norderstedt, DE), comprising multiple AutoAnalyzer 3, XY-3 Sampler, and AutoAnalyzer 3 Digital Colorimeter. Samples for the analysis of SO_4_^2−^ (μM) were also stored at −20 °C and analyzed via ion chromatography (IC) using a 690 IC equipped with a 69 IC pump from Metrohm Deutschland GmbH & Co. KG (Filderstadt, DE).

### 2.3 Quantification of culturable Mn-transforming microorganisms

To quantify culturable Mn-transforming microorganisms, the most probable number (MPN) technique was applied with a triplicate dilution series of fresh sediment in selective media designed for three functional groups: (i) lithotrophic Mn oxidizers (litMnOx), (ii) organotrophic Mn oxidizers (orgMnOx), and (iii) Mn reducers (MnRed). Mineral medium containing 6 mM KCl, 4 mM NaCl, 1 mM CaCl · 2H_2_O, 1.9 mM MgCl_2_ · 6H_2_O, and 0.1 mM MgSO_4_ · 7H_2_O was based on the freshwater medium for sulfate-reducing bacteria (Widdel and Bak, 1992). Vitamin mixture containing thiamine and vitamin B_12_, non-chelated trace element solution, and selenite-tungstate solution were adapted from Widdel and Bak (1992). A 10 mM 4-(2-hydroxyethyl)-1-piperazineethanesulfonic acid (HEPES) buffer (pH 7.4) was applied. Selective Mn oxidizer media (based on Boogerd and de Vrind, 1987; Okazaki et al., 1997; Stein et al., 2001; Yu and Leadbetter, 2020) contained 2 mM KNO_3_ and 0.3 mM KH_2_PO_4_ as N and P sources, respectively, with freshly prepared Mn carbonate as Mn(II) source (~80 mM for litMnOx and ~20 mM for orgMnOx). Medium for organotrophic Mn oxidizers was additionally enriched with 0.5 g L^−1^ yeast extract, 0.5 g L^−1^ casamino acids, and 5 mM D(+)-glucose · H_2_O. Mn reducer medium (adapted from Lovley, 2006; Lovley and Phillips, 1988; Vandieken et al., 2012) contained additionally 5 mM NH_4_Cl, 1.5 mM KH_2_PO_4_, 5 mM Na-acetate, 5 mM Na-lactate, and ~14 mM freshly prepared Mn oxide as Mn(IV) source.

MPN dilution series were conducted for selected sediments from three depth intervals (0–2 cm, 2–4 cm, 8–10 cm) collected from the three sampling locations in July and October (Supplementary Table S3). The sediment samples were homogenized over the corresponding depths and, where available, pooled from multiple cores to ensure representative sampling. Initial dilutions were prepared in 100-mL sterile serum bottles containing 45 mL of selective medium and 5 mL of sediment (10% v/v). Serial dilutions were subsequently performed in 20-mL serum bottles (10-fold in July: 1 mL sediment slurry + 9 mL medium; 5.5-fold in October: 1 mL sediment slurry + 4.5 mL medium). After inoculation, Mn oxidizer bottles were sealed with sterile parafilm, while Mn reducer bottles were sealed with sterile butyl rubber stoppers. The headspace of the Mn reducer bottles was sparged with N_2_/CO_2_ (80:20 v/v) gas mixture to create anoxic conditions. All cultures were incubated at 20 °C. Mn oxidation is indicated by brown coloring, and Mn reduction by discoloration (Krumbein and Altmann, 1973; Wendt-Potthoff et al., 2014). MPN evaluation was carried out six weeks after inoculation using the EPA MPN Calculator (https://mostprobablenumbercalculator.epa.gov/mpnForm, version 2.0, U. S. Environmental Protection Agency, Washington, DC, 2013).

### 2.4 DNA extraction

For DNA extraction from MPN cultures, aliquots of 9 mL (July) and 4.5 mL (October) were taken from selected dilutions (Supplementary Table S3) after 15 weeks and 9 weeks of incubation, respectively. Microbial cells were harvested from culture liquid by centrifugation (4,153 x *g*, 10 °C, 20 min) and stored at −80 °C until further processing. DNA extractions were carried out 1 month (July) and 14 months (October) after sampling. Genomic DNA from both MPN enrichment cultures and sediment samples was extracted using the DNeasy PowerSoil Kit from Qiagen GmbH (Hilden, DE). For water samples, 1-L aliquots were filtered on the day of sampling using 0.2-μm cellulose acetate filters, which were immediately frozen at −80 °C and later processed using the DNeasy PowerWater Kit (Qiagen GmbH, Hilden, DE). DNA concentrations (nM) were measured using the Qubit dsDNA BR Assay Kit in combination with a Qubit 4 Fluorometer (Invitrogen by Thermo Fisher Scientific, Waltham, MA, US). All extracted DNA was stored at −80 °C for subsequent molecular analyses.

### 2.5 Sequencing and bioinformatic analysis

Bacterial community composition was assessed by targeting the V3-V4 region of the 16S rRNA gene. Amplicon Sequencing was conducted by Novogene Europe (Cambridge, UK, https://www.novogene.com/eu-en/) using the primers 341F (5′-CCTAYGGGRBGCASCAG-3′) and 806R (5′-GGACTACNNGGGTATCTAAT-3′). Libraries were sequenced on an Illumina NovaSeq 6000 platform (San Diego, CA, US) to generate 250 bp paired-end raw reads. Sequence analysis was performed by Novogene using the bioinformatics pipeline QIIME (Quantitative Insights Into Microbial Ecology). For the July samples, raw sequences were analyzed using QIIME1 (http://qiime.org/index-qiime1.html, version 1.9.1), with sequences clustered into operative taxonomic units (OTUs) at ≥97% similarity. Further analysis of phylogenetic relationships and dominant taxa was conducted using Uparse software (http://drive5.com/uparse/, Uparse v7.0.1001, Edgar, 2013), and multiple sequence alignment was performed using MUSCLE software (http://www.drive5.com/muscle/, version 3.8.31, Edgar, 2004). Taxonomic annotation was based on the SILVA Database (http://www.arbsilva.de/, SILVA138, Quast et al., 2013) using the Mothur algorithm. For the September and October samples, sequence processing was performed using QIIME2 (https://qiime2.org/, version QIIME2-202202), employing DADA2 (Callahan et al., 2016) for denoising and generating amplicon sequence variants (ASVs). Unlike OTU-based approaches, ASVs are resolved at 100% sequence similarity, using QIIME2’s classify-sklearn algorithm (Bokulich et al., 2018; Bolyen et al., 2019), a pre-trained Naive Bayes classifier, and the SILVA Database (SILVA138).

Major microbial metabolisms were identified using FAPROTAX (Functional Annotation of PROkaryotic TAXa; https://pages.uoregon.edu/slouca/LoucaLab/archive/FAPROTAX/lib/php/index.php, version 1.2.10, Louca et al., 2016). This tool assigns functional and metabolic traits to taxa based on curated taxonomic classifications linked to experimentally characterized strains and their known physiology from the literature. Functional assignments were generated using the collapse_table.py script, taking OTU or ASV tables as input. The script was executed using Python (https://www.python.org/psf-landing/, version 3.11.2, Python Software Foundation, Wilmington, DE, US) managed through miniconda3. Our analysis focused on key aerobic and lithotrophic processes (methanotrophy, nitrification, oxidation of sulfur compounds, Fe oxidation, and Mn oxidation) as well as on anaerobic respiration pathways (methanogenesis and respiration of nitrate, sulfate, Fe, and Mn). In the July sequencing dataset, 15.0% of all sequences were assigned to at least one functional or metabolic annotation of all processes compiled in FAPROTAX, and in the October sequence batch, 22.1% of the sequences were assigned. To enhance the resolution of Mn-transforming groups, we curated a comprehensive list of 161 prokaryotic genera associated with Mn cycling, compiled from an extensive literature research covering 235 articles (Supplementary Table S8).

Point and bar charts were created using Microsoft Excel LTSC MSO (16.0.14332.20824, Microsoft Corporation, Redmond, WA, US). Bubble plots, heatmaps, and, for advanced multivariate analyses, non-metric multidimensional scaling (nMDS) plots were generated with R (version 4.4.22024-10-31 ucrt, R Foundation for Statistical Computing, Vienna, AT) within R Studio (2024.12.0 Build 467, Posit Software, PBC, Boston, MA, US), employing the widely used “vegan” package (Oksanen et al., 2025) for ecological ordination and “ggplot2” (Wickham, 2016) for data visualization. To illustrate the core microbiota patterns in sediments, MPN cultures, and across the three sampling locations (buoys A, E, and H), ellipses representing 95% confidence intervals were drawn using a multivariate t-distribution (type = “t,” level = 0.95), providing a robust visualization of community clustering and variability.

## 3 Results

### 3.1 Physicochemical gradients in the water column

Surface temperatures peaked at 22.9–23.5 °C in July and September, dropping to 15.4–16.5 °C in October (Supplementary Figure S1). Thermal stratification developed at buoys A and E, with epilimnion depths of 6–7 m in July and September, respectively. Bottom water temperatures ranged from 6.4–7.0 °C (buoy A) to 8.1–12.0 °C (buoy E). O_2_ oversaturation occurred near the thermocline with up to 472 μM O_2_ (buoy A), but O_2_ levels declined sharply toward the sediment, reaching 69 μM (buoy A, October) and 3 μM (buoy E, September). The corresponding pH values ranged from alkaline in surface waters (8.0–8.9) to more acidic near the bottom (6.6–6.8). Remarkably, acid-soluble Mn was elevated in bottom waters, up to 9.4 μM at buoy A and 24.1 μM at buoy E, with dissolved Mn^2+^ contributing up to 83% (buoy A). Fe enrichment was observed only at buoy E in September/October (up to 5.9 μM). Mn consistently exceeded Fe by factors of up to 47 (buoy A) and 8 (buoy E). Buoy H showed no stratification or Mn and Fe enrichment.

POC remained lower than DOC at all sampling locations and sampling dates. POC ranged from 5.2–36.8 μM (buoy A), 24.0–71.0 μM (buoy E), and 42.7–49.6 μM (buoy H). DOC generally decreased with depth, but occasionally increased near sediments. Surface DOC was 147.9–212.4 μM, dropping to 101.7–143.2 μM (buoy A) and 110.2–166.0 μM (buoy E). Near the surface, SO_4_^2−^ was highest with 242.6–253.0 μM, with one notable peak at buoy H (803.7 μM, July). Values for PO_4_^3−^ remained very low (≤0.06 μM). Surface NO_2_^−^ peaked at 0.9–1.3 μM, decreased with depth, but increased again near sediments at buoys A and E. In surface layers, NO_3_^−^ ranged from 129.0–169.3 μM, peaking at 208.0 μM (buoy A) before declining near the sediment. Concentrations of NH_4_^+^ remained low throughout (0.3–3.4 μM), but rose near the sediment to 12.2 μM (buoy A) and 7.2 μM (buoy E). DNA concentrations were used as a proxy to indicate (microbial) biomass and showed spatial variation.

### 3.2 Geochemical characteristics in the sediment and pore water

**Figure 2:**
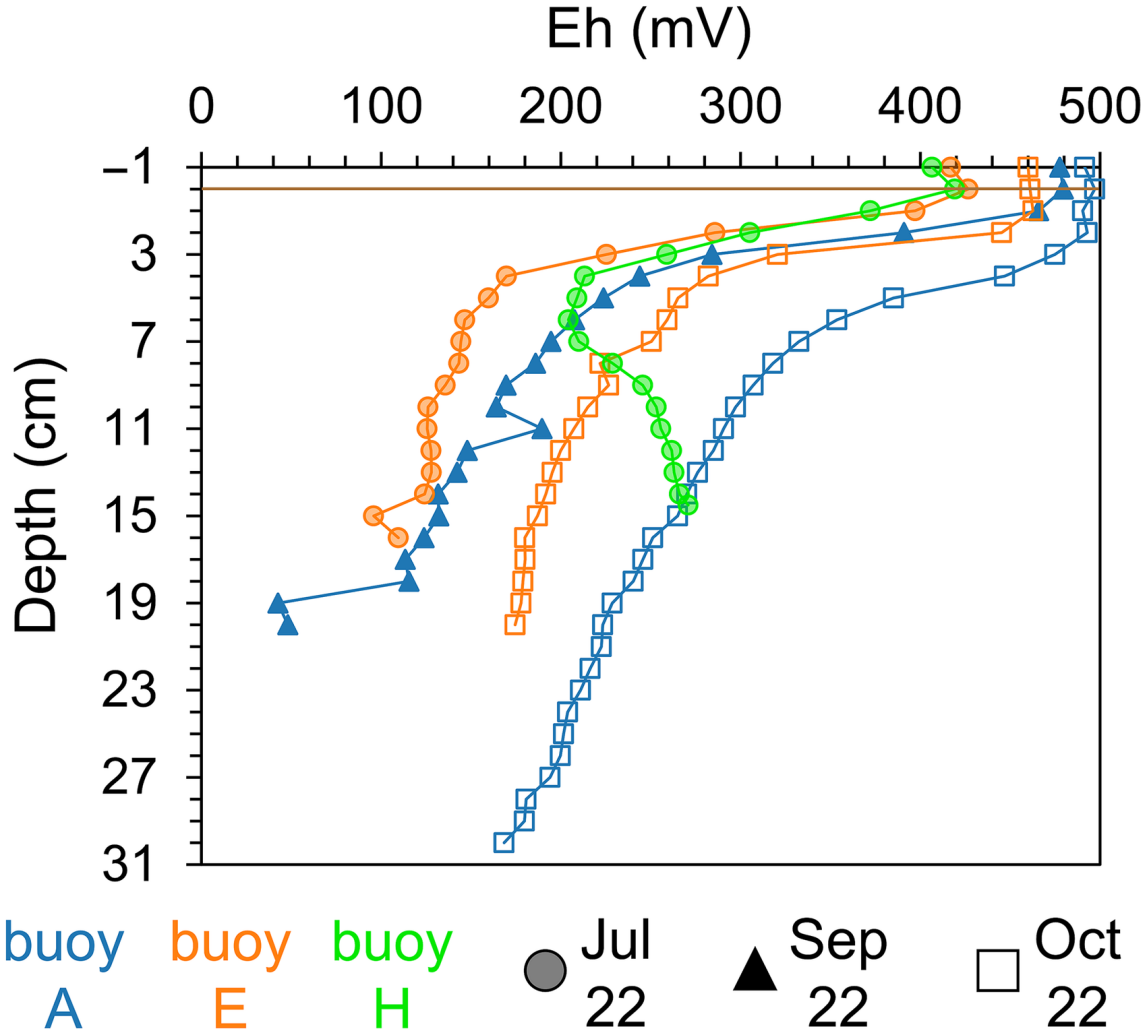
Redox potential (Eh, mV) of sediment layers (cm). Comparison across three sampling locations, partwise for samplings in July (shaded circle), September (filled triangle), and October (empty square): buoy A (blue), buoy E (orange), and buoy H (green). Horizontal brown line indicates the sediment-water interface.

Sediment color and texture, as determined by visual inspection, varied both spatially and vertically across sampling locations. At buoy A, surface sediments were brown to rust-colored and fluffy, transitioning to dark gray or black, loamy sediments with depth. Sediments at buoy E had a firmer brown surface, becoming dry, compact, and black deeper down. At buoy H, sediments were sandy, brownish with plant debris and increasingly darker zones indicative of organic matter accumulation. Redox potential profiles (Figure 2) showed strong stratification. Oxic conditions (>300 mV) were present in the overlying water and upper 2–3 cm of sediment across all sampling locations. Below this, redox potential declined sharply within 1–5 cm, marking the transition to anoxic conditions (<300 mV). At buoys A and E, redox values in sediment continued decreasing with depth. In contrast, redox potential in sediments at buoy H showed a minimum followed by a rise at depth. A seasonal redox shift toward more oxidizing conditions at depth was observed from summer to autumn, particularly in sediments at buoy E (July to October) and buoy A (September to October).

Water content across sediment profiles showed clear spatial and temporal variation (Supplementary Figure S2A). In sediments at buoy A, water content was high (67–90%) but dropped locally to 53–58% at 2–4 cm depth in July. That same month, surface sediments at buoys E and H had similarly high water content but declined more steeply with depth, reaching 37–53% in deeper layers. In October, water content ranged from 61 to 87% in sediments at buoys A and E, with consistently lower values at depth at buoy E. Organic matter content, assessed as AFDW, showed distinct depth trends (Supplementary Figure S2B). At buoy A in July, surface organic content in sediments peaked at 15%, declined to 3.5% at 2–4 cm, and then rose again to 7–9% in deeper layers. Sediments at buoys E and H showed a more gradual decrease of organic matter with depth (5–9%), with sediments at buoy H maintaining consistently higher values. In September, organic matter in sediments at buoy A reached 11% at 2–3 cm, while deeper layers stayed similar to July. By October, organic content in sediments at buoys A and E ranged from 5 to 8% throughout the profile.

**Figure 3:**
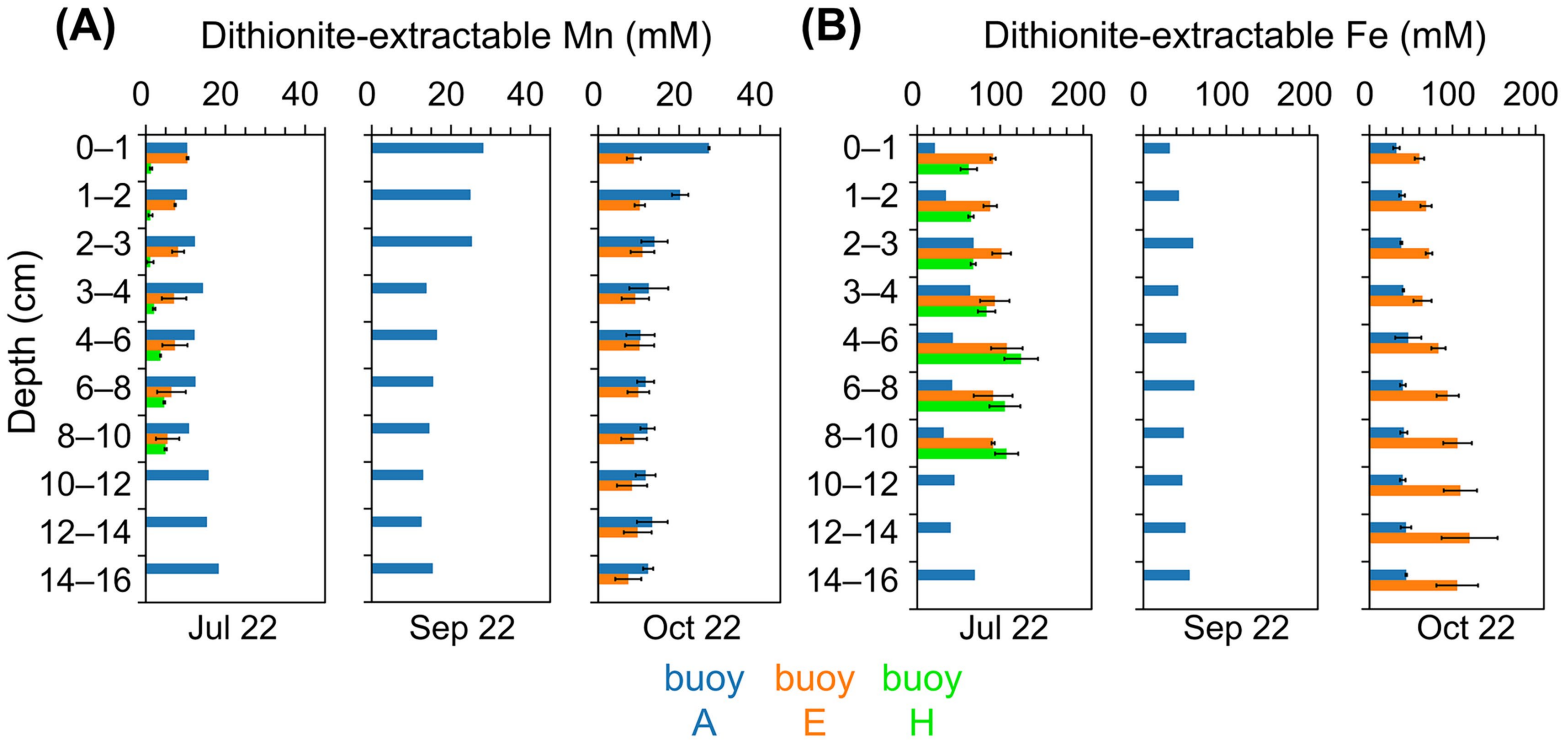
Dithionite-extractable Mn and Fe contents (mM) of sediment layers (cm). Comparison across three sampling locations, partwise for samplings in July, September, and October: buoy A (blue), buoy E (orange), and buoy H (green). **(A)** Mn and **(B)** Fe concentrations (mM).

Dithionite-extractable Mn reached the highest concentrations in sediments at buoy A (10–28 mM), far exceeding levels at other sampling locations (Figure 3A). In July, Mn increased slightly with depth, while in September and October, peak values were confined to the upper 2–3 cm. Sediments at buoy E showed moderate Mn concentrations (5–11 mM) with notable core-to-core variability in deeper layers. At buoy H, sediments exhibited consistently the lowest Mn levels (1–5 mM), with higher concentrations at greater depths. Dithionite-extractable Mn was generally comparable to oxalate-extractable Mn, except at depth, where oxalate-extractable Mn prevailed (Supplementary Figure S3A). Fe concentrations exceeded Mn at all sampling locations and depths, except in the surface sediment layers at buoy A (Figures 3B and Supplementary S3B). Dithionite-extractable Fe in sediments at buoy A ranged from 21 to 70 mM, the lowest among the sampling locations, while sediments at buoys E and H showed higher values (60–121 mM and 62–125 mM, respectively), typically increasing with depth. At buoy A, Fe concentrations in sediments were up to 5.5-fold higher than Mn; in sediments at buoys E and H, Fe exceeded Mn by factors of 6–25 and 18–59, particularly in deeper layers. Oxalate-extractable Fe was slightly higher than dithionite-extractable Fe, especially at depth. A complementary representation of Mn and Fe depth profiles in g kg^−1^_DW_ is provided in Supplementary Figure S4.

**Figure 4:**
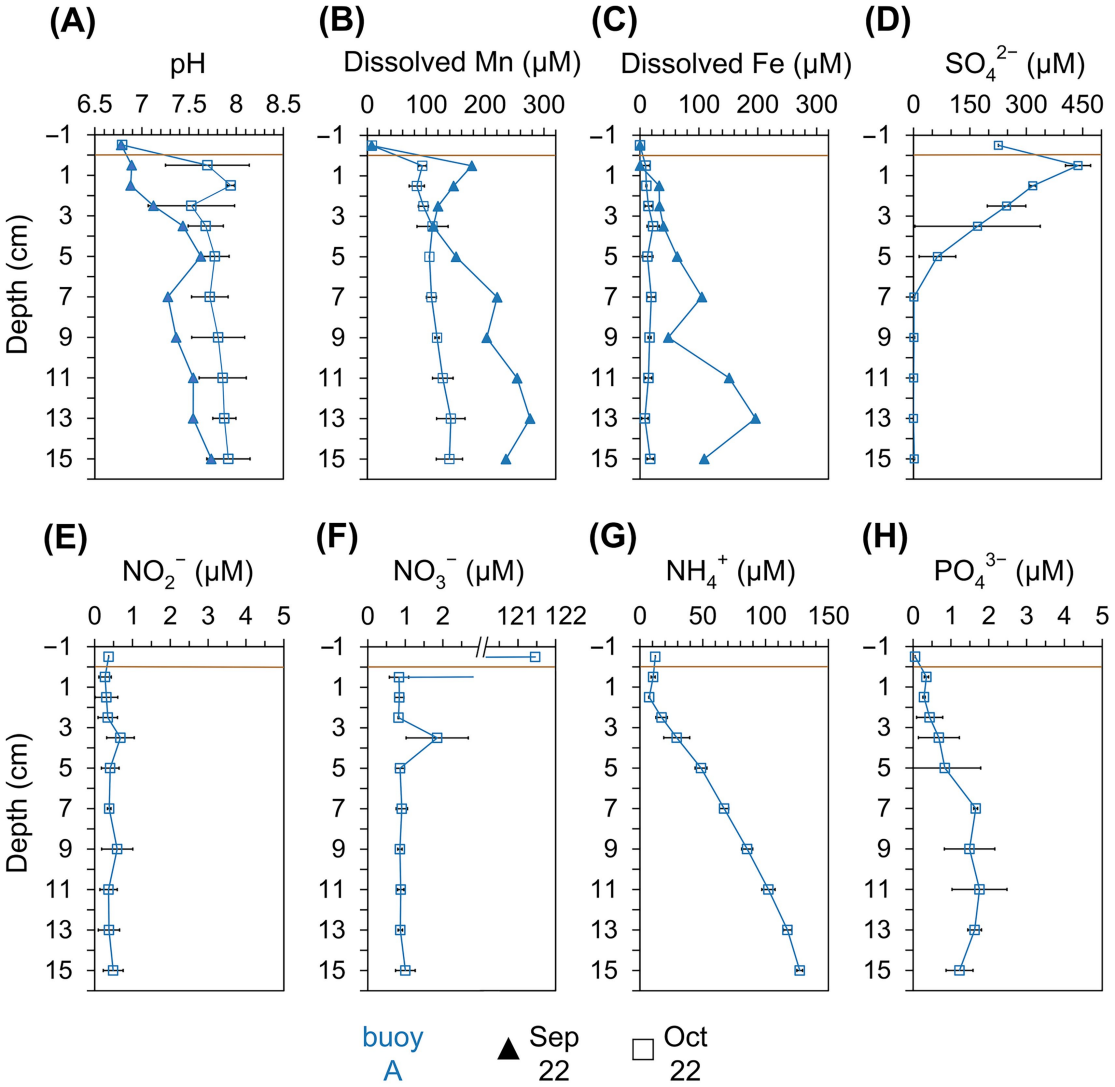
Physicochemical parameters in the pore water retrieved from different sediment layers (cm). Comparison across the sampling location of buoy A (blue) for samplings in September (filled triangle) and October (empty square). **(A)** pH, **(B)** dissolved Mn (μM), **(C)** dissolved Fe (μM), **(D)** SO_4_^2−^ (μM), **(E)** NO_2_^−^ (μM), **(F)** NO_3_^−^ (μM), **(G)** NH_4_^+^ (μM), and **(H)** PO_4_^3−^ (μM). Horizontal brown line indicates the sediment-water interface.

Pore water analysis at buoy A (Figure 4) from September and October showed pH values of 6.9–7.9, slightly increasing with depth. Dissolved Mn^2+^ and Fe^2+^ concentrations increased with depth, peaking at 276 μM (Mn) and 196 μM (Fe) in September. Mn^2+^ consistently exceeded Fe^2+^, indicating Mn as the dominant redox-active metal. In October, NO_2_^−^ and NO_3_^−^ remained low (~0.4 μM and ~0.8 μM), while NH_4_^+^ and PO_4_^3−^ increased with depth, reaching up to 127 μM and ~1.7 μM, respectively. In contrast, SO_4_^2−^ sharply declined from 438 μM in the upper 5 cm.

### 3.3 Prokaryotic community composition and metabolisms in the sediment

The ten most abundant prokaryotic classes across sediment samples were Gammaproteobacteria, Alphaproteobacteria, Vicinamibacteria, Bacteroidia, Bacilli, Clostridia, Methanosarcinia, Thermoleophilia, KD4-96, and Verrucomicrobiae, comprising 42.3–72.4% of total sequences (Supplementary Figure S5). Their distribution varied by sampling location and sampling date. Clear depth profiles were evident, with Gammaproteobacteria more abundant in surface sediments, while Bacilli, Clostridia, and Methanosarcinia prevailed in deeper layers, reflecting a shift toward anaerobic taxa. Gammaproteobacteria, the most abundant class, is metabolically diverse (Liu Q. et al., 2022; Liu W. et al., 2018; Newton et al., 2011; Song et al., 2024; Wu et al., 2017; Zemskaya et al., 2021). Bacilli and Clostridia form endospores and persist under unfavorable conditions; Clostridia contribute to anaerobic degradation of organic polymers and fermenting degradation products (Dürre, 2015; Huang et al., 2017; Song et al., 2024; Wu et al., 2017). Notably, Methanosarcinia were relatively abundant in the Wahnbach Reservoir as a class of methane-producing Archaea (Ji et al., 2016; Zemskaya et al., 2021). Alphaproteobacteria, Vicinamibacteria, Bacteroidia, Thermoleophilia, KD4-96, and Verrucomicrobiae showed no clear depth-related distribution patterns.

**Figure 5:**
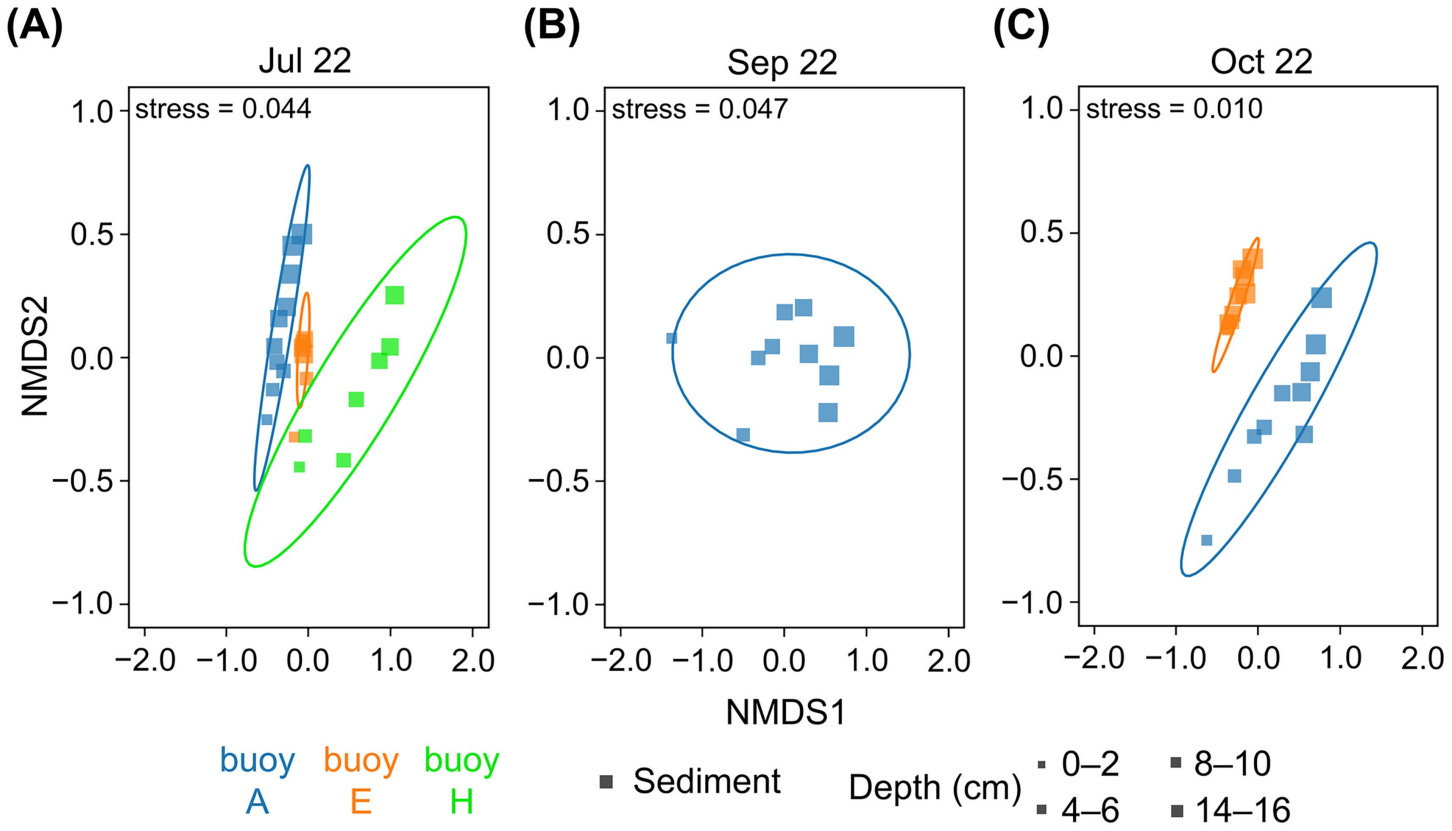
The nMDS plots of OTU/ASV distribution of sediment core samples based on 16S rRNA gene sequences. Comparison across the three sampling locations of buoy A (blue), buoy E (orange), and buoy H (green). Symbol size represents sediment depth (cm). Sediment samples (square) from **(A)** July, **(B)** September, and **(C)** October. Core microbiota indicated by ellipses based on multivariate t-distribution with a 95% confidence interval. Stress values are provided.

The nMDS plots (Figure 5) revealed distinct compositional differences and a clear clustering of prokaryotic communities by sampling location, as well as a gradient related to sediment depth. Communities in sediments at buoys A and E were more similar to each other. Those at buoy H, particularly in deeper sediments, were clearly divergent. The sediment depth-related gradient, most notably at buoy A (July and October) and buoy H (July), reflected the vertical stratification of microbial assemblages. The greatest similarities between the different sampling locations were found in the upper sediment layers; accordingly, deeper sediment layers increasingly differed.

**Figure 6:**
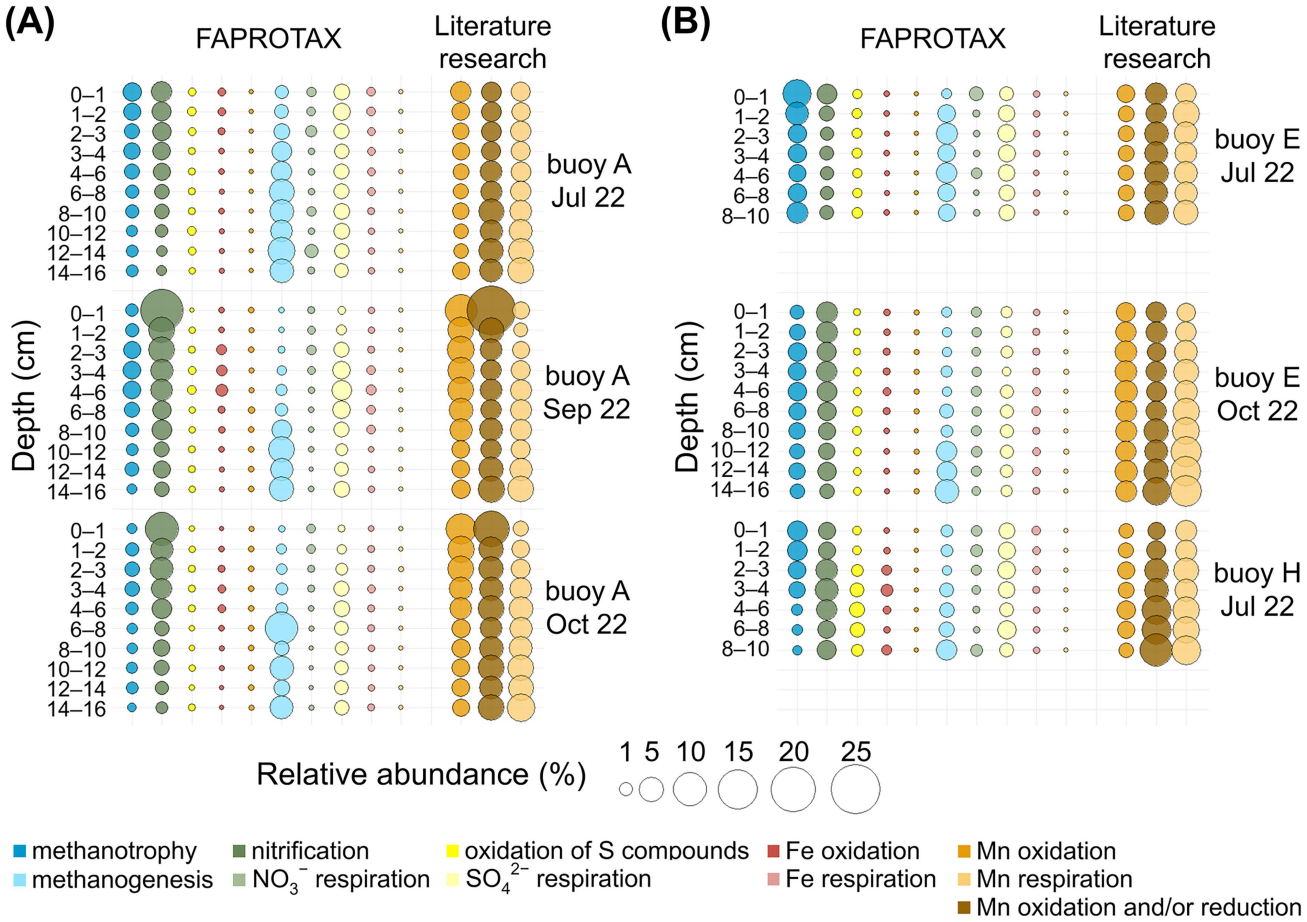
Bubble plots for relative abundance (%) of 16S rRNA gene sequences for selected metabolic pathways in sediment layers (cm). Comparison across three sampling locations. Functional assignments are based on FAPROTAX and own literature research. **(A)** For the sampling at buoy A in July, September, and October. **(B)** For the sampling at buoy E in July and October, and at buoy H in July.

The depth-resolved distribution of the key oxidizing and reducing metabolisms of interest, predicted by FAPROTAX and supplemented by our curated list of Mn-transforming genera (based on an extensive literature research), is presented in Figure 6, with dominant genera listed in Supplementary Tables S1, S2. Metabolic processes and associated prokaryotes are presented according to their relative abundance in FAPROTAX, starting with oxidizing processes, and concluding with a detailed description of the Mn-transforming organisms (Figure 6, Supplementary Tables S1, S2).

Methanotrophy (0.2–7.1%) was consistently present in sediments at all sampling locations, with the highest levels observed in surface sediments (0–2 cm) and declining with increasing depth. Key methanotrophs included “*Candidatus* Methylomirabilis”, *Crenothrix*, *Methylobacter*, and *Methylocystis*. The latter is capable of anoxic methane oxidation via nitrate reduction (Cheng et al., 2017; Su et al., 2022; Vigliotta et al., 2007). Nitrification was the most abundant process (up to 18.1%), highest in surface layers at buoy A, and decreased with depth. Dominant nitrifiers included *Ellin6067*, GOUTA6, and MND1. Next to *Ellin6067*, the typical nitrifier *Nitrospira* was detected, also noted as a potential Mn oxidizer (Laanbroek and Bollmann, 2011; Miyata et al., 2024; Palomo et al., 2016; Wang et al., 2014). Sulfur compound oxidation (up to 1.4%) was mainly detected in deeper sediments at buoy H, dominated by *Thiobacillus*. Fe oxidation was sporadic and low (≤0.6%), with *Gallionella* as the main genus. FAPROTAX-predicted Mn oxidation was rare (≤0.04%) and mostly absent; only *Pedomicrobium* was consistently assigned.

Literature research revealed higher Mn-oxidizer abundances (1.2–9.5%), decreasing with sediment depth at buoy A but stable at buoys E and H. Prominent genera included *Nitrospira*, *Crenothrix*, *Arenimonas*, and *Hyphomicrobium*, indicating greater diversity than captured by standard functional annotation tools. Most of these species are known to oxidize other reduced inorganic compounds: *Nitrospira* oxidizing NH_4_^+^, NO_2_^−^, and Fe(II) (Cai et al., 2023; Mehrani et al., 2020; Palomo et al., 2016), *Crenothrix* oxidizing NH_4_^+^, CH_4_, and Fe(II) (Cai et al., 2023; Cheng et al., 2017; Sujith and Bharathi, 2011; Vigliotta et al., 2007), and *Hyphomicrobium*, oxidizing CH_4_ and Fe(II) (Kurt, 2019; Sujith and Bharathi, 2011; Vuilleumier et al., 2011). *Arenimonas* are linked to metal(loid) immobilization via biosorption and mineral formation (Liu J. et al., 2018). Further commonly reported Mn oxidizers, such as *Leptothrix*, *Pedomicrobium*, and *Arthrobacter* (Cai et al., 2023; Carmichael and Bräuer, 2015; Ehrlich and Newman, 2008; Gounot, 1994), were less abundant or absent in the sediments of the Wahnbach Reservoir.

Methanogenesis was consistently present in sediments at all sampling locations and dates, with the highest relative abundances (up to 9.6%) in deeper sediments, particularly at buoy A. *Methanoregula* and *Methanosarcina* were the dominant genera. Although less abundant, *Methanosarcina* species are known to oxidize methane coupled to Mn(IV) reduction (Chen et al., 2023; Ji et al., 2016; Liu et al., 2020). Nitrate respiration showed low relative abundances (≤1.2%), with *Dechloromonas* as the main taxon. Sulfate respiration was more abundant (up to 3.0%), driven by Sva0081 sediment group, *Desulfatiglans*, and *Desulfatirhabdium*. Sulfate respiration occurred in deeper sediment layers. Sequences related to *Desulfobulbus*, *Desulfobacterium,* and *Desulfobacterium* (Holmer and Storkholm, 2001) were less abundant. Notably, *Desulfobulbus* can also reduce Mn(IV) (Lovley and Phillips, 1994). Fe respiration was low (≤0.4%), primarily involving *Desulfuromonas* and *Geobacter*. Mn respiration, as annotated by FAPROTAX, appeared only in three samples at ≤0.01%, and was only represented by the genus *Geobacter*.

Using the list of Mn reducers from our literature research, much higher relative abundances (1.1–8.7%) were found, especially in deeper sediment layers at buoy E, followed by buoy H. Key Mn-reducing genera included *Clostridium*, *Anaeromyxobacter*, *Paenibacillus*, and “*Candidatus* Methanoperedens”. *Clostridium* is mainly fermentative but also capable of metal reduction (Francis and Dodge, 1991; Lovley, 1991; Pyne et al., 2014; Shoiful et al., 2020), and *Anaeromyxobacter* is known for CH_4_ and NH_4_^+^ oxidation and dissimilatory metal-reduction (Liu et al., 2023; Liu et al., 2020; Wang et al., 2020). *Paenibacillus* is associated with N_2_ fixation, PO_4_^3−^ solubilization, and Fe(III) reduction (Ehrlich and Newman, 2008; Grady et al., 2016). The archaeon “*Candidatus* Methanoperedens” oxidizes CH_4_ coupled to NO_3_^−^ and Fe(III) reduction (Leu et al., 2020; Liu et al., 2020; Su et al., 2020). Other known Mn reducers, including *Geothrix*, *Geobacter*, and *Rhodoferax* (Coates et al., 1999; Finneran et al., 2003; Liu et al., 2023; Lovley et al., 1993), were present in lower abundances in the sediments of the Wahnbach Reservoir.

Prokaryotes with Mn-reducing and/or -oxidizing capacity, identified through our literature research, were widespread across all sampling locations, depths, and sampling dates, comprising 2.1–25.0% of the community, with *Bacillus* and *Ellin6067* as dominant taxa. *Bacillus* use organic compounds in aerobic respiration and can affect metal cycling through biosorption, metal cation oxidation, and metal oxide reduction (Francis et al., 2002; Kanso et al., 2002), and *Ellin6067* is known to couple NH_4_^+^ oxidation to Mn(IV) reduction (Jiang Z. et al., 2024; Liu et al., 2023). Other Mn-transforming genera, such as *Shewanella* and *Pseudomonas* (Cai et al., 2023; Gounot, 1994; Lovley, 1991), were less abundant or absent in the sediments of the Wahnbach Reservoir.

### 3.4 Most probable number (MPN) of Mn-transforming microorganisms and community composition of selected cultures

The culture-based approach provided semi-quantitative estimates of active Mn-transforming microbes (Supplementary Table S4). No visible brown coloration developed during the six-week incubation, indicating the absence of detectable lithotrophic Mn oxidizers under the tested conditions. Organotrophic Mn oxidizers were initially scarce (<100 cells mL^−1^ in July) but increased notably by October, reaching 10^3^–10^5^ cells mL^−1^ at buoys A and E, with a maximum of 5.6 · 10^4^ cells mL^−1^ in surface sediments at buoy A. Mn-reducing prokaryotes were more abundant overall (10^2^ to 10^5^ cells mL^−1^), particularly at buoys E and H in July, peaking at 4.3 · 10^4^ cells mL^−1^ in surface sediments at buoy H.

**Figure 7:**
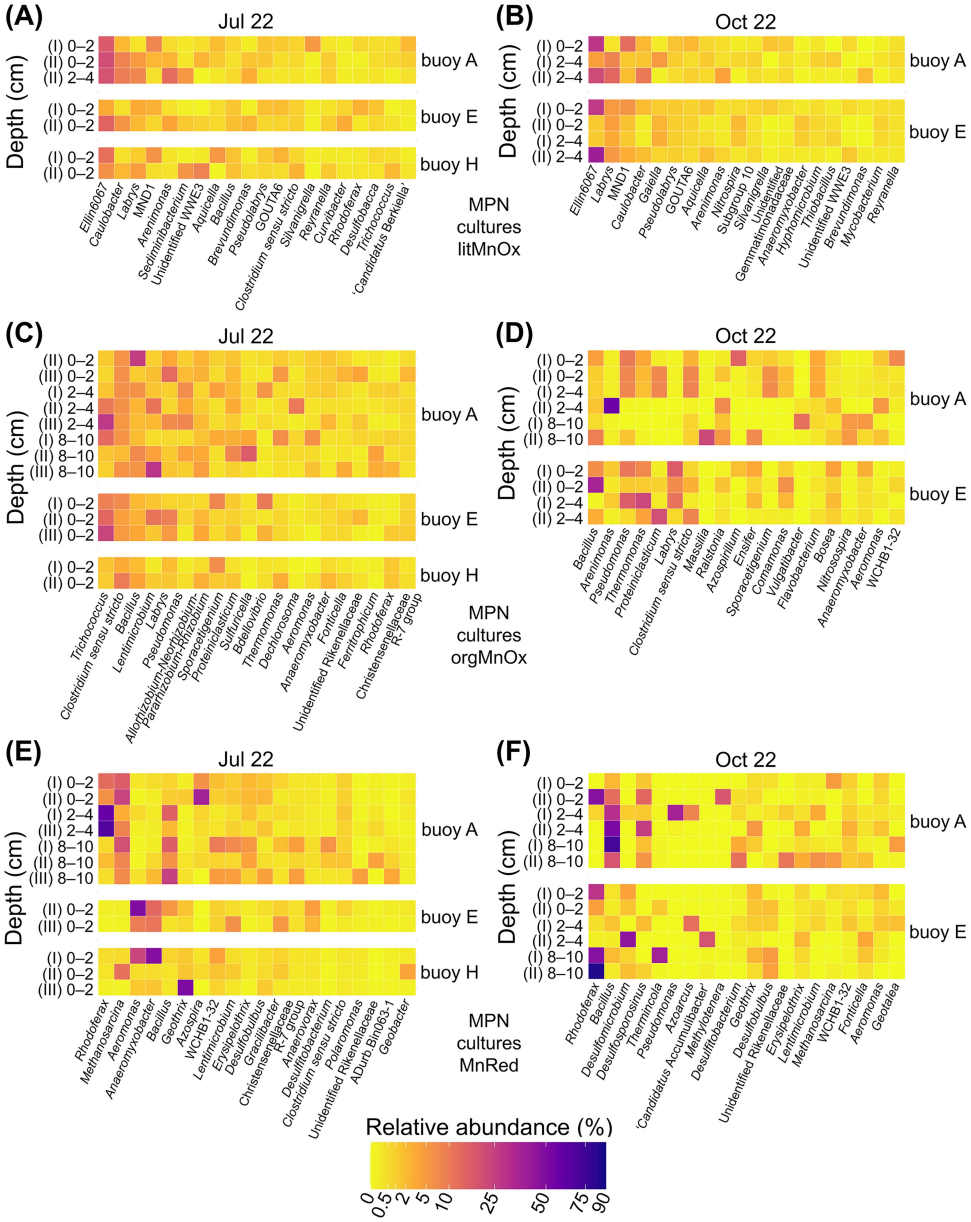
Relative abundance (%) of the twenty most abundant genera enriched in MPN cultures for Mn-transforming microorganisms based on 16S rRNA gene sequencing. Comparison across three sampling locations. Panels show **(A, B)** litMnOx, **(C, D)** orgMnOx, and **(E, F)** MnRed. MPN series were inoculated with different sediment layers (cm). Roman numerals label different dilutions of the MPN series (I, II, III). MPN series were performed with sediments from buoys A, E, and H in July and from buoys A and E in October.

The nMDS plots illustrate patterns of similarity and divergence between prokaryotic communities from enrichment cultures and the original sediment samples. Communities enriched in cultures of lithotrophic Mn-oxidizing microorganisms were generally similar to one another, resembling those of the original sediments, especially in October (Supplementary Figures S6A,B). 16S rRNA gene sequencing identified *Ellin6067*, *Labrys*, MND1, and *Caulobacter* as dominant genera (Figures 7A,B, Supplementary Table S5). Among these, *Ellin6067* and MND1, both abundant in sediments, are known nitrifiers, though only *Ellin6067* has been linked to Mn cycling (Jiang Y. et al., 2024; Liu et al., 2023). In contrast, *Labrys* and *Caulobacter* were enriched in cultures but were rare in sediments. While *Labrys* has no established association with Mn transformation, *Caulobacter* is a well-documented Mn oxidizer (Gounot, 1994; Kurt, 2019).

In contrast to lithotrophic cultures, communities enriched in cultures of organotrophic Mn oxidizers were highly variable and showed limited resemblance to sediment communities (Supplementary Figures S6C,D). 16S rRNA gene sequencing revealed distinct community shifts between July and October (Figures 7C,D, Supplementary Table S5). July cultures were dominated by the Mn reducers *Trichococcus* (Wang et al., 2018) and *Clostridium* (Lovley, 1991; Tebo et al., 2005), the latter also abundant in native sediments. October enrichments varied more and were dominated by *Bacillus* and *Arenimonas*, both of which were already abundant in the original sediment. *Bacillus* is a metabolically versatile genus capable of both Mn oxidation and reduction (Gounot, 1994; Kurt, 2019; Zakharova et al., 2010), while *Arenimonas* is known to perform Mn oxidation (Liu J. et al., 2018; Liu W. et al., 2018). *Pseudomonas*, capable of Mn transformation (Cai et al., 2023; Gounot, 1994; Lovley, 1991), became prominent in enrichments despite being initially low in sediments.

Communities enriched in cultures of Mn reducers formed distinct clusters, clearly separated from native sediment communities (Supplementary Figures S6E,F). Cultures were also grouped by inoculum origin, with nearly complete separation between cultures with sediment from buoys A and E in October. Community composition varied by sampling date and location (Figures 7E,F, Supplementary Table S5). In July, *Rhodoferax* dominated enrichments with sediment from buoy A, followed by *Methanosarcina* as the second most abundant genus. *Bacillus* appeared in deeper layers. In several cultures with sediment from buoys E and H, *Aeromonas* and *Anaeromyxobacter* were dominant, while *Geothrix* was the prevailing genus in one culture with sediment from buoy H. While *Bacillus* and *Anaeromyxobacter* were common in sediments, other dominant taxa were less abundant. *Rhodoferax*, *Methanosarcina*, *Anaeromyxobacter*, and *Geothrix* are all known Mn reducers (Chen et al., 2023; Coates et al., 1999; Finneran et al., 2003; Liu et al., 2023; Lovley et al., 1993), while *Bacillus* has the capacity for both Mn oxidation and reduction (Gounot, 1994; Kurt, 2019; Zakharova et al., 2010). *Aeromonas*, in contrast, is typically associated with Mn oxidation (Ehrlich and Newman, 2008; Shoiful et al., 2020; Zhang et al., 2019). In October, *Bacillus* again dominated enrichment cultures with sediment from buoy A and original sediments, while *Rhodoferax* appeared in several buoy E enrichments but remained rare in the original sediments.

Key metabolisms and the most representative genera identified by FAPROTAX and our own literature research are presented in more detail in Supplementary Figure S7, Supplementary Tables S6, S7.

## 4 Discussion

### 4.1 Redox-controlled Mn and Fe release from the sediment to the water column

In the Wahnbach Reservoir, thermal stratification in summer and autumn (Supplementary Figure S1) leads to O_2_ depletion, with concentrations dropping from ~300 μM to 68 μM at buoy A and to near-anoxic levels (3 μM) at buoy E. Consumption of O_2_ most likely occurred through aerobically respiring organisms, including prokaryotes that use DOC as an electron donor. However, a concomitant decrease of DOC was not observed in bottom waters. In contrast, a slight increase was observed. Natural organic matter, including the dissolved organic matter (DOM) of freshwaters, consists of a huge diversity of organic molecules (Hertkorn et al., 2008) and varies considerably respective composition and reactivity (Dadi et al., 2017; Herzsprung et al., 2012, 2023). DOM adsorbs to mineral phases specifically to Fe (oxyhydr)oxides and to lesser extent to Mn (oxyhydr)oxides (Chorover and Amistadi, 2001; Ma et al., 2020) and may be released by reductive dissolution under anoxic conditions (Dadi et al., 2016, 2017).

Low-oxygen conditions promote Mn release from sediments via microbially mediated Mn oxide reduction (Herschel, 1995; Willmitzer et al., 2015). Dissolved Mn^2+^ is the dominant form in hypolimnetic waters (Godwin et al., 2020; Kamyshny et al., 2025; LaRowe et al., 2021), consistent with findings in water samples at buoy A, where acid-soluble Mn reached 83% Mn^2+^. Reservoir Mn levels vary regionally, with Wahnbach Reservoir values aligning with some studies (LaRowe et al., 2021; WHO, 2021). In many reservoirs, Mn(II) is released from sediments earlier and under less reducing conditions than Fe(II), leading to elevated dissolved Mn concentrations during the onset of stagnation. With prolonged stagnation, however, more reducing conditions favor Fe(III) reduction, and dissolved Fe(II) often surpasses Mn(II) concentrations (Giles et al., 2016; Krueger et al., 2020; Wendt-Potthoff et al., 2014). In addition to sedimentary release, surface runoff and inflow can also represent a relevant source of Fe and Mn input to the water column and should not be neglected in the overall mass balance (Li et al., 2019; Munger et al., 2019). Fe levels in the water column of the Wahnbach Reservoir did not exceed Mn content at any time. Due to its lower redox potential, Fe was released only under stronger reducing conditions (Durn et al., 2001; Lovley, 1995; Lovley and Phillips, 1988), here in sediments at buoy E reaching up to 5.9 μM.

If all Mn(II) present in bottom waters at buoys A and E (9.4 and 24 μM, respectively) were oxidized to Mn(IV), this process could theoretically consume up to 4.7 and 12 μM O_2_. Accordingly, complete reoxidation of Fe(II) to Fe(III) would require an additional 1.5 μM O_2_ in waters above the sediment at buoy E. These values illustrate the potential contribution of inorganic electron donors (Mn^2+^, Fe^2+^) to aerobic respiration, in comparison with the generally much larger O_2_ demand driven by the oxidation of organic carbon (in bottom waters up to 138 μM at buoy A and 161 μM at buoy E). In the natural environment, these processes are highly dynamic with varying turnover rates as Mn(II) and Fe(II) are replenished from the sediment by reduction processes, while O_2_ diffuses downward from overlying oxic water layers. Additionally, the processes by which reoxidation occurs may differ (abiotic vs. biotic). However, O_2_ is the strongest and most common oxidant available; its consumption in natural waters may be shared between abiotic and biotic processes and between chemoorganotrophic (organic C oxidation) and chemolithotrophic processes (oxidation of reduced Mn, Fe, or NH_4_^+^). As indicated here, although inorganic electron donors can contribute to O_2_ consumption, their relative share is generally minor compared with that of organic matter oxidation.

Furthermore, low concentrations of NO_3_^−^ in bottom waters (Supplementary Figure S1N) suggest denitrification. Such depletion is typical in deeper waters above the sediment, where denitrification often represents the dominant pathway of NO_3_^−^ loss, reducing NO_3_^−^ to N_2_ and thereby limiting its accumulation in bottom waters. Together, lower NO_3_^−^ values and Mn/Fe enrichment at the sediment-water interface under low O_2_ conditions and high organic carbon contents point to anaerobic processes such as denitrification and Mn(IV)/Fe(III) reduction (Forsberg, 1989; Hamilton-Taylor and Davison, 1995; Jiang Y. et al., 2024; Zhang D. et al., 2022).

### 4.2 Mn and Fe dynamics in reservoir sediments

Sediments in lakes and reservoirs act as sinks or sources for nutrients and redox-sensitive metals, depending on redox conditions at the sediment-water interface (Forsberg, 1989; Krueger et al., 2020). In Wahnbach Reservoir sediments (Figure 3), Mn reached up to 28 mM (15.6 g kg^−1^) in surface layers at the deepest sampling location (buoy A), likely due to Mn focusing (Scholtysik et al., 2020; Scholtysik et al., 2022). These levels exceed typical freshwater sediment Mn concentrations of 0–7 g kg^−1^ (von Gunten et al., 1997; Krueger et al., 2020; LaRowe et al., 2021; Madison et al., 2013). Usually, Fe exceeds Mn in sediments (Durn et al., 2001; Patrick and Henderson, 1981); however, here, Mn was comparably high. The extraction methods targeted Mn and Fe (oxyhydr)oxides (Durn et al., 2001; Schwertmann and Taylor, 1989), with dithionite and oxalate isolating poorly and well-crystallized Mn oxides, such as manganite, bixbyite, and hausmannite, though oxalate extraction is generally less efficient (Canfield et al., 1993; Durn et al., 2001; Lenstra et al., 2021). For Fe, dithionite extracts secondary oxides while oxalate targets amorphous and poorly crystalline forms (Canfield et al., 1993; Durn et al., 2001; Lenstra et al., 2021). Unexpectedly, oxalate extracts contained more Mn and Fe than dithionite extracts (Figure 3, Supplementary Figures S3, S4). It also has to be noted that in our study, extracts also included Mn(II) and Fe(II) forms such as carbonates, acid volatile sulfides, and dissolved Mn^2+^/Fe^2+^ (Canfield et al., 1993; Poulton and Canfield, 2005).

Redox potentials (Figure 2) suggest that Mn in sediments predominantly exists as Mn(IV) oxides, since Mn(IV) reduction occurs at potentials below +300 to +200 mV (Gotoh and Patrick, 1972), while Fe(III) reduction requires more reducing conditions below +100 mV to −47 mV (Durn et al., 2001; Patrick and Henderson, 1981). However, depending on the mineral phases present, the redox potential ranges for Fe and Mn minerals, leading to zones where Mn(IV) and Fe(III) reduction and also other anaerobic respiration processes can occur (Fiskal et al., 2019; Liu J. et al., 2022; Lovley, 1991; Lovley et al., 2004). Comparisons of redox potential profiles in the sediment between sampling locations suggest localized heterogeneity, whereas temporal differences between sampling dates are likely linked to seasonal biogeochemical changes. Organic matter present in the sediment of the Wahnbach Reservoir most likely served as electron donor for the anaerobic respiration processes.

Pore water concentrations of dissolved Mn^2+^ and Fe^2+^ (Figures 4B,C) in sediments at buoy A were generally low and made up only a small fraction of total Mn and Fe extracted by dithionite or oxalate. In September, concentrations of dissolved Mn and Fe were higher in deeper layers, with Mn^2+^ exceeding Fe^2+^, suggesting Mn(IV) and Fe(III) reduction to occur under reducing conditions, probably fueled by organic carbon degradation. The resulting Mn^2+^ and Fe^2+^ diffuse upward, where they may be reoxidized and redeposited, contributing to metal focusing. The biotically oxidation of Mn^2+^ can be very fast, so Mn^2+^ does not accumulate (Morgan, 2005). Mn^2+^ pore water levels in Wahnbach Reservoir reached up to 276 μM, placing them at the higher end of values reported for freshwater systems (LaRowe et al., 2021).

The NH_4_^+^ profile (Figure 4G) in the pore water showed higher concentrations of NH_4_^+^ at greater depths. Degradation of organic matter and concomitant ammonification may be a source of NH_4_^+^. It indicates a consumption of ammonification in the surface sediment layer and/or overlying water. At the sediment surface, the observed decrease in NH_4_^+^ is a result of nitrification, while the resulting NO_3_^−^ is subsequently consumed through denitrification, and a smaller fraction of NO_3_^−^ is likely reduced to NH_4_^+^ via nitrate ammonification (Cojean et al., 2020). Therefore, low concentrations of NO_3_^−^ (Figure 4F) were observed in the pore water. The process of nitrification can occur even at low O_2_ concentrations (Stenstrom and Poduska, 1980), with Mn oxides serving as electron acceptors, particularly in the upper sediment layers (Hulth et al., 1999). Additionally, in deeper sediment layers, SO_4_^2−^ depletion (Figure 4D) indicates SO_4_^2−^reduction. In the Wahnbach Reservoir, pH slightly decreased below 7 at the sediment-water interface, reaching lower values than in the overlying water column and the deeper sediment layers. This decrease is likely caused by the reoxidation of reduced inorganic compounds, including sulfide (reflected in elevated SO_4_^2−^ concentrations at the sediment surface), Fe(II), and Mn(II) compounds. With depth, pH gradually increases, reflecting ongoing reductive processes in the sediments (Silburn et al., 2017).

Sediment and pore water profiles reflect a typical redox zonation in the Wahnbach Reservoir: denitrification in the sediment-water interface, as well as nitrification in surface sediments, followed by Mn(IV), Fe(III), and SO_4_^2−^ reduction with increasing depth (Canfield and Thamdrup, 2009). Estimated potential electron acceptor concentrations are: (i) Fe(III) up to 74 mM (assuming that most of the extracted Fe is accounted by Fe oxides), (ii) Mn(IV) up to 28 mM (assuming that most of the extracted Mn is accounted by Mn oxides), (iii) SO_4_^2−^ up to 438 μM, and (iv) NO_3_^−^ < 1 μM, whereby the high NH_4_^+^ concentrations (increasing to 127 μM) indicate production from organic matter degradation, with NO_3_^−^ appearing only transiently as an intermediate and being rapidly consumed through denitrification. Organic carbon will be one of the most important electron donors in this context.

### 4.3 Key prokaryotic players driving redox processes in sediments

The dominant prokaryotic classes in Wahnbach Reservoir sediments (Supplementary Figure S5) were consistent across sampling locations and dates, typical of freshwater lakes and sediments (Huang et al., 2017; Newton et al., 2011; Song et al., 2024; Wu et al., 2017; Zemskaya et al., 2021; Zhang et al., 2019). The distribution of the community likely reflects general redox zonation and/or organic carbon availability. More detailed insights into metabolic processes were obtained from 16S rRNA gene sequencing results at the genus level, which reflected redox changes and were supported by physicochemical data, consistent with typical patterns in freshwater sediments (Canfield and Thamdrup, 2009).

Although Mn and Fe concentrations were high in sediments, especially at buoy A, and elevated Mn^2+^ and Fe^2+^ levels in pore water suggested Mn(IV) and Fe(III) reduction, FAPROTAX detected few sequences linked to Fe-transforming prokaryotes and almost none for Mn-transformers (Figure 6). This discrepancy highlights a common limitation of predictive functional tools, which rely on curated reference databases and cultured representatives (Djemiel et al., 2022; Sansupa et al., 2021). Many genera with experimentally confirmed Mn-transforming ability, such as *Clostridium*, *Bacillus*, or *Anaeromyxobacter*, are not explicitly annotated in FAPROTAX, which may result in their underrepresentation. Literature research revealed that Mn-transforming taxa were far more widespread than suggested by the automated annotation.

Despite their apparently modest relative abundances, Mn- and Fe-transforming prokaryotes can play disproportionally large roles in sediment redox dynamics (Canfield et al., 2005; Lovley, 2006). Both metals often act as electron acceptors in anoxic environments, for example, facilitating the oxidation of organic carbon, and serve as redox partners in the reduction of NO_3_^−^ and SO_4_^2−^ or during methanogenesis. Importantly, many Mn-associated prokaryotes in sediments of the Wahnbach Reservoir are not restricted to metal cycling but are metabolically versatile, participating simultaneously in other elemental cycles. Such multiple metabolic roles suggest that Mn cycling in freshwater sediments cannot be understood in isolation but must be considered in a broader Mn-Fe-S-N-C network.

Depth-resolved patterns reflect classical redox zonation in sediments, where Mn and Fe reduction typically occur beneath the zone of declining O_2_ concentrations and low NO_3_^−^ availability, followed by SO_4_^2−^ reduction and methanogenesis (Berg et al., 1998; Canfield and Thamdrup, 2009; Fiskal et al., 2019). Observed substantial Mn-reducing genera alongside only moderate abundances of SO_4_^2−^ reducers suggests that Mn and Fe play a stronger role in controlling anaerobic organic matter turnover in sediments of the Wahnbach Reservoir compared to SO_4_^2−^. This is consistent with the relatively high organic matter content (~5–10%), which supports anaerobic degradation and methane production. While no visible methane release was observed, Mn cycling may suppress methanogenesis, as suggested in previous studies (Ma et al., 2008). Organisms associated with nitrification were relatively abundant, indicating that NH_4_^+^ produced in deeper layers is actively metabolized in surface sediment. Although organisms linked to NO_3_^−^ respiration were less abundant, this process nonetheless plays an important role in shaping sediment biogeochemistry, as discussed above. The ecological relevance of Mn cycling has broader implications (Wang et al., 2022). Freshwater reservoirs often rely on the cycling of Mn and also Fe to regulate carbon turnover and CH_4_ production (Leu et al., 2020; Ma et al., 2008; Su et al., 2020). In marine sediments, SO_4_^2−^ reducers often dominate and suppress methanogenesis (Su et al., 2020), rather than other electron acceptors, such as NO_3_^−^, Mn, and Fe compounds.

### 4.4 Mn-transforming prokaryotes enriched in MPN cultures

Coloration and discoloration of MPN cultures, especially at higher dilutions, indicated biotic Mn oxidation and reduction. Prokaryotes abundant at higher dilutions likely thrived under selective conditions and may contribute to Mn cycling in Wahnbach Reservoir sediments, as observed for Mn reducer cultures. No clear growth was observed in lithotrophic Mn oxidizer cultures, whereas organotrophic Mn oxidizers peaked at the deepest sampling location, where Mn and organic matter were abundant (Supplementary Table S4). Mn reducers were consistently detected at all sampling locations, with MPN around 10^3^–10^4^ mL^−1^, comparable to values in the Rappbode Reservoir, Germany (Wendt-Potthoff et al., 2014).

In lithotrophic Mn oxidizer cultures, minimal Mn oxidation and Mn-dependent growth were observed, even at the lowest dilution. Thus, extracted DNA (Figures 7A,B, Supplementary Tables S5–S7) likely came from sediment organisms such as *Ellin6067*, possibly growing on sediment-derived substrates with little influence from Mn. Enriched organisms, such as *Caulobacter*, an aerobic chemoorganotroph (Abraham et al., 1999), likely utilized organic matter from the sediment (Gounot, 1994; Kurt, 2019). Although it appeared to have a limited impact in the original sediments, it derived an advantage for growth from enrichment cultures for Mn oxidizers. Overall, lithotrophic Mn oxidizers likely play a minor role in Mn cycling in the Wahnbach Reservoir.

In organotrophic Mn oxidizer cultures (Figures 7C,D, Supplementary Tables S5–S7), a diverse community was enriched, including known Mn-oxidizing and -reducing prokaryotes. Abundant and enriched Mn oxidizers, such as *Arenimonas* (Liu et al., 2018), might play a major role here, whereby Mn oxidizers such as *Thermomonas* (Li et al., 2024; Yang et al., 2014) were also enriched, which, due to their low abundance in the original sediment, are probably not of great importance for the Mn cycle in the Wahnbach Reservoir, but showed a high potential for active Mn oxidation. The same apply to the enriched Mn-transforming organisms *Bacillus* (Gounot, 1994; Kurt, 2019; Zakharova et al., 2010), already abundant in original sediments, and *Pseudomonas* (Gounot, 1994; Tebo et al., 2004; Zakharova et al., 2010), previously detected in low abundance. Their enrichment and associated medium discoloration suggest a role in Mn oxidation. The enrichment of genera that are already quite abundant in sediments demonstrates their relevance for active Mn cycling in the Wahnbach Reservoir. Surprisingly, the typical Mn reducers *Trichococcus* (Wang et al., 2018) and *Clostridium* (Lovley, 1991; Tebo et al., 2005) were observed. *Clostridium*, already abundant in sediments, likely persisted due to low dilution, while *Trichococcus*, a facultative anaerobe, may have thrived on available organic matter. The enriched community reflects adaptation to varied conditions.

In Mn reducer cultures (Figures 7E, F, Supplementary Tables S5–S7), various prokaryotes capable of Mn reduction or both Mn oxidation and reduction were enriched. Due to higher dilutions, these cultures were less influenced by the original sediment community and exhibited clear Mn(IV) reduction, as indicated by discoloration. *Anaeromyxobacter*, abundant in sediments, was enriched in cultures from buoys E and H, supporting its role in active Mn reduction (Liu et al., 2023; Liu et al., 2020; Wang et al., 2020), potentially coupled to anaerobic CH_4_ oxidation in the original sediments. *Rhodoferax*, initially less abundant, was strongly enriched, benefiting from the organic-rich medium while reducing Mn(IV) and Fe(III) (Finneran et al., 2003; Kato and Ohkuma, 2021). *Methanosarcina* enrichment in cultures from buoy A, where it was already present, suggests involvement in anaerobic CH_4_ oxidation coupled to Mn reduction (Chen et al., 2023; Liu et al., 2020; Yu et al., 2022) in the Wahnbach Reservoir sediments. *Azospira*, initially rare, was enriched in upper layer cultures from buoy A, indicating Mn’s relevance in NO_3_^−^ respiration (Peng et al., 2016; Tong et al., 2023). Genera such as *Desulfomicrobium*, *Desulfosporosinus*, and *Desulfitobacterium* (Kim et al., 2012; Sharak Genthner and Devereux, 2015; Thevenieau et al., 2007; Villemur et al., 2006), previously less abundant, were also enriched, likely coupling organic oxidation with Mn(IV) reduction. These findings underscore the abundance and diversity of Mn-reducing microbes, their wide repertoire of utilizing and linking different metabolic pathways, and the significance of Mn reduction in the Wahnbach Reservoir.

The enrichment of genera enriched in both cultures of Mn reducers and (organotrophic) Mn oxidizers, along with the presence of prokaryotes exhibiting various Mn-associated metabolisms in the sediment, highlighted the adaptability of the prokaryotic community in the Wahnbach Reservoir. Additionally, the enrichment of genera previously in low abundances in sediments suggests that under more reducing conditions, Mn reduction could increase, leading to greater Mn release into the water column of the Wahnbach Reservoir.

## 5 Conclusion

The metabolic pathways identified in this study reveal a complex and tightly interconnected biogeochemical network in the Wahnbach Reservoir, linking the cycling of Mn, Fe, N, S, and C (Cojean et al., 2020; Gounot, 1994; Hulth et al., 1999; Pimenov and Neretin, 2006; Su et al., 2020; Thamdrup et al., 2000; Wallenius et al., 2021). Key prokaryote-mediated processes shaping this network include: (i) Mn(IV) and Fe(III) reduction primarily in deeper sediment layers, with dissolved Mn(II) and Fe(II) largely retained and reoxidized in surface layers; (ii) probably ammonification in deeper layers coupled to nitrification and denitrification in upper layers and the sediment-water interface, (iii) sulfate reduction occurring in deeper, anoxic zones; and (iv) potential methane production in the deeper sediments, counterbalanced by CH_4_ oxidation in the surface sediments. These processes align with established redox stratification patterns commonly found in freshwater systems (Berg et al., 1998; Canfield and Thamdrup, 2009; Fiskal et al., 2019).

The presence of Mn in the water column reflects episodic shifts toward more reducing conditions, often during stratification or stimulated organic carbon production. In the Wahnbach Reservoir, sediment processes regulate which reduced compounds are released or retained. In particular, in-sediment reoxidation of reduced compounds appears to act as a critical barrier to the release of NH_4_^+^, partially Fe(II), H_2_S/HS^−^, and CH_4_ into the overlying water. Simultaneously, the regeneration of Mn(IV), Fe(III), NO_3_^−^, and SO_4_^2−^, which act as alternative terminal electron acceptors, sustain redox cycling within the sediment. Importantly, NO_3_^−^ and SO_4_^2−^ can also be replenished in the absence of O_2_ and contribute significantly due to high turnover. Mn(IV) reduction emerges as a pivotal process, supported by the availability of Mn(IV) in sediments and the presence of prokaryotes capable of coupling its reduction with the oxidation of NH_4_^+^, S compounds, organic matter, and CH_4_ (Godwin et al., 2020; Gounot, 1994; Lovley, 1991; Wang et al., 2022).

Our results, therefore, extend the general consensus on redox zonation and metal accumulation by emphasizing the regulatory role of sediments as both sources and sinks. In contrast to much of the literature, where Mn and Fe are often discussed together, our data demonstrate that their roles and dynamics differ substantially. Although some microorganisms are capable of reducing both Mn and Fe, many are specialized, and their activities are further separated by the markedly different redox potentials required for Mn(IV) and Fe(III) reduction. Moreover, predictive tools such as FAPROTAX underestimate several taxa with confirmed Mn- or Fe-transforming capacities, leading to an underappreciation of their ecological relevance. In the Wahnbach Reservoir, Mn cycling in particular emerges as disproportionately important: Mn transformation is a key pathway tightly linked to N, S, and C transformations (Wang et al., 2022). This coupling makes Mn a central node in controlling water column chemistry and, by extension, drinking water quality.

Redox dynamics are particularly important for drinking water management. Sustained reducing conditions can enhance the mobilization of Mn(II), necessitating additional treatment efforts. In the Wahnbach Reservoir, differences between sampling locations highlight the central role of local redox conditions, as overall O_2_ concentrations, or their absence, represent one of the most important predictors of Mn release and accumulation. Since input from surface runoff or atmospheric deposition can be largely ruled out here, Mn cycling is primarily governed by in-sediment processes. Mn reduction sets in rapidly once O_2_ levels decline, and the rates of biotic reduction depend on both the content and type of Mn minerals, the availability of electron donors (including organic carbon, NH_4_^+^, and CH_4_), and the ecophysiology of the microbial community. Equally important are reoxidation processes, which strongly affect the net accumulation of Mn in bottom waters; abiotic pathways, in particular, can play a key role (Ma et al., 2020). Thus, continuous monitoring of redox-sensitive parameters in the Wahnbach Reservoir is essential to assess the potential for Mn release, its dependency on local redox changes, and the underlying microbial and geochemical mechanisms driving Mn cycling. Understanding these interactions is key to anticipating and mitigating water quality challenges under changing environmental conditions.

## Data Availability

The datasets presented in this study are publicly available. This data can be found at: https://www.ncbi.nlm.nih.gov/sra, accession number PRJNA1263398.

## Footnotes

^1^ https://www.wahnbach.de
