# Supplementary material for: Anaerobic prokaryotic processes drive manganese release in a drinking water reservoir
Source: Front Microbiol. 2025 Oct 15;16:1671749. doi: 10.3389/fmicb.2025.1671749 (PMC12569786; doi:10.3389/fmicb.2025.1671749)
Supplement: Supplementary file 1 [file Supplementary_file_1.docx]

Supplementary Material

# Supplementary Figures and Tables

## Supplementary Figures


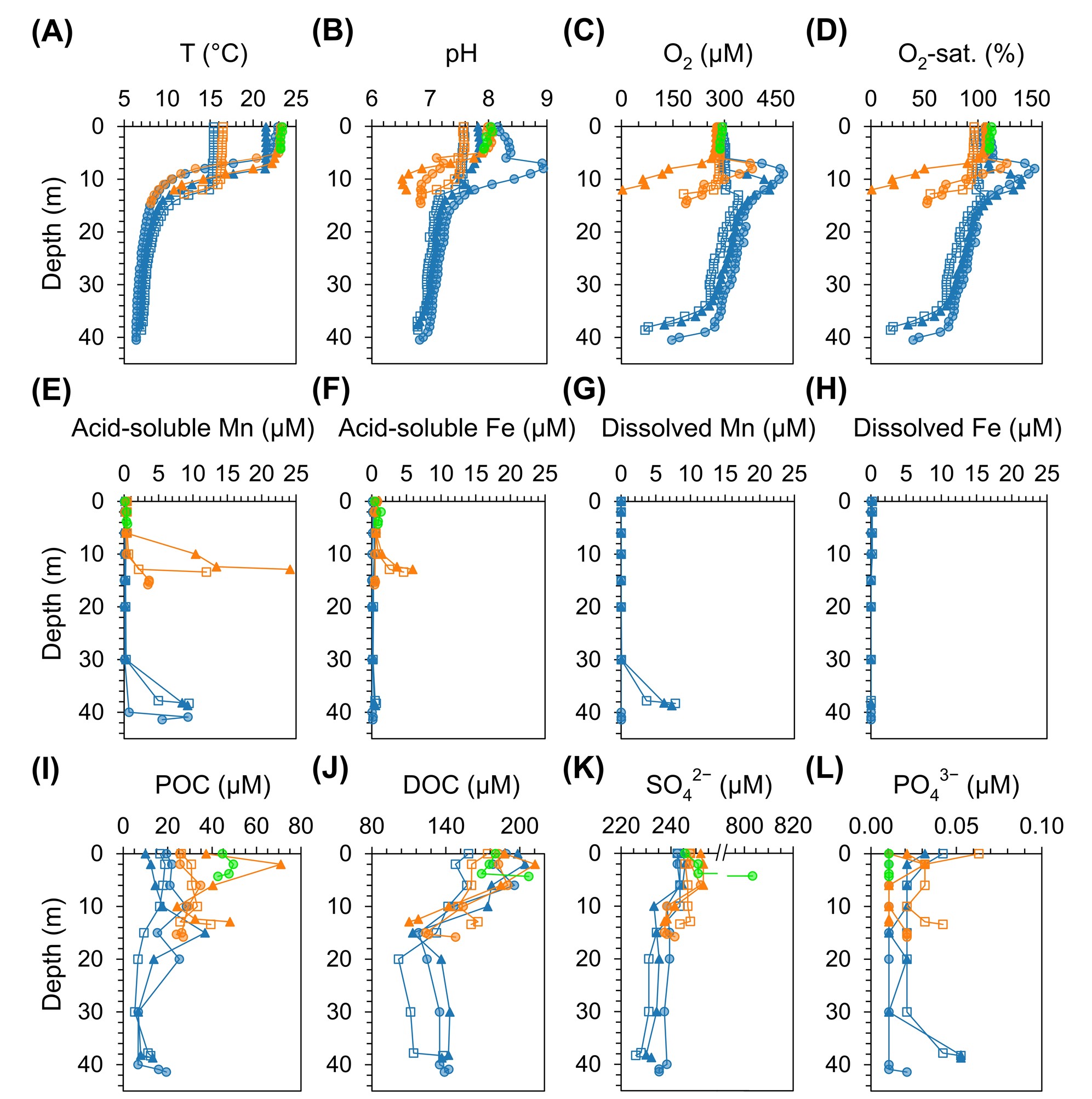


*(Continued)*


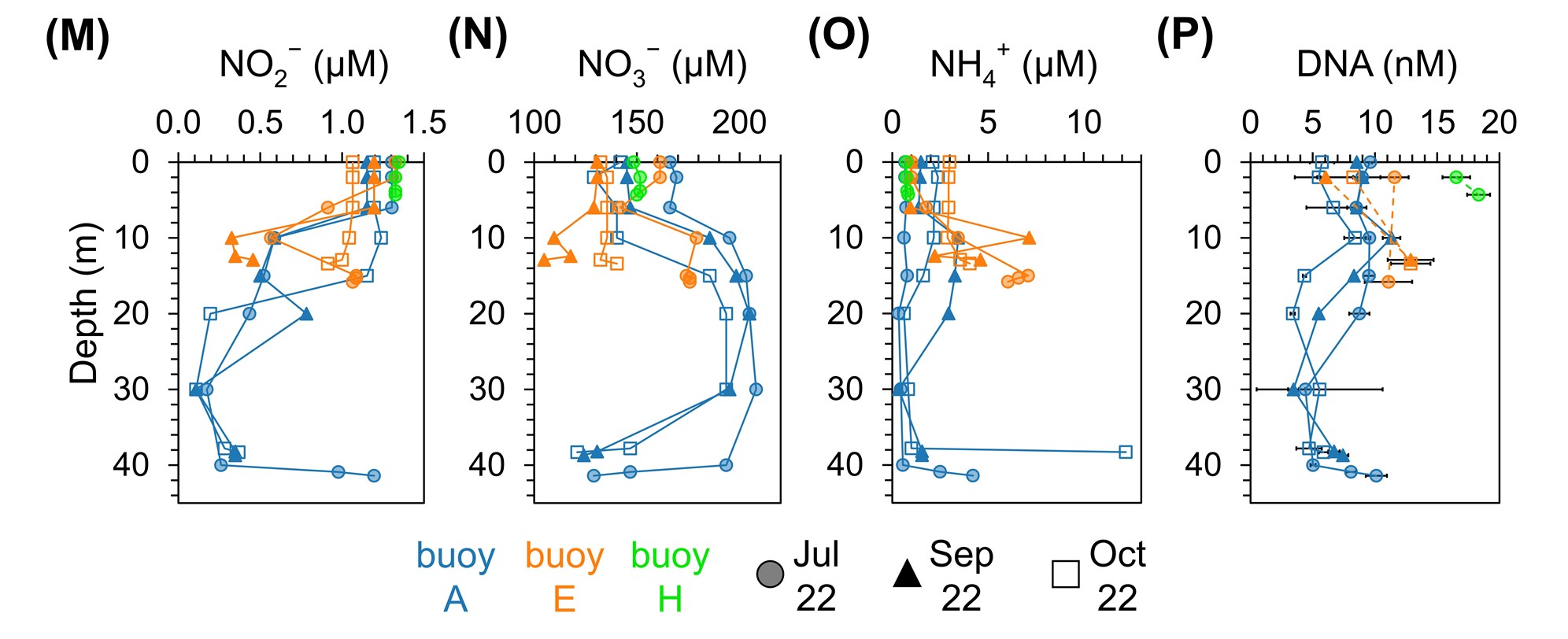


**Supplementary Figure S1.** Physicochemical parameters of the water column. Comparison across three sampling locations, partwise for samplings in July (shaded circle), September (filled triangle), and October (empty square): buoy A (blue), buoy E (orange), and buoy H (green) in the water depth profile (m). **(A)** Temperature (°C), **(B)** pH, **(C)** O_2_ concentration (µM), **(D)** O_2_ saturation (%), **(E)** acid-soluble Mn (µM), **(F)** acid-soluble Fe (µM), **(G)** dissolved Mn (µM), **(H)** dissolved Fe (µM), **(I)** POC (µM), **(J)** DOC (µM), **(K)** SO_4_^2−^ (µM), **(L)** PO_4_^3−^ (µM), **(M)** NO_2_^−^ (µM), **(N)** NO_3_^−^ (µM), **(O)** NH_4_^+^ (µM), and **(P)** DNA (nM).

**
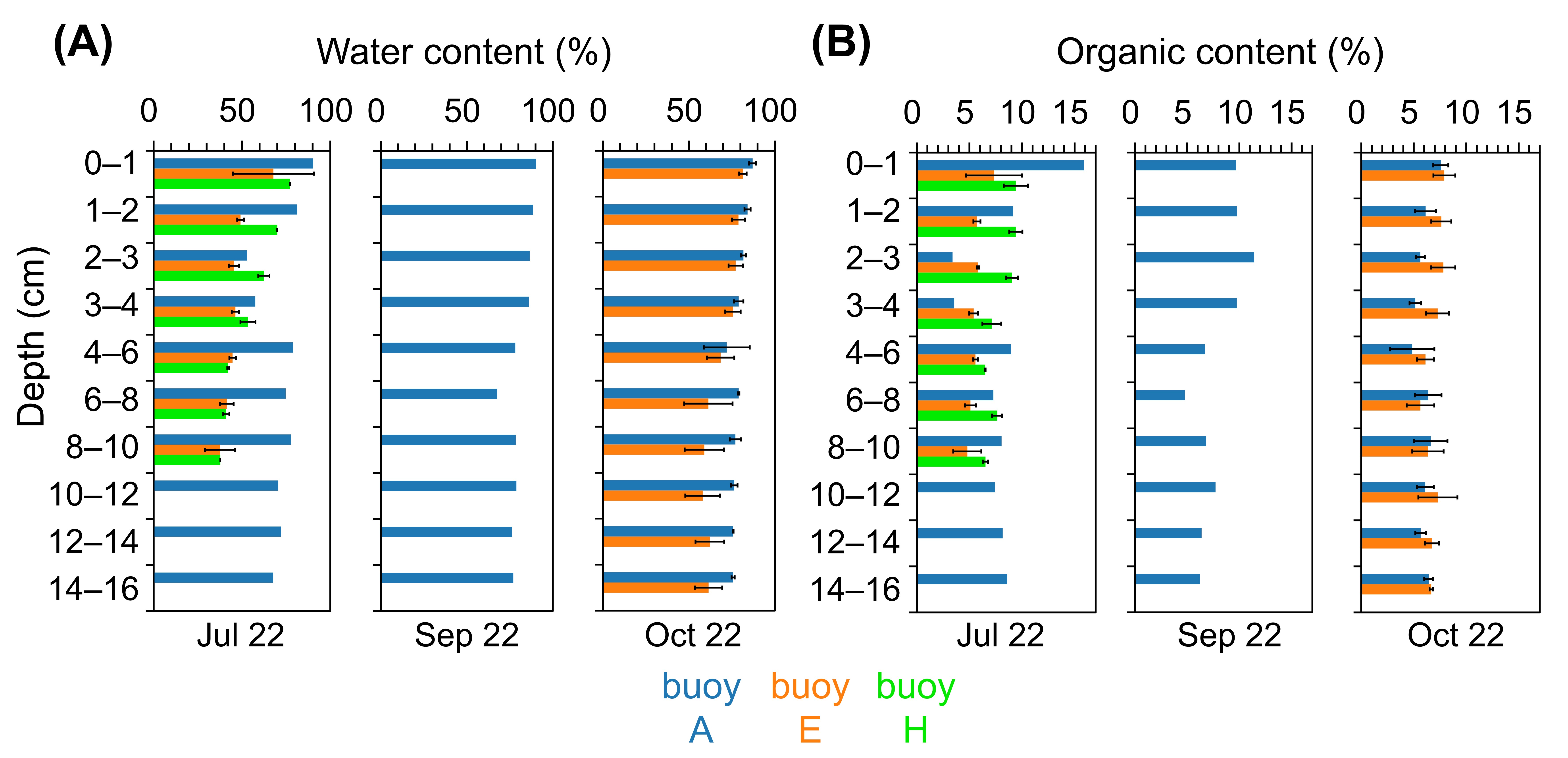
**

**Supplementary Figure S2.** Water content (%) and organic content (%) of sediment layers (cm). Comparison across three sampling locations, partwise for samplings in July, September, and October: buoy A (blue), buoy E (orange), and buoy H (green). **(A)** Water content (%) determined by loss-on-drying, and **(B)** organic content (%) determined by loss-on-ignition.


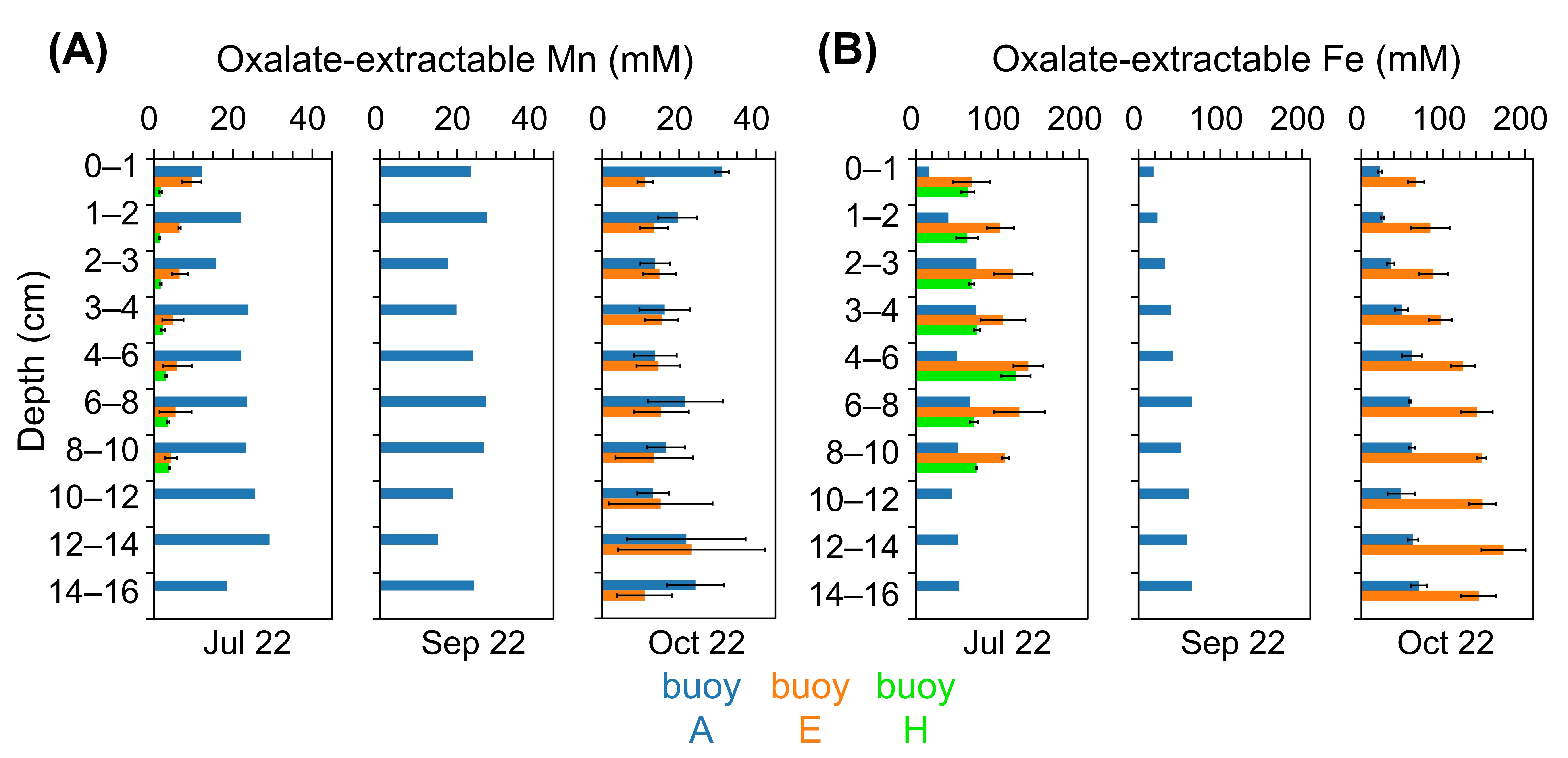


**Supplementary Figure S3.** Oxalate-extractable Mn and Fe contents (mM) of sediment layers (cm). Comparison across three sampling locations, partwise for samplings in July, September, and October: buoy A (blue), buoy E (orange), and buoy H (green). **(A)** Mn and **(B)** Fe concentrations (mM).


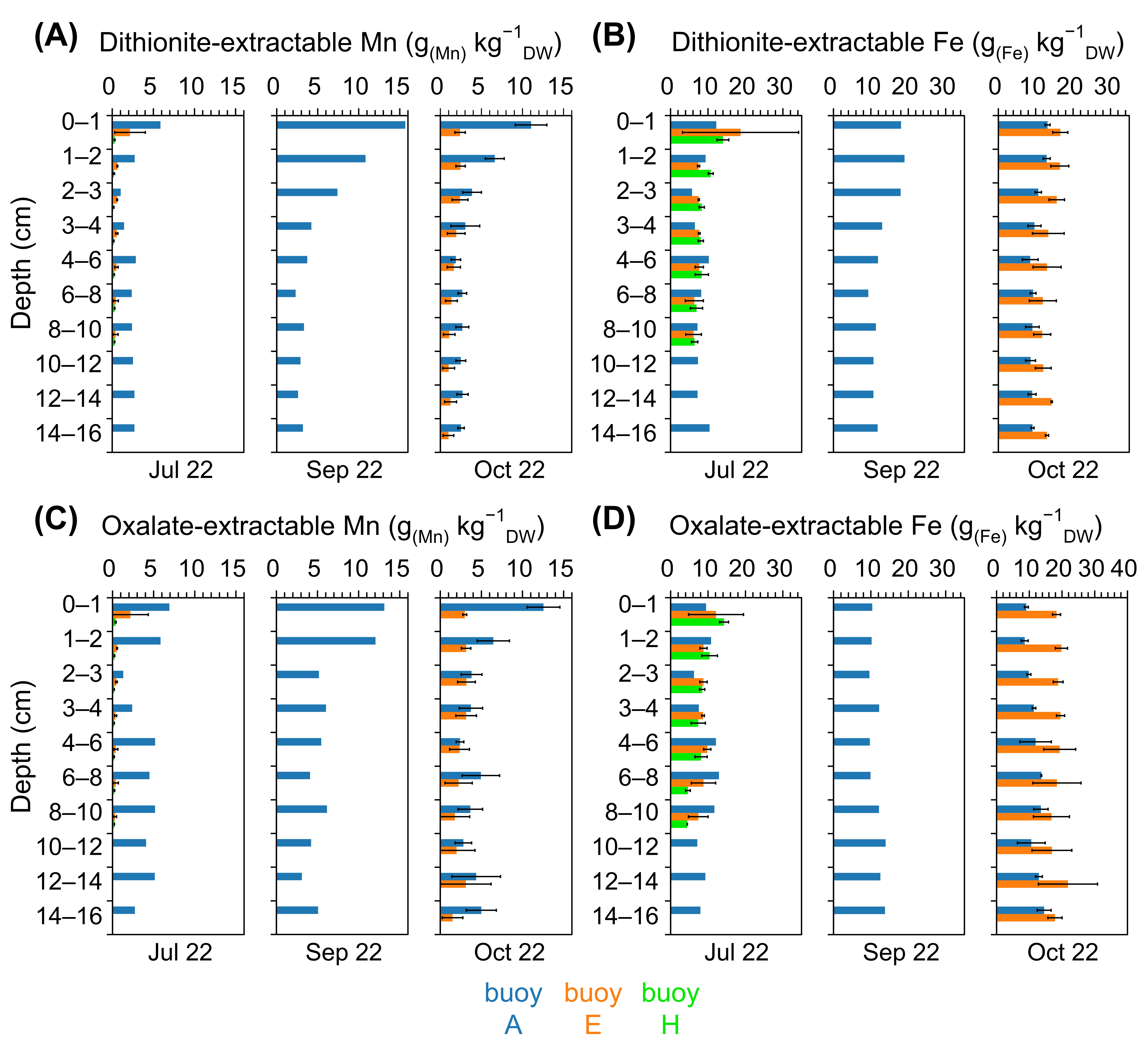


**Supplementary Figure S4.** Mn and Fe contents (g kg^−1^_DW_) of sediment layers (cm). Comparison across three sampling locations, partwise for samplings in July, September, and October: buoy A (blue), buoy E (orange), and buoy H (green). Dithionite-extractable **(A)** Mn and **(B)** Fe, and oxalate-extractable **(C)** Mn and **(D)** Fe contents (g kg^−1^_DW_).


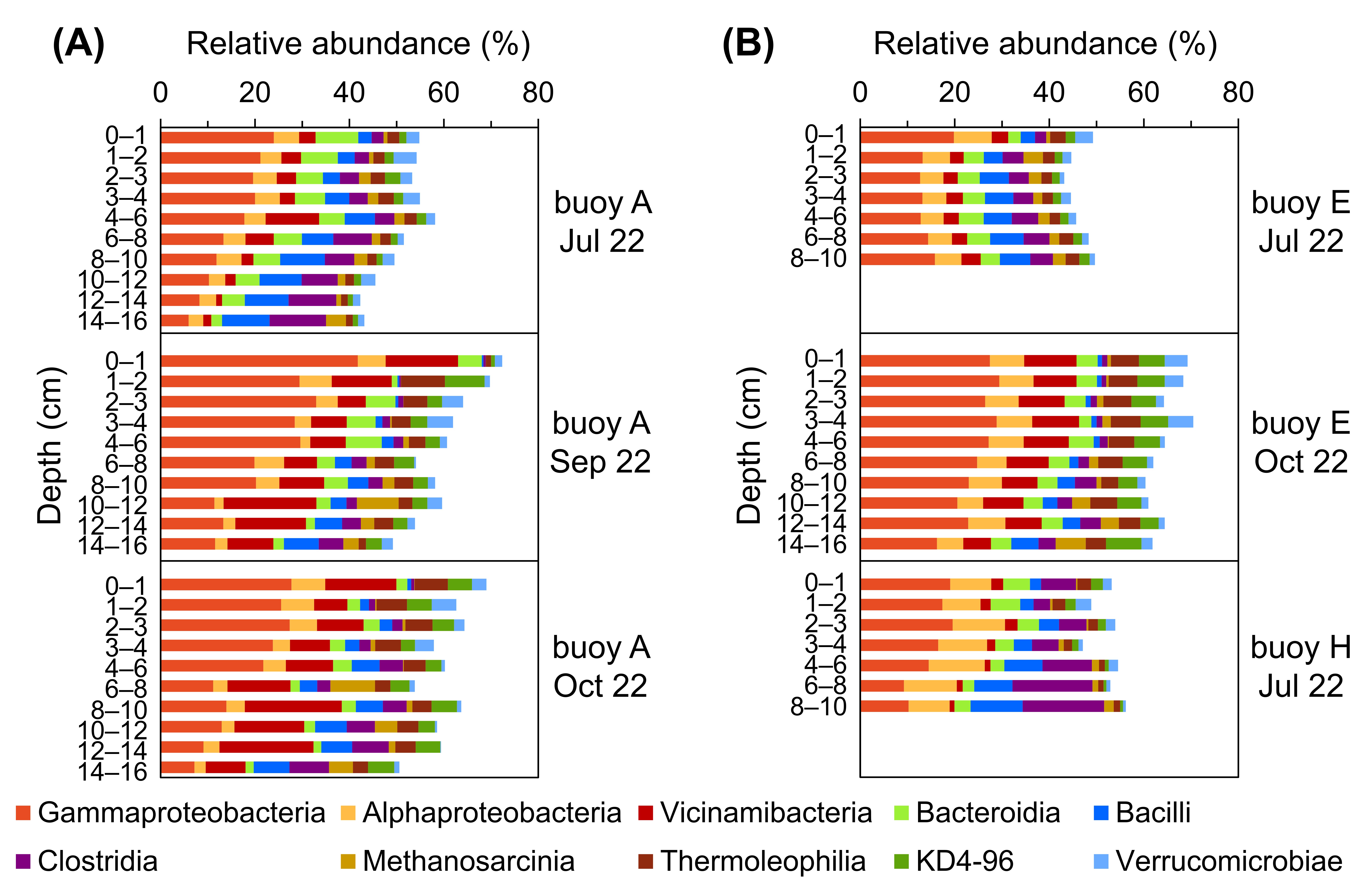


**Supplementary Figure S5.** Relative abundance (%) of 16S rRNA gene sequences for the ten most abundant classes of microorganisms in sediment layers (cm). Comparison across three sampling locations. **(A)** For the sampling at buoy A in July, September, and October. **(B)** For the sampling at buoy E in July and October, and at buoy H in July.


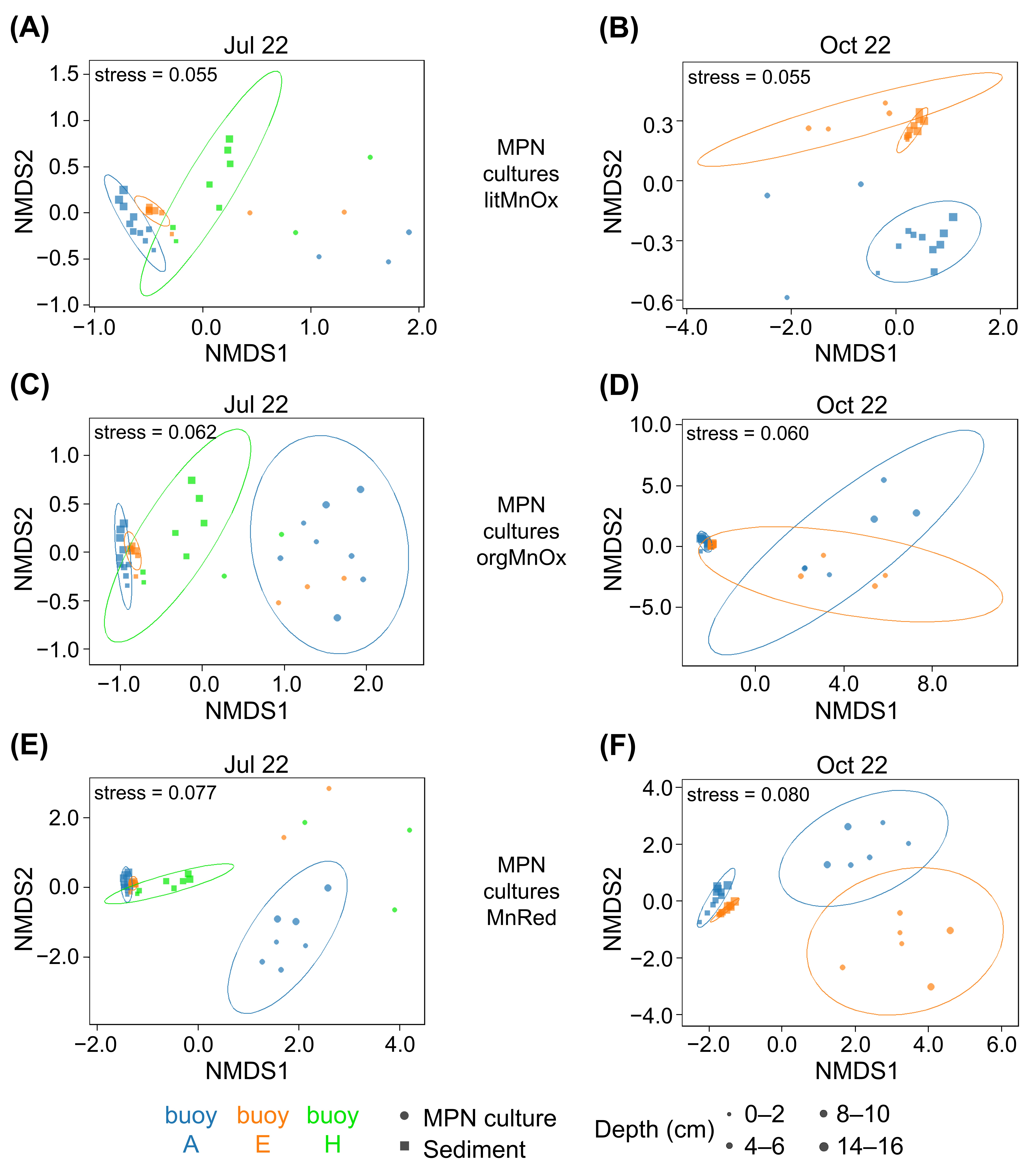


**Supplementary Figure S6.** The nMDS plots of OTU/ASV distribution of MPN cultures in comparison to sediment core samples based on 16S rRNA gene sequences. Comparison across the three sampling locations of buoy A (blue), buoy E (orange), and buoy H (green). Symbol size represents sediment depth (cm). Sediment samples (square) and MPN cultures (circle) are shown in a single graph for **(A, B)** litMnOx, **(C, D)** orgMnOx, and **(E, F)** MnRed. Cultivation results from July and October. Core microbiota indicated by ellipses based on multivariate t-distribution with a 95% confidence interval. Stress values are provided.


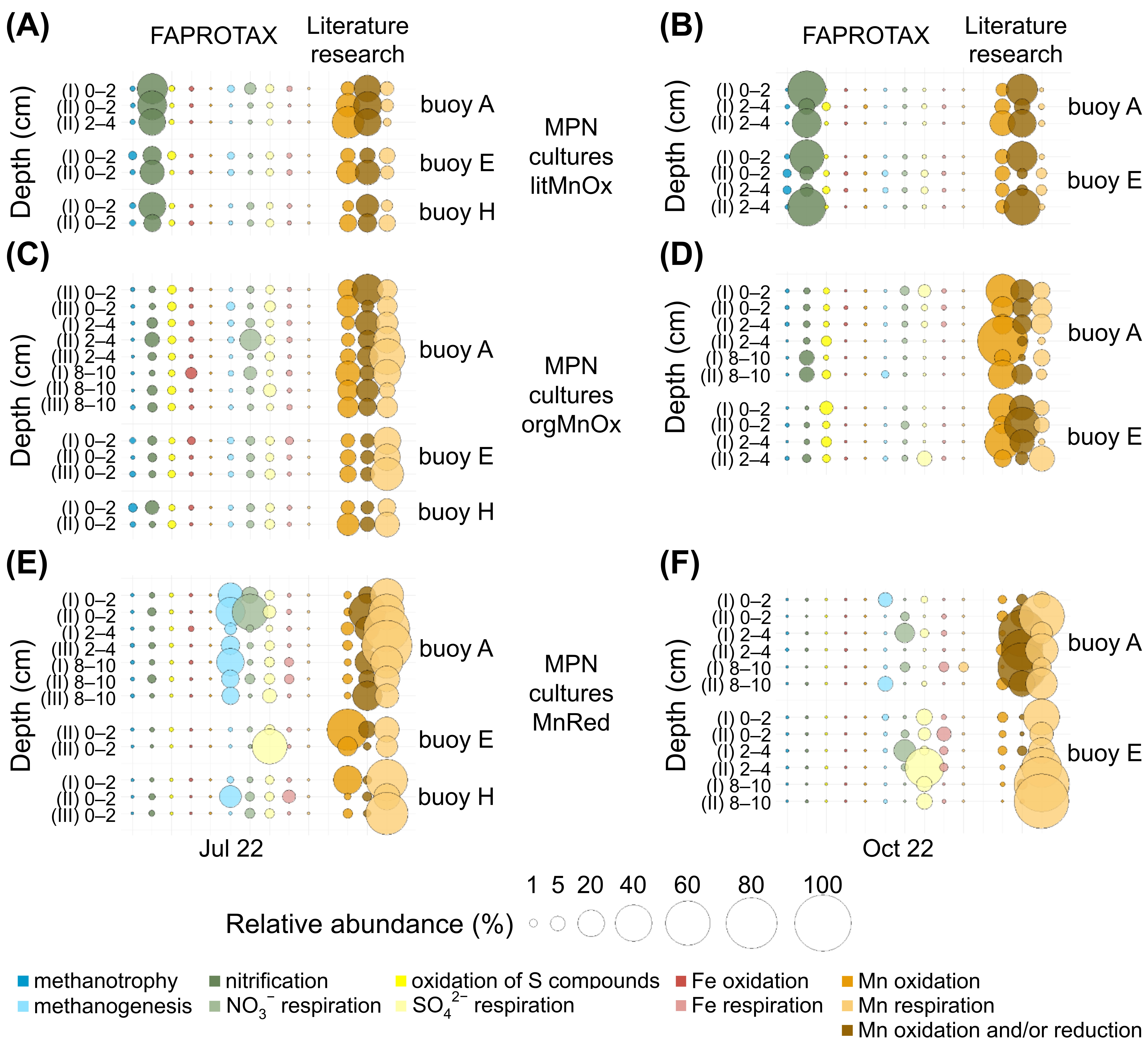


**Supplementary Figure** **S7.** Bubble plots for relative abundance (%) of 16S rRNA gene sequences for selected metabolic pathways in MPN cultures of Mn-transforming microorganisms. Comparison across three sampling locations. Panels show selected processes in **(A, B)** litMnOx, **(C, D)** orgMnOx, and **(E, F)** MnRed. MPN series were inoculated with different sediment layers (cm). Roman numerals label different dilutions of the MPN series (I, II, III). MPN series were performed with sediments from buoys A, E, and H in July and from buoys A and E in October. Functional assignments are based on FAPROTAX and own literature research.

## Supplementary Tables

Supplementary Table S1. Most abundant genera in sediment core samples for each metabolism, with functional assignments based on FAPROTAX. Listed genera represent ≥1% relative abundance among all genera associated with a specific metabolism in all sediment core samples from July, September, and October. Relative abundance (%) of each genus for a particular metabolism is shown.

| **Metabolism (FAPROTAX)** | **Genera with ≥1% relative abundance of a specific metabolism in all sediment core samples** | **Relative abundance (%) of genera for a** **particular metabolism in all sediment core samples** |
| --- | --- | --- |
| Methanotrophy | ʻ*Candidatus* Methylomirabilisʼ | 32.4 |
|  | *Crenothrix* | 24.3 |
|  | *Methylobacter* | 18.2 |
|  | *Methylocystis* | 16.3 |
| Nitrification | *Ellin6067* | 44.0 |
|  | GOUTA6 | 16.3 |
|  | MND1 | 15.5 |
|  | IS-44 | 6.5 |
|  | mle1-7 | 5.2 |
|  | ʻ*Candidatus* Nitrotogaʼ | 4.7 |
|  | DSSD61 | 4.2 |
|  | *Nitrosospira* | 1.0 |
| Oxidation of S compounds | *Thiobacillus* | 87.3 |
|  | *Tumebacillus* | 8.7 |
|  | *Bosea* | 3.8 |
| Fe oxidation | *Gallionella* | 99.6 |
| Mn oxidation | *Pedomicrobium* | 99.2 |
| Methanogenesis | *Methanoregula* | 55.6 |
|  | *Methanosarcina* | 4.9 |
|  | Rice Cluster I | 4.2 |
|  | *Methanolinea* | 1.7 |
|  | *Methanobacterium* | 1.6 |
|  | *Methanocella* | 1.2 |
| NO_3_^−^ respiration | *Dechloromonas* | 55.0 |
|  | *Escherichia* | 26.0 |
|  | *Azospira* | 5.0 |
|  | [*Desulfobacterium*] *catecholicum* group | 4.5 |
|  | *Rhodoplanes* | 1.6 |
|  | *Schlesneria* | 1.3 |
|  | *Vogesella* | 1.2 |
| SO_4_^2−^ respiration | Sva0081 sediment group | 33.8 |
|  | *Desulfatiglans* | 16.9 |
|  | *Desulfatirhabdium* | 13.3 |
|  | *Desulfocapsa* | 3.5 |
|  | *Syntrophobacter* | 3.4 |
|  | *Desulfobulbus* | 2.4 |
| Fe respiration | *Desulfuromonas* | 53.1 |
|  | *Geobacter* | 37.4 |
|  | *Bacillus* | 6.4 |
|  | *Anaeromyxobacter* | 1.6 |
| Mn respiration | *Geobacter* | 100.0 |

Supplementary Table S2. Most abundant genera in sediment core samples for possible Mn-transformation, with functional assignments based on own literature research. Listed genera represent ≥1% relative abundance among all genera associated with a specific metabolism in all sediment core samples from July, September, and October. Relative abundance (%) of each genus for a particular metabolism is shown.

| **Metabolism (own literature research)** | **Genera with ≥1% relative abundance of a specific metabolism in all sediment core samples** | **Relative abundance (%) of genera for a particular metabolism in all sediment core samples** |
| --- | --- | --- |
| Mn oxidation | *Nitrospira* | 15.6 |
|  | *Crenothrix* | 13.4 |
|  | *Arenimonas* | 12.8 |
|  | *Hyphomicrobium* | 11.8 |
|  | *Mycobacterium* | 9.9 |
|  | *Methylibium* | 4.7 |
|  | *Gallionella* | 3.5 |
|  | *Lysinibacillus* | 3.0 |
|  | *Sphingomonas* | 2.8 |
|  | *Terrimonas* | 2.4 |
|  | *Legionella* | 2.4 |
|  | *Streptomyces* | 2.3 |
|  | *Pedomicrobium* | 1.7 |
|  | *Klebsiella* | 1.7 |
|  | *Reyranella* | 1.4 |
|  | *Brevibacillus* | 1.2 |
|  | *Allorhizobium-Neorhizobium-Pararhizobium-Rhizobium* | 1.1 |
|  | *Arthrobacter* | 1.1 |
| Mn oxidation and/or reduction | *Bacillus* | 60.1 |
|  | *Ellin6067* | 33.2 |
|  | *Thiobacillus* | 4.7 |
|  | *Escherichia-Shigella* | 1.4 |
| Mn reduction | *Clostridium* | 27.5 |
|  | *Anaeromyxobacter* | 20.7 |
|  | *Paenibacillus* | 14.3 |
|  | ʻ*Candidatus* Methanoperedensʼ | 10.1 |
|  | *Desulfosporosinus* | 4.7 |
|  | *Rhodoferax* | 4.2 |
|  | *Desulfomonile* | 3.9 |
|  | *Methanosarcina* | 3.1 |
|  | *Thermincola* | 1.9 |
|  | *Thermoanaerobaculum* | 1.9 |
|  | *Desulfuromonas* | 1.7 |
|  | *Geothrix* | 1.4 |
|  | *Geobacter* | 1.2 |

**Supplementary Table S3.** MPN cultures in July and October. Cultures indicate the growth medium, sampling location, and sediment depth (cm). Samples selected for sequencing are listed. Submitted samples not sequenced further are marked in gray.

| **Growth medium** | **Location (buoy)** | **Depth (cm)** | **Sequencing samples – MPN dilution** | **Location (buoy)** | **Depth (cm)** | **Sequencing samples – MPN dilution** |
| --- | --- | --- | --- | --- | --- | --- |
|  | **MPN cultures in July** | | | **MPN cultures in October** | | |
| litMnOx | A | 0–2 | (I) 10^−1^ | A | 0–2 | (I) 10^−1^ |
|  |  |  | (II) 10^−2^ |  |  | (II) 10^−4^ |
|  |  | 2–4 | (I) 10^−1^ |  | 2–4 | (I) 10^−1^ |
|  |  |  | (II) 10^−2^ |  |  | (II) 10^−2^ |
|  | E | 0–2 | (I) 10^−1^ | E | 0–2 | (I) 10^−2^ |
|  |  |  | (II) 10^−2^ |  |  | (II) 10^−1^ |
|  | H | 0–2 | (I) 10^−1^ |  | 2–4 | (I) 10^−1^ |
|  |  |  | (II)10^−2^ |  |  | (II) 10^−2^ |
| orgMnOx | A | 0–2 | (I) 10^−1^ | A | 0–2 | (I) 10^−2^ |
|  |  |  | (II) 10^−2^ |  |  | (II) 10^−6^ |
|  |  |  | (III) 10^−2^ |  |  |  |
|  |  | 2–4 | (I) 10^−1^ |  | 2–4 | (I) 10^−1^ |
|  |  |  | (II) 10^−2^ |  |  | (II) 10^−5^ |
|  |  |  | (III) 10^−2^ |  |  |  |
|  |  | 8–10 | (I) 10^−1^ |  | 8–10 | (I) 10^−3^ |
|  |  |  | (II) 10^−2^ |  |  | (II) 10^−2^ |
|  |  |  | (III) 10^−2^ |  |  |  |
|  | E | 0–2 | (I) 10^−1^ | E |  |  |
|  |  |  | (II) 10^−2^ |  | 0–2 | (I) 10^−3^ |
|  |  |  | (III) 10^−2^ |  |  | (II) 10^−2^ |
|  | H | 0–2 | (I) 10^−1^ |  |  |  |
|  |  |  | (II) 10^−2^ |  | 2–4 | (I) 10^−3^ |
|  |  |  | (III) 10^−2^ |  |  | (II) 10^−2^ |
| MnRed | A | 0–2 | (I) 10^−4^ |  |  |  |
|  |  |  | (II) 10^−4^ | A | 0–2 | (I) 10^−4^ |
|  |  |  | (III) 10^−4^ |  |  | (II) 10^−4^ |
|  |  | 2–4 | (I) 10^−4^ |  | 2–4 | (I) 10^−3^ |
|  |  |  | (II)10^−4^ |  |  | (II)10^−4^ |
|  |  |  | (III) 10^−4^ |  | 8–10 | (I) 10^−3^  (II) 10^−3^ |
|  |  | 8–10 | (I) 10^−3^ |  |  |  |
|  |  |  | (II) 10^−3^ |  |  |  |
|  |  |  | (III) 10^−3^ | E | 0–2 | (I) 10^−4^ |
|  | E | 0–2 | (I) 10^−4^ |  |  | (II) 10^−4^ |
|  |  |  | (II) 10^−5^ |  | 2–4 | (I) 10^−4^ |
|  |  |  | (III) 10^−5^ |  |  | (II) 10^−4^ |
|  | H | 0–2 | (I) 10^−6^ |  | 8–10 | (I) 10^−4^ |
|  |  |  | (II) 10^−5^ |  |  | (II) 10^−4^ |
|  |  |  | (III) 10^−5^ |  |  |  |

**Supplementary Table S4.** Quantification of Mn-transforming microorganisms in MPN cultures from July and October. The EPA MPN Calculator data are presented in cells mL^−1^. Approaches are defined by growth medium, sampling location (buoy), and sediment depth (cm).

| Approach | 1. MPN | 2. MPN corr. | 3. L95 (C&F) | 4. U95 (C&F) | 5. L95 (L&H) | 6. U95 (L&H) | 7. S-K E | 8. Th | 9. R MPN |
| --- | --- | --- | --- | --- | --- | --- | --- | --- | --- |
|  |  | | | | | | | | |
|  | Unit: cells mL^−1^ | | | | | | | | |
|  | **MPN July** | | | | | | | | |
|  |  | | | | | | | | |
| litMnOx | | | | | | | | | |
| A, 0–2 | < 100 |  |  |  |  |  |  |  |  |
| A, 2–4 | < 100 |  |  |  |  |  |  |  |  |
| E, 0–2 | < 100 |  |  |  |  |  |  |  |  |
| H, 0–2 | < 100 |  |  |  |  |  |  |  |  |
|  | | | | | | | | | |
| orgMnOx | | | | | | | | | |
| A, 0–2 | < 100 |  |  |  |  |  |  |  |  |
| A, 2–4 | 36 | 30 | 5 | 123 | 2 | 258 | 68 | 41 | 36 |
| A, 8–10 | 36 | 30 | 5 | 123 | 2 | 258 | 68 | 41 | 36 |
| E, 0–2 | 36 | 30 | 5 | 123 | 2 | 258 | 68 | 41 | 36 |
| H, 0–2 | 93 | 74 | 5 | 271 | 9 | 418 | 147 | 116 | 91 |
|  | | | | | | | | | |
| MnRed | | | | | | | | | |
| A, 0–2 | 9,328 | 7,133 | 2,066 | 27,088 | 1,113 | 41,750 | 14,678 | 11,547 | 9,119 |
| A, 2–4 | 23,979 | 18,336 | 4,756 | 96,519 | 2,474 | 136,130 | 31,623 |  |  |
| A, 8–10 | 2,398 | 1,834 | 476 | 9,652 | 247 | 13,613 | 3,162 |  |  |
| E, 0–2 | 23,979 | 18,336 | 4,756 | 96,519 | 2,474 | 136,130 | 31,623 |  |  |
| H, 0–2 | 42,730 | 32,674 | 10,337 | 138,483 | 5,928 | 255,412 | 68,129 | 40,825 | 42,189 |
|  |  |  |  |  |  |  |  |  |  |
|  |  |  |  |  |  |  |  |  |  |
|  | **MPN October** | | | | | | | | |
|  |  | | | | | | | | |
| litMnOx | | | | | | | | | |
| A, 0–2 | 103 | 80 | 19 | 339 | 15 | 411 |  |  |  |
| A, 2–4 | < 100 |  |  |  |  |  |  |  |  |
| E, 0–2 | < 100 |  |  |  |  |  |  |  |  |
| E, 2–4 | < 100 |  |  |  |  |  |  |  |  |
|  |  |  |  |  |  |  |  |  |  |
| orgMnOx | | | | | | | | | |
| A, 0–2 | 56,477 | 43,185 | 14,844 | 158,731 | 9,174 | 205,130 |  | 58,114 | 56,704 |
| A, 2–4 | 2,145 | 1,640 | 593 | 6,016 | 392 | 6,732 |  | 2,270 | 2,146 |
| A, 8–10 | 3,114 | 2,381 | 585 | 10,255 | 435 | 10,391 |  |  |  |
| E, 0–2 | 3,114 | 2,381 | 585 | 10,255 | 435 | 10,391 |  |  |  |
| E, 2–4 | 12,352 | 9,445 | 3,361 | 36,240 | 2,379 | 34,932 |  | 12,485 | 12,413 |
|  |  |  |  |  |  |  |  |  |  |
| MnRed | | | | | | | | | |
| A, 0–2 | 1,466 | 1,121 | 408 | 4,044 | 217 | 6,342 |  | 1,921 | 1,396 |
| A, 2–4 | 852 | 651 | 213 | 2,531 | 116 | 4,171 |  | 679 | 829 |
| A, 8–10 | 566 | 433 | 106 | 1,865 | 78 | 2,457 |  |  |  |
| E, 0–2 | 3,114 | 2,381 | 585 | 10,255 | 431 | 13,457 |  |  |  |
| E, 2–4 | 3,114 | 2,381 | 585 | 10,255 | 431 | 13,457 |  |  |  |
| E, 8–10 | 3,114 | 2,381 | 585 | 10,255 | 431 | 13,457 |  |  |  |
|  | | | | | | | | | |
|  | | | | | | | | | |
| Abbreviations  1. MPN – Most Probable Number (MPN) in 1 mL sediment sample  2. MPN corr. – MPN Corrected For Bias (Thomas)  3. L97 (C&F) – Lower 95% Confidence Limit (Cornish & Fisher)  4. U95 (C&F) – Upper 95% Confidence Limit (Cornish & Fisher)  5. L95 (L&H) – Lower 95% Confidence Limit (Loyer & Hamilton)  6. U95 (L&H) – Upper 95% Confidence Limit (Loyer & Hamilton)  7. S-K E – Spearman-Karber Estimate  8. Th – Thomas  9. R MPN – R Based MPN | | | | | | | | | |
|  | | | | | | | | | |

**Supplementary Table S5.** The 20 most abundant genera in MPN cultures in all samples of a batch in one medium in July and October. Average relative abundance (%) is presented in brackets. Genera that are among the 20 most common genera only in July or October are marked in gray.

| **Growth medium** | **Top 20 genera in all samples of a batch in one medium with average relative abundance (%)** | | | | |
| --- | --- | --- | --- | --- | --- |
|  | **MPN cultures in July** | |  | **MPN cultures in October** | |
| litMnOx | *Ellin6067* (13.2) | *Pseudolabrys* (1.1) |  | *Ellin6067* (17.6) | Subgroup 10 (0.5) |
|  | *Caulobacter* (4.1) | GOUTA6 (1.0) |  | *Labrys* (5.8) | *Silvanigrella* (0.4) |
|  | *Labrys* (3.5) | *Clostridium sensu stricto* (1.0) |  | MND1 (3.9) | Unidentified Gemmatimonadaceae (0.4) |
|  | MND1 (2.9) | *Silvanigrella* (0.8) |  | *Caulobacter* (2.5) | *Anaeromyxobacter* (0.4) |
|  | *Arenimonas* (2.9) | *Reyranella* (0.7) |  | *Gaiella* (1.8) | *Hyphomicrobium* (0.4) |
|  | *Sediminibacterium* (1.8) | *Curvibacter* (0.6) |  | *Pseudolabrys* (1.0) | *Thiobacillus* (0.3) |
|  | Unidentified WWE3 (1.8) | *Rhodoferax* (0.6) |  | GOUTA6 (0.9) | Unidentified WWE3 (0.3) |
|  | *Aquicella* (1.4) | *Desulfobacca* (0.6) |  | *Aquicella* (0.9) | *Brevundimonas* (0.3) |
|  | *Bacillus* (1.1) | *Trichococcus* (0.6) |  | *Arenimonas* (0.9) | *Mycobacterium* (0.3) |
|  | *Brevundimonas* (1.1) | ʻ*Candidatus* Berkiellaʼ (0.6) |  | *Nitrospira* (0.7) | *Reyranella* (0.3) |
| orgMnOx | *Trichococcus* (8.1) | *Bdellovibrio* (1.6) |  | *Bacillus* (6.3) | *Ensifer* (1.5) |
|  | *Clostridium sensu stricto* (5.7) | *Thermomonas* (1.5) |  | *Arenimonas* (5.9) | *Sporacetigenium* (1.5) |
|  | *Bacillus* (5.1) | *Dechlorosoma* (1.0) |  | *Pseudomonas* (5.6) | *Comamonas* (1.4) |
|  | *Lentimicrobium* (5.1) | *Aeromonas* (1.0) |  | *Thermomonas* (4.3) | *Vulgatibacter* (1.2) |
|  | *Labrys* (3.6) | *Anaeromyxobacter* (0.9) |  | *Proteiniclasticum* (3.7) | *Flavobacterium* (1.2) |
|  | *Pseudomonas* (2.2) | *Fonticella* (0.9) |  | *Labrys* (3.5) | *Bosea* (1.2) |
|  | *Allorhizobium-Neorhizobium-Pararhizobium-Rhizobium* (2.2) | Unidentified Rikenellaceae (0.7) |  | *Clostridium sensu stricto* (3.3) | *Nitrosospira* (1.1) |
|  | *Sporacetigenium* (2.1) | *Ferritrophicum* (0.7) |  | *Massilia* (2.2) | *Anaeromyxobacter* (1.0) |
|  | *Proteiniclasticum* (2.1) | *Rhodoferax* (0.7) |  | *Ralstonia* (1.8) | *Aeromonas* (0.9) |
|  | *Sulfuricella* (1.8) | Christensenellaceae R-7 group (0.7) |  | *Azospirillum* (1.7) | WCHB1-32 (0.9) |
| MnRed | *Rhodoferax* (12.3) | *Desulfobulbus* (1.1) |  | *Rhodoferax* (18.0) | *Geothrix* (1.2) |
|  | *Methanosarcina* (8.2) | *Gracilibacter* (1.0) |  | *Bacillus* (6.1) | *Desulfobulbus* (1.0) |
|  | *Aeromonas* (7.1) | Christensenellaceae R-7 group (1.0) |  | *Desulfomicrobium* (4.6) | Unidentified Rikenellaceae (1.0) |
|  | *Anaeromyxobacter* (6.5) | *Anaerovorax* (0.8) |  | *Desulfosporosinus* (4.4) | *Erysipelothrix* (0.9) |
|  | *Bacillus* (6.1) | *Desulfitobacterium* (0.7) |  | *Thermincola* (3.3) | *Lentimicrobium* (0.9) |
|  | *Geothrix* (4.8) | *Clostridium sensu stricto* (0.6) |  | *Pseudomonas* (3.1) | *Methanosarcina* (0.9) |
|  | *Azospira* (3.9) | *Polaromonas* (0.4) |  | *Azoarcus* (1.7) | WCHB1-32 (0.8) |
|  | WCHB1-32 (2.2) | Unidentified Rikenellaceae (0.4) |  | ʻ*Candidatus* Accumulibacterʼ (1.6) | *Fonticella* (0.7) |
|  | *Lentimicrobium* (2.1) | ADurb.Bin063-1 (0.4) |  | *Methylotenera* (1.5) | *Aeromonas* (0.6) |
|  | *Erysipelothrix* (1.4) | *Geobacter* (0.4) |  | *Desulfitobacterium* (1.4) | *Geotalea* (0.6) |

**Supplementary Table S6.** Most abundant genera in MPN cultures for each metabolism, with functional assignments based on FAPROTAX. Listed genera represent ≥1% relative abundance among all genera associated with a specific metabolism in all MPN cultures from July and October. Relative abundance (%) of each genus for a particular metabolism is shown.

| **Metabolism (FAPROTAX)** | **Genera with ≥1% relative abundance of a specific metabolism in all MPN cultures** | **Relative abundance (%) of genera for a particular metabolism in all MPN cultures** |
| --- | --- | --- |
| Methanotrophy | *Methylocystis* | 41.3 |
|  | *Crenothrix* | 18.8 |
|  | ʻ*Candidatus* Methylomirabilisʼ | 15.4 |
|  | *Methylobacter* | 15.3 |
| Nitrification | *Ellin6067* | 72.6 |
|  | MND1 | 14.7 |
|  | *Nitrosospira* | 5.4 |
|  | GOUTA6 | 5.3 |
|  | *Nitrosomonas* | 1.5 |
|  | ʻ*Candidatus* Nitrotogaʼ | 1.0 |
| Oxidation of S compounds | *Bosea* | 66.1 |
|  | *Thiobacillus* | 33.1 |
| Fe oxidation | *Azospira* | 85.6 |
|  | *Gallionella* | 14.3 |
| Mn oxidation | *Pedomicrobium* | 86.2 |
|  | *Leptothrix* | 13.8 |
| Methanogenesis | *Methanosarcina* | 93.4 |
|  | *Methanoregula* | 2.3 |
|  | *Methanobacterium* | 1.4 |
| NO_3_^−^ respiration | *Azospira* | 53.9 |
|  | *Azoarcus* | 17.3 |
|  | *Dechlorosoma* | 13.0 |
|  | *Dechloromonas* | 6.6 |
|  | *Desulfitobacterium* | 1.6 |
|  | *Zoogloea* | 1.3 |
|  | *Lactiplantibacillus* | 1.3 |
| SO_4_^2−^ respiration | *Desulfomicrobium* | 37.5 |
|  | *Desulfobulbus* | 19.5 |
|  | *Desulfovibrio* | 4.8 |
|  | *Desulfosporosinus* | 2.5 |
|  | Sva0081 sediment group | 2.3 |
| Fe respiration | *Geobacter* | 64.5 |
|  | *Desulfosporosinus* | 18.8 |
|  | *Desulfitobacterium* | 8.7 |
|  | *Ferribacterium* | 5.2 |
|  | *Desulfuromonas* | 2.7 |
| Mn respiration | *Desulfitobacterium* | 100.0 |

**Supplementary Table S7.** Most abundant genera in MPN cultures for possible Mn-transformation, with functional assignments based on own literature research. Listed genera represent ≥1% relative abundance among all genera associated with a specific metabolism in all MPN cultures from July and October. Relative abundance (%) of each genus for a particular metabolism is shown.

| **Metabolism (own literature research)** | **Genera with ≥1% relative abundance for a specific metabolism in all MPN cultures** | **Relative abundance (%) of genera for a particular metabolism in all MPN cultures** |
| --- | --- | --- |
| Mn oxidation | *Aeromonas* | 19.3 |
|  | *Arenimonas* | 14.5 |
|  | *Thermomonas* | 13.2 |
|  | *Caulobacter* | 8.9 |
|  | *Allorhizobium-Neorhizobium-Pararhizobium-Rhizobium* | 7.0 |
|  | *Ralstonia* | 3.7 |
|  | *Bosea* | 3.4 |
|  | *Hyphomicrobium* | 3.2 |
|  | *Brevundimonas* | 3.0 |
|  | *Ensifer* | 2.8 |
|  | *Azospirillum* | 2.6 |
|  | *Flavobacterium* | 2.5 |
|  | *Klebsiella* | 2.2 |
|  | *Reyranella* | 2.0 |
|  | *Legionella* | 1.5 |
|  | *Nitrospira* | 1.3 |
|  | *Herbaspirillum* | 1.0 |
| Mn oxidation and/or reduction | *Bacillus* | 46.2 |
|  | *Ellin6067* | 27.6 |
|  | *Pseudomonas* | 16.8 |
|  | *Azospira* | 7.6 |
|  | *Thiobacillus* | 1.3 |
| Mn reduction | *Rhodoferax* | 32.6 |
|  | *Clostridium* | 10.5 |
|  | *Trichococcus* | 10.0 |
|  | *Methanosarcina* | 9.5 |
|  | *Anaeromyxobacter* | 8.9 |
|  | *Geothrix* | 5.5 |
|  | *Desulfomicrobium* | 5.0 |
|  | *Desulfosporosinus* | 3.9 |
|  | *Thermincola* | 3.9 |
|  | *Desulfobulbus* | 2.6 |
|  | *Desulfitobacterium* | 2.1 |
|  | *Azoarcus* | 1.6 |
|  | *Geobacter* | 1.1 |

**Supplementary Table S8.** Overview of known genera involved in Mn metabolism. Each genus is listed with its primary Mn metabolic function and corresponding literature reference. Genera highlighted in bold are frequently reported in the context of Mn transformation. Bolded metabolic functions indicate the primary Mn metabolism described in the literature and used for functional classification.

| Genus | Suggested Mn-metabolism | References |
| --- | --- | --- |
|  |  |  |
| *Achromobacter* | Oxidation | Carmichael and Bräuer, 2015; Mao et al., 2022; Yin et al., 2019 |
| *Acidithiobacillus* | Oxidation and/or reduction | D. B. Johnson and Pakostova, 2021; van Nguyen et al., 2018 |
| *Acidovorax* | Oxidation | Piazza et al., 2019 |
| *Acinetobacter* | **Oxidation** (and/or reduction) | Cai et al., 2023; Ehrlich and Newman, 2008; Gounot, 1994; L.-M. Zhang et al., 2008 |
| *Aeromonas* | **Oxidation** | Carmichael and Bräuer, 2015; Ehrlich and Newman, 2008; Martin-Carnahan and Joseph, 2015; Shoiful et al., 2020; Y. Zhang et al., 2019 |
| *Afipia* | Oxidation | Cai et al., 2023; Carmichael and Bräuer, 2015; Marcus et al., 2017 |
| *Agrobacterium* | Oxidation | Carmichael and Bräuer, 2015 |
| *Agromyces* | Oxidation | Cai et al., 2023; Carmichael and Bräuer, 2015; Marcus et al., 2017; W. Yang et al., 2013 |
| *Albidiferax* | **Reduction** (and/or oxidation) | Akob et al., 2014; Carmichael and Bräuer, 2015; Flynn et al., 2021; Rosenberg et al., 2014a |
| *Alcanivorax* | Oxidation | Cai et al., 2023; Sujith et al., 2014 |
| *Alteromonas* | Oxidation and/or reduction | Carmichael and Bräuer, 2015; Gounot, 1994; Lovley et al., 1989; Templeton et al., 2005 |
| *Anaeromyxobacter* | **Reduction**  (and/or oxidation) | R. Chen et al., 2022; Wenbo Liu et al., 2020; Y. Liu, Song et al., 2023; Y. Liu, Wang et al., 2023 |
| *Archaeoglobus* | Reduction | Liebensteiner et al., 2014; Lovley, 2000 |
| *Arcobacter* | Reduction | Berg et al., 2013; Vandieken et al., 2012 |
| *Arenimonas* | Oxidation | J. Liu et al., 2018; Wen Liu et al., 2018 |
| *Arthrobacter* | **Oxidation**  (and/or reduction) | Akob et al., 2014; Cai et al., 2023; Carmichael and Bräuer, 2015; Ehrlich and Newman, 2008; Gounot, 1994; Marcus et al., 2017; Tebo et al., 2005 |
| *Aurantimonas* | Oxidation | Anderson et al., 2009; Cai et al., 2023; Carmichael and Bräuer, 2015; Dick et al., 2008; Sujith and Bharathi, 2011 |
| *Azospira* | Oxidation and/or reduction | Jia et al., 2022; Mao et al., 2023; Peng et al., 2016; Tong et al., 2023 |
| *Azospirillum* | Oxidation | Dangeti et al., 2020; Mao et al., 2023 |
| *Bacillus* | Oxidation and/or reduction | Cai et al., 2023; Carmichael and Bräuer, 2015; de Vos et al., 2009; Ehrlich and Newman, 2008; C. A. Francis et al., 2002; C. A. Francis and Tebo, 2002; Gounot, 1994; Kanso et al., 2002; Kurt, 2019; Y. Liu, Wang et al., 2023; Marcus et al., 2017; J. Su et al., 2013; Sylvan et al., 2015; Tebo et al., 2004; Timofeeva et al., 2024; van Waasbergen et al., 1996; van Waasbergen et al., 1993; Zakharova et al., 2010 |
| *Bosea* | Oxidation | Marcus et al., 2017; Mariner et al., 2008; L.-M. Zhang et al., 2008 |
| *Brevibacillus* | Oxidation | Cai et al., 2023; Zeng et al., 2018; Zhao et al., 2018 |
| *Brevibacterium* | Oxidation | Cai et al., 2023; Sujith et al., 2014 |
| *Brevundimonas* | Oxidation | Cai et al., 2023; D. Hou et al., 2020; H. Zhou et al., 2016 |
| *Burkholderia* | Oxidation | Cahyani et al., 2009; Cai et al., 2023; Carmichael and Bräuer, 2015; Si et al., 2017 |
| *Caldimonas* | Oxidation | Carmichael and Bräuer, 2015; Takeda et al., 2002; Tebo et al., 2005 |
| ʻ*Candidatus* Accumulibacterʼ | Other Association | B. Ren et al., 2019; Valdivia Santibáñez, 2009 |
| ʻ*Candidatus*  Magnetobacteriumʼ | Other Association | Douterelo et al., 2019; Faivre and Schüler, 2008; Lin et al., 2014 |
| ʻ*Candidatus*  Methanoperedensʼ | Reduction | Leu et al., 2020; Wenbo Liu et al., 2020; G. Su et al., 2020 |
| *Carnobacterium* | Reduction | Bratina et al., 1998; Hammes and Hertel, 2006; Wurzel, 2000 |
| *Caulobacter* | Oxidation | Gounot, 1994; Kurt, 2019 |
|  |  |  |

*(Continued)*

**Supplementary Table S8.** Continued.

| Genus | Suggested Mn-Metabolism | References |
| --- | --- | --- |
|  |  |  |
| *Cellulomonas* | Oxidation  and/or reduction | Carmichael and Bräuer, 2015; Sani et al., 2002; Xiong et al., 2023; W. Yang et al., 2013 |
| *Chromobacterium* | Oxidation | Bromfield, 1956; Carmichael and Bräuer, 2015; Gounot, 1994; Kurt, 2019 |
| *Chryseobacterium* | Oxidation | Grandbois et al., 2017; Piazza et al., 2019; Therdkiattikul et al., 2020 |
| *Citreicella* | Oxidation | Cai et al., 2023; Rajasabapathy et al., 2015 |
| *Citrifermentans* | Reduction | Meng et al., 2022; Shrestha et al., 2022; L. Zhou et al.,2024 |
| *Citrobacter* | Oxidation | Carmichael and Bräuer, 2015; Ehrlich and Newman, 2008; Tang, Wu, et al., 2014; Tang, Xia, et al., 2014 |
| *Cladothrix* | Oxidation | Pringsheim, 1949; Sujith and Bharathi, 2011; Zapffe, 1933 |
| *Clonothrix* | Oxidation | Sujith and Bharathi, 2011; Vigliotta et al., 2007 |
| *Clostridium* | Reduction | A. J. Francis and Dodge, 1991; Lovley, 1991; Shoiful et al., 2020; Tebo et al., 2005 |
| *Corynebacterium* | Oxidation and/or reduction | Carmichael and Bräuer, 2015; Gounot, 1994 |
| *Crenothrix* | Oxidation | Cai et al., 2023; Cheng et al., 2017; Sujith and Bharathi, 2011; Vigliotta et al., 2007 |
| *Cupriavidus* | Oxidation | Bai et al., 2021; Carmichael and Bräuer, 2015; Piazza et al., 2019 |
| *Cytophaga* | Oxidation | Carmichael and Bräuer, 2015; Kurt, 2019 |
| *Deferribacter* | Reduction | Greene et al., 1997; Rosenberg et al., 2014c; Slobodkina et al., 2009 |
| *Desulfitobacterium* | Reduction | De Vos et al., 2009; Kim et al., 2012; Luijten et al., 2004; Niggemyer et al., 2001; Villemur et al., 2006 |
| *Desulfobacterium* | Reduction | Avendaño et al., 2024; Lovley and Phillips, 1994 |
| *Desulfobulbus* | Reduction | Lovley and Phillips, 1994 |
| *Desulfomicrobium* | Reduction | Lovley and Phillips, 1994; Sharak Genthner and Devereux, 2015; Thevenieau et al., 2007; |
| *Desulfosporosinus* | Reduction | Senko et al., 2009; Vandieken et al., 2017 |
| *Desulfotomaculum* | Reduction | Sass and Cypionka, 2004; Senko et al., 2009; Visser et al., 2016 |
| *Desulfovibrio* | Reduction | Gounot, 1994; Lovley and Phillips, 1994; Sass and Cypionka, 2004; Senko et al., 2009; Shoiful et al., 2020 |
| *Desulfuromonas* | Reduction | An and Picardal, 2015; Vandieken et al., 2006; Wunder et al., 2024 |
| *Desulfuromusa* | Reduction | Vandieken et al., 2006; Wunder et al., 2024 |
| *Duganella* | Oxidation | Akob et al., 2014; Carmichael and Bräuer, 2015 |
| *Ellin6067* | Oxidation and/or reduction | Jiang et al., 2024; Y. Liu, Wang et al., 2023 |
| *Ensifer* | Oxidation | Piazza et al., 2019; Z. Xiao et al., 2022 |
| *Enterobacter* | Oxidation | Carmichael and Bräuer, 2015; H. Li et al., 2022; Thatoi et al., 2023 |
| *Erythrobacter* | Oxidation | Anderson et al., 2009; Carmichael and Bräuer, 2015; C. A. Francis et al., 2001; Tebo et al., 2004; Tebo et al., 2005 |
| *Escherichia* | Oxidation and/or reduction | Appenzeller et al., 2005; Carmichael and Bräuer, 2015; Di-Ruggiero and Gounot, 1990; Lovley, 1991; Zhiyong Wang et al., 2017 |
| *Exiguobacterium* | Oxidation and/or reduction | Cai et al., 2023; Carmichael and Bräuer, 2015; Sujith et al., 2014; M. Xiao et al., 2024 |
| *Ferrimonas* | Reduction | Nakagawa et al., 2006; Nolan et al., 2010; Vandieken et al., 2012 |
| *Ferroglobus* | **Reduction**  (and/or oxidation) | Canfield et al., 2005; Lovley, 2000; Lovley et al., 2004 |
| *Fervidicella* | Reduction | Ogg and Patel, 2010 |
| *Flavobacterium* | Oxidation | Akob et al., 2014; Cai et al., 2023; Carmichael and Bräuer, 2015; Ehrlich and Newman, 2008; D. Hou et al., 2020 |
| *Fulvimarina* | Oxidation | Anderson et al., 2009; Carmichael and Bräuer, 2015; F. Ren et al., 2014 |
| *Gallionella* | Oxidation | Cai et al., 2023; Hallbeck and Pedersen, 2015; Hoover et al., 2023; Sujith and Bharathi, 2011 |
| *Geoalkalibacter* | Reduction | Greene et al., 2009; Pierra et al., 2015; Rosenberg et al., 2014b |
| *Geobacillus* | Oxidation | Douterelo et al., 2018; Elumalai et al., 2019 |
|  |  |  |

*(Continued)*

**Supplementary Table S8.** Continued.

| Genus | Suggested Mn-Metabolism | References |
| --- | --- | --- |
|  |  |  |
| *Geobacter* | **Reduction**  (and/or oxidation) | Ehrlich and Newman, 2008; Gounot, 1994; Wenbo Liu et al., 2020; Y. Liu, Wang et al., 2023; Lovley et al., 1993; Rosenberg et al., 2014b; Straub et al., 1998 |
| *Geodermatophilus* | Oxidation | Carmichael and Bräuer, 2015; Hungate et al., 1987; Ivanova et al., 2010 |
| *Geoglobus* | Reduction | Canfield et al., 2005 |
| *Georgfuchsia* | Reduction | Dorer et al., 2016; Weelink et al., 2009 |
| *Geothrix* | **Reduction** | Coates et al., 1999; Krieg et al., 2010; Y. Liu, Wang et al., 2023; Nevin and Lovley, 2002 |
| *Haloferax* | Oxidation | Alvares et al., 2023; Naik-Samant and Furtado, 2019 |
| *Halomonas* | Oxidation | Fernandes et al., 2018; Rivadeneyra et al., 2017; Xu et al., 2024 |
| *Herbaspirillum* | Oxidation | Newsome et al., 2020; L.-M. Zhang et al., 2008 |
| *Herpetosiphon* | Oxidation | Piazza et al., 2019 |
| *Hydrogenophaga* | Oxidation | Cai et al., 2023; Dangeti et al., 2020; Marcus et al, 2017 |
| *Hyphomicrobium* | Oxidation | Cai et al., 2023; Carmichael and Bräuer, 2015; Ehrlich and Newman, 2008; Gounot, 1994; Kurt, 2019; Sujith and Bharathi, 2011 |
| *Janthinobacterium* | Oxidation | Akob et al., 2014; Cai et al., 2023; Carmichael and Bräuer, 2015 |
| *Klebsiella* | Oxidation | Carmichael and Bräuer, 2015; Y. Hou et al., 2015; Y. Li et al., 2024 |
| *Lactobacillus* | Other Association | Archibald and Duong, 1984; Archibald and Fridovich, 1981; Lovley, 1991 |
| *Lapillicoccus* | Oxidation | Carmichael and Bräuer, 2015 |
| *Legionella* | Oxidation | Huston et al., 2008; Piazza et al., 2019 |
| *Leifsonia* | Oxidation | Carmichael and Bräuer, 2015 |
| *Leptolyngbya* | Oxidation | Cai et al., 2023; J. H. Park et al., 2018 |
| *Leptospirillum* | Oxidation | Cai et al., 2023; D. B. Johnson, 2015; Northup et al., 2003 |
| *Leptothrix* | Oxidation | Brouwers et al., 2000; Cai et al., 2023; Carmichael and Bräuer, 2015; Corstjens et al., 1997; Ehrlich and Newman, 2008; Emerson et al., 1989; Emerson, 2000; Gounot, 1994; Kurt, 2019; Siering and Ghiorse, 1997a, 1997b; Tebo et al., 2004; Zakharova et al., 2010; L.-M. Zhang et al., 2008 |
| *Luteibacter* | Oxidation | Carmichael and Bräuer, 2015; Q. Jin et al., 2024 |
| *Lysinibacillus* | Oxidation | Cai et al., 2023; Carmichael and Bräuer, 2015; Cerrato et al., 2010; Marcus et al., 2017; Timofeeva et al., 2024 |
| *Magnetospirillum* | Other Association | Berny et al., 2020; Prozorov et al., 2014 |
| *Marinobacter* | Oxidation | Cai et al., 2023; Carmichael and Bräuer, 2015; Liao et al., 2013 |
| *Massilia* | Other Association | Y. Wang et al., 2020; Zhao et al., 2020 |
| *"Metallogenium"* | Oxidation | Cai et al., 2023; Ehrlich and Newman, 2008; Emerson et al., 1989; Emerson, 2000; Herschel, 1995; Klaveness, 1977; Kurt, 2019; Miyajima, 1992; Sujith and Bharathi, 2011; Zakharova et al., 2010 |
| *Methanobacterium* | Other Association | Q. Chen et al., 2023; Lovley, 2000 |
| *Methanosarcina* | Reduction | Q. Chen et al., 2023; Wenbo Liu et al., 2020; L. Yu et al., 2022 |
| *Methylarcula* | Oxidation | Anderson et al., 2009; Aoki et al., 2021; Carmichael and Bräuer, 2015 |
| *Methylibium* | Oxidation | Cai et al., 2023; Miyata et al., 2024 |
| *Methylobacterium* | Oxidation | Carmichael and Bräuer, 2015; Timofeeva et al., 2024 |
| *Microbacterium* | Oxidation | Cai et al., 2023; Carmichael and Bräuer, 2015; Lan et al., 2021; Marcus et al., 2017 |
| *Microbulbifer* | Oxidation | Carmichael and Bräuer, 2015; Templeton et al., 2005 |
| *Micrococcus* | Oxidation | Carmichael and Bräuer, 2015; Gounot, 1994; Palmer et al., 1986 |
| *Microcystis* | Oxidation | Richardson et al., 1988 |
| *Mycobacterium* | Oxidation | Cai et al., 2023; Carmichael and Bräuer, 2015; Marcus et al., 2017 |
| *Naumaniella* | Oxidation | Cai et al., 2023; Zakharova et al., 2010 |
| *Nitrospira* | Oxidation | Cai et al., 2023; Miyata et al., 2024; Palomo et al., 2016 |
| *Nocardia* | Oxidation | Carmichael and Bräuer, 2015 |
| *Noviherbaspirillum* | Oxidation | Piazza et al., 2019 |
| *Oceanisphaera* | Oxidation | S.-J. Park et al., 2006 |
| *Oceanospirillum* | Oxidation | Carmichael and Bräuer, 2015; Ehrlich and Newman, 2008; Gounot, 1994 |
| *Orenia* | Reduction | Dong, Sanford, Boyanov, Kemner, Flynn, O'Loughlin, Chang et al., 2016 |
| *Oxalicibacterium* | Oxidation | Carmichael and Bräuer, 2015 |
| *Paenibacillus* | Reduction | Ehrlich and Newman, 2008; Loyaux-Lawniczak et al., 2019 |
|  |  |  |

*(Continued)*

**Supplementary Table S8.** Continued.

| Genus | Suggested Mn-Metabolism | References |
| --- | --- | --- |
|  |  |  |
| *Pantoea* | Oxidation | Carmichael and Bräuer, 2015; Sun et al., 2021 |
| *Paracoccus* | Oxidation | Carmichael and Bräuer, 2015 |
| *Pedomicrobium* | Oxidation | Anderson et al., 2009; Cai et al., 2023; Carmichael and Bräuer, 2015; Ehrlich and Newman, 2008; Gounot, 1994; Kurt, 2019; Larsen et al., 1999; Sly et al., Sujith and Bharathi 2011; 1988; Tebo et al., 2004; Tebo et al., 2005 |
| *Planococcus* | Oxidation | Carmichael and Bräuer, 2015; Palmer et al., 1986 |
| *Priestia* | Oxidation | Farda et al., 2022; Timofeeva et al., 2024 |
| *Propionibacterium* | Oxidation | Carmichael and Bräuer, 2015; L.-M. Zhang et al., 2008 |
| *Proteiniclasticum* | Other Association | Novotnik et al, 2019; Zakaria et al., 2024 |
| *Proteus* | Oxidation | Carmichael and Bräuer, 2015 |
| *Pseudoalteromonas* | Oxidation | Cai et al., 2023; Carmichael and Bräuer, 2015; Subari et al., 2018 |
| *Pseudomonas* | Oxidation and/or reduction | Brouwers et al., 1999; Cai et al., 2023; Carmichael and Bräuer, 2015; Caspi et al., 1998; Cerrato et al., 2010; de Vrind et al., 1998; de Vrind et al., 2003; Ehrlich and Newman, 2008; Gounot, 1994; Horvath et al., 2014; Lovley, 1991; Marcus et al., 2017; Shoiful et al., 2020; Tebo et al., 2004; Timofeeva et al., 2024; Zakharova et al., 2010; L.-M. Zhang, 2008 |
| *Pseudonocardia* | Oxidation | Carmichael and Bräuer, 2015 |
| *Pyrobaculum* | Reduction | Canfield et al., 2005; Kashefi and Lovley, 2000 |
| *Ralstonia* | Oxidation | Carmichael and Bräuer, 2015; Huang et al., 2018; Yabuuchi et al., 2015; W. Yang et al., 2013 |
| *Ramlibacter* | Oxidation | Guido et al., 2024; H. Yu and Leadbetter, 2020 |
| *Reyranella* | Oxidation | Marcus et al., 2017; Q. Wang et al, 2024 |
| *Rhizobium* | Oxidation | Carmichael and Bräuer, 2015 |
| *Rhodobacter* | Oxidation | Anderson et al., 2009; Cai et al., 2023; Carmichael and Bräuer, 2015; Piazza et al., 2019 |
| *Rhodococcus* | Oxidation | Carmichael and Bräuer, 2015; Singh et al., 2013; Timofeeva et al., 2024 |
| *Rhodoferax* | **Reduction** (and/or oxidation) | Akob et al., 2014; Finneran et al., 2003; Kato and Ohkuma, 2021; Lovley et al., 2004; C. Yu et al., 2024; H. Zhang et al., 2020 |
| *Riemerella* | Oxidation | Carmichael and Bräuer, 2015; Guo et al., 2023 |
| *Roseobacter* | Oxidation | Anderson et al., 2009; Carmichael and Bräuer, 2015; Jofré et al., 2021; Sujith and Bharathi 2011 |
| *Serratia* | Oxidation | Barboza et al., 2017; Cai et al., 2023 |
| *Shewanella* | Oxidation and/or reduction | Cai et al., 2023; Carmichael and Bräuer, 2015; Drewniak et al., 2015; Ehrlich and Newman, 2008; Gao et al., 2006; Gounot, 1994; Hau and Gralnick, 2007; Leonardo et al., 1999; Lovley, 1991; Myers and Myers, 1993; Venkateswaran et al., 1998 |
| *Shinella* | Oxidation | Carmichael and Bräuer, 2015 |
| *Siderocapsa* | Oxidation | Cai et al., 2023; Gounot, 1994; Hanert, 2006; Zakharova et al., 2010 |
| *Sinorhizobium* | Oxidation | Carmichael and Bräuer, 2015; Davies and Walker, 2007 |
| *Sphaerotilus* | Oxidation | A. H. Johnson and Stokes, 1966; Kurt, 2019; Sujith and Bharathi, 2011 |
| *Sphingobacterium* | Oxidation | Cai et al., 2023; Carmichael and Bräuer, 2015; Wan et al., 2020 |
| *Sphingomonas* | Oxidation | Cai et al., 2023; C. A. Francis et al., 2001; Piazza et al., 2019 |
| *Sphingopyxis* | Oxidation | Carmichael and Bräuer, 2015; Piazza et al., 2019; Sharma et al., 2021 |
| *Staphylococcus* | Oxidation | Cai et al., 2023; Carmichael and Bräuer, 2015 |
| *Stenotrophomonas* | Oxidation | Barboza et al., 2015; Cai et al., 2023; D. Hou et al., 2020; Song et al., 2022 |
| *Streptomyces* | Oxidation | Carmichael and Bräuer, 2015; Ehrlich and Newman, 2008; Therdkiattikul et al., 2020 |
| *Sulfitobacter* | Oxidation | Anderson et al., 2009; Carmichael and Bräuer, 2015; Templeton et al., 2005 |
| *Sulfolobus* | Reduction | Lovley, 1991; Lovley, 2000 |
| *Sulfurospirillum* | Reduction | Luijten et al., 2004; MacRae et al., 2007; Stolz et al., 2015 |
| *Tepidibacillus* | Reduction | Dong, Sanford, Boyanov, Kemner, Flynn, O'Loughlin, Locke, et al., 2016; S. Li et al., 2024 |
| *Thermincola* | Reduction | Tikhonova et al., 2023; Zavarzina et al., 2007 |
| *Terrabacter* | Oxidation | Carmichael and Bräuer, 2015; Piazza et al., 2019 |
| *Terrimonas* | Oxidation | Carmichael and Bräuer, 2015; Sjöberg et al., 2018 |
|  |  |  |

*(Continued)*

**Supplementary Table S8.** Continued.

| Genus | Suggested Mn-Metabolism | References |
| --- | --- | --- |
|  |  |  |
| *Thermoanaerobaculum* | Reduction | Losey et al., 2013; Zhu et al., 2022 |
| *Thermomonas* | Oxidation | D. Hou et al., 2020; D. Li et al., 2024; Y. Yang et al., 2014 |
| *Thermovenabulum* | Reduction | Ogg et al., 2010; Zavarzina et al., 2002 |
| *Thiobacillus* | Oxidation and/or reduction | Boon et al., 1998; Kumari and Natarajan, 2002; Lovley, 1991; Sugio et al., 1988 |
| *Trichococcus* | Reduction | Z. Jin et al., 2024; Zhongzhong Wang et al., 2018 |
| *Variovorax* | Oxidation | Carmichael and Bräuer, 2015; W. Yang et al., 2013 |
| *Vibrio* | Oxidation | Carmichael and Bräuer, 2015; Gounot, 1994; F. Yang et al., 2023 |
|  |  |  |

# References

Akob, D. M., Bohu, T., Beyer, A., Schäffner, F., Händel, M., Johnson, C. A., et al. (2014). Identification of Mn(II)-oxidizing bacteria from a low-pH contaminated former uranium mine. Appl. Environ. Microbiol. 80, 5086–5097. doi: 10.1128/AEM.01296-14

Alvares, J. J., Gaonkar, S. K., Naik, C. C., Asogekar, P., and Furtado, I. J. (2023). Characterization of Mn_3_O_4_-MnO_2_ nanocomposites biosynthesized by cell lysate of *Haloferax alexandrinus* GUSF-1. J. Basic Microbiol. 63, 996–1006. doi: 10.1002/jobm.202300023

An, T. T., and Picardal, F. W. (2015). *Desulfuromonas carbonis* sp. nov., an Fe(III)-, S^0^- and Mn(IV)-reducing bacterium isolated from an active coalbed methane gas well. Int. J. Syst. Evol. Microbiol. 65, 1686–1693. doi: 10.1099/ijs.0.000159

Anderson, C. R., Dick, G. J., Chu, M.‑L., Cho, J.‑C., Davis, R. E., Bräuer, S. L., et al. (2009). *Aurantimonas manganoxydans*, sp. nov. and *Aurantimonas litoralis*, sp. nov.: Mn(II) oxidizing representatives of a globally distributed clade of alpha-*Proteobacteria* from the order *Rhizobiales*. Geomicrobiol. J. 26, 189–198. doi: 10.1080/01490450902724840

Aoki, M., Miyashita, Y., Tran, P. T., Okuno, Y., Watari, T., and Yamaguchi, T. (2021). Enrichment of marine manganese-oxidizing microorganisms using polycaprolactone as a solid organic substrate. Biotechnol. Lett. 43, 813–823. doi: 10.1007/s10529-021-03088-z

Appenzeller, B. M. R., Yañez, C., Jorand, F., and Block, J.‑C. (2005). Advantage provided by iron for *Escherichia coli* growth and cultivability in drinking water. Appl. Environ. Microbiol. 71, 5621–5623. doi: 10.1128/AEM.71.9.5621-5623.2005

Archibald, F. S., and Duong, M. N. (1984). Manganese acquisition by Lactobacillus plantarum. J. Bacteriol. 158, 1–8. doi: 10.1128/jb.158.1.1-8.1984

Archibald, F. S., and Fridovich, I. (1981). Manganese and defenses against oxygen toxicity in Lactobacillus plantarum. J. Bacteriol. 145, 442–451. doi: 10.1128/jb.145.1.442-451.1981

Avendaño, K. A., Ponce-Jahen, S. J., Valenzuela, E. I., Pajares, S., Samperio-Ramos, G., Camacho-Ibar, V. F., et al. (2024). Nitrogen loss in coastal sediments driven by anaerobic ammonium oxidation coupled to microbial reduction of Mn(IV)-oxide. Sci. Total Environ. 923:171368. doi: 10.1016/j.scitotenv.2024.171368

Bai, Y., Su, J., Wen, Q., Huang, T., Chang, Q., and Ali, A. (2021). Characterization and mechanism of Mn(II)-based mixotrophic denitrifying bacterium (*Cupriavidus* sp. Hy129) in remediation of nitrate (NO_3_^-^-N) and manganese (Mn(II)) contaminated groundwater. J. Hazard. Mater. 408:124414. doi: 10.1016/j.jhazmat.2020.124414

Barboza, N. R., Amorim, S. S., Santos, P. A., Reis, F. D., Cordeiro, M. M., Guerra-Sá, R., et al. (2015). Indirect manganese removal by *Stenotrophomonas* sp. and *Lysinibacillus* sp. isolated from Brazilian mine water. Biomed. Res. Int. 2015:925972. doi: 10.1155/2015/925972

Barboza, N. R., Morais, M. M. C. A., Queiroz, P. S., Amorim, S. S., Guerra-Sá, R., and Leão, V. A. (2017). High manganese tolerance and biooxidation ability of *Serratia marcescens* isolated from manganese mine water in Minas Gerais, Brazil. Front. Microbiol. 8:1946. doi: 10.3389/fmicb.2017.01946

Berg, C., Beckmann, S., Jost, G., Labrenz, M., and Jürgens, K. (2013). Acetate-utilizing bacteria at an oxic-anoxic interface in the Baltic Sea. FEMS Microbiol. Ecol. 85, 251–261. doi: 10.1111/1574-6941.12114

Berny, C., Le Fèvre, R., Guyot, F., Blondeau, K., Guizonne, C., Rousseau, E., et al. (2020). A method for producing highly pure magnetosomes in large quantity for medical applications using *Magnetospirillum gryphiswaldense* MSR-1 magnetotactic bacteria amplified in minimal growth media. Front. Bioeng. Biotechnol. 8:16. doi: 10.3389/fbioe.2020.00016

Boon, M., Snijder, M., Hansford, G. S., and Heijnen, J. J. (1998). The oxidation kinetics of zinc sulphide with Thiobacillus ferrooxidans. Hydrometallurgy 48, 171–186. doi: 10.1016/S0304-386X(97)00081-9

Bratina, B. J., Stevenson, B. S., Green, W. J., and Schmidt, T. M. (1998). Manganese reduction by microbes from oxic regions of the Lake Vanda (Antarctica) water column. Appl. Environ. Microbiol. 64, 3791–3797. doi: 10.1128/AEM.64.10.3791-3797.1998

Bromfield, S. M. (1956). Oxidation of manganese by soil microorganisms. Aust. J. Biol. Sci. 9:238. doi: 10.1071/BI9560238

Brouwers, G. J., Corstjens, P. L. A. M., de Vrind, J. P. M., Verkamman, A., de Kuyper, M., and de Vrind-de Jong, E. W. (2000). Stimulation of Mn^2+^ oxidation in Leptothrix discophora SS-1 by Cu^2+^ and sequence analysis of the region flanking the gene encoding putative multicopper oxidase MofA. Geomicrobiol. J. 17, 25–33. doi: 10.1080/014904500270468

Brouwers, G. J., de Vrind, J. P. M., Corstjens, P. L. A. M., Cornelis, P., Baysse, C., and de Vrind-de Jong, E. W. (1999). cumA, a gene encoding a multicopper oxidase, is involved in Mn^2+^ oxidation in Pseudomonas putida GB-1. Appl. Environ. Microbiol. 65, 1762–1768. doi: 10.1128/AEM.65.4.1762-1768.1999

Cahyani, V. R., Murase, J., Ishibashi, E., Asakawa, S., and Kimura, M. (2009). Phylogenetic positions of Mn^2+^-oxidizing bacteria and fungi isolated from Mn nodules in rice field subsoils. Biol. Fertil. Soils 45, 337–346. doi: 10.1007/s00374-008-0337-8

Cai, Y., Yang, K., Qiu, C., Bi, Y., Tian, B., and Bi, X. (2023). A review of manganese-oxidizing bacteria (MnOB): Applications, future concerns, and challenges. Int. J. Environ. Res. Public Health 20:1272. doi: 10.3390/ijerph20021272

Canfield, D. E., Kristensen, E., and Thamdrup, B. (2005). The iron and manganese cycles. Adv. Mar. Biol. 48, 269–312. doi: 10.1016/S0065-2881(05)48008-6

Carmichael, S. K., and Bräuer, S. L. (2015). “Microbial diversity and manganese cycling: A review of manganese-oxidizing microbial cave communities” in Microbial Life of Cave Systems. Life in Extreme Environments, ed. A. S. Engel (Boston, MA: De Gruyter), 137–160.

Caspi, R., Tebo, B. M., and Haygood, M. G. (1998). C-type cytochromes and manganese oxidation in Pseudomonas putida MnB1. Appl. Environ. Microbiol. 64, 3549–3555. doi: 10.1128/AEM.64.10.3549-3555.1998

Cerrato, J. M., Falkinham, J. O., Dietrich, A. M., Knocke, W. R., McKinney, C. W., and Pruden, A. (2010). Manganese-oxidizing and -reducing microorganisms isolated from biofilms in chlorinated drinking water systems. Water Res. 44, 3935–3945. doi: 10.1016/j.watres.2010.04.037

Chen, Q., Wang, N., Huang, D., Yuan, T., Wu, H., and Xu, Q. (2023). Enhancement of methane production from anaerobic digestion using different manganese species. Biomass Convers. Biorefin. 13, 9783–9793. doi: 10.1007/s13399-021-01839-6

Chen, R., Liu, H., Zhang, P., Ma, J., and Jin, M. (2022). Co-response of Fe-reducing/oxidizing bacteria and Fe species to the dynamic redox cycles of natural sediment. Sci. Total Environ. 815:152953. doi: 10.1016/j.scitotenv.2022.152953

Cheng, Q., Nengzi, L., Bao, L., Huang, Y., Liu, S., Cheng, X., et al. (2017). Distribution and genetic diversity of microbial populations in the pilot-scale biofilter for simultaneous removal of ammonia, iron and manganese from real groundwater. Chemosphere 182, 450–457. doi: 10.1016/j.chemosphere.2017.05.075

Coates, J. D., Ellis, D. J., Gaw, C. V., and Lovley, D. R. (1999). Geothrix fermentans gen. nov., sp. nov., a novel Fe(III)-reducing bacterium from a hydrocarbon-contaminated aquifer. Int. J. Syst. Bacteriol. 49, 1615–1622. doi: 10.1099/00207713-49-4-1615

Corstjens, P. L. A. M., de Vrind, J. P. M., Goosen, T., and Jong, E. W. d. V. (1997). Identification and molecular analysis of the Leptothrix discophora SS‐1 *mofA* gene, a gene putatively encoding a manganese‐oxidizing protein with copper domains. Geomicrobiol. J. 14, 91–108. doi: 10.1080/01490459709378037

Dangeti, S., McBeth, J. M., Roshani, B., Vyskocil, J. M., Rindall, B., and Chang, W. (2020). Microbial communities and biogenic Mn-oxides in an on-site biofiltration system for cold Fe(II)- and Mn(II)-rich groundwater treatment. Sci. Total Environ. 710:136386. doi: 10.1016/j.scitotenv.2019.136386

Davies, B. W., and Walker, G. C. (2007). Disruption of sitA compromises Sinorhizobium meliloti for manganese uptake required for protection against oxidative stress. J. Bacteriol. 189, 2101–2109. doi: 10.1128/jb.01377-06

De Vos, P., Garrity, G. M., Jones, D., Krieg, N. R., Ludwig, W., Rainey, F. A., Schleifer, K.-H., Whitman, W. B. (2009). Bergey’s Manual of Systematic Bacteriology: The Firmicutes. New York, NY: Springer.

De Vrind, J. P. M., Brouwers, G. J., Corstjens, P. L. A. M., den Dulk, J., and de Vrind-de Jong, E. W. (1998). The cytochrome c maturation operon is involved in manganese oxidation in Pseudomonas putida GB-1. Appl. Environ. Microbiol. 64, 3556–3562. doi: 10.1128/AEM.64.10.3556-3562.1998

De Vrind, J. P. M., de Groot, A., Brouwers, G. J., Tommassen, J., and de Vrind-de Jong, E. W. (2003). Identification of a novel Gsp-related pathway required for secretion of the manganese-oxidizing factor of Pseudomonas putida strain GB-1. Mol. Microbiol. 47, 993–1006. doi: 10.1046/j.1365-2958.2003.03339.x

Dick, G. J., Podell, S., Johnson, H. A., Rivera-Espinoza, Y., Bernier-Latmani, R., McCarthy, J. K., et al. (2008). Genomic insights into Mn(II) oxidation by the marine alphaproteobacterium Aurantimonas sp. strain SI85-9A1. Appl. Environ. Microbiol. 74, 2646–2658. doi: 10.1128/AEM.01656-07

Di-Ruggiero, J., and Gounot, A. M. (1990). Microbial manganese reduction mediated by bacterial strains isolated from aquifer sediments. Microb. Ecol. 20, 53–63. doi: 10.1007/BF02543866

Dong, Y., Sanford, R. A., Boyanov, M. I., Kemner, K. M., Flynn, T. M., O'Loughlin, E. J., et al. (2016). Orenia metallireducens sp. nov. strain Z6, a novel metal-reducing member of the phylum Firmicutes from the deep subsurface. Appl. Environ. Microbiol. 82, 6440–6453. doi: 10.1128/AEM.02382-16

Dong, Y., Sanford, R. A., Boyanov, M. I., Kemner, K. M., Flynn, T. M., O'Loughlin, E. J., et al. (2016). Tepidibacillus decaturensis sp. nov., a microaerophilic, moderately thermophilic iron-reducing bacterium isolated from 1.7 km depth groundwater. Int. J. Syst. Evol. Microbiol. 66, 3964–3971. doi: 10.1099/ijsem.0.001295

Dorer, C., Vogt, C., Neu, T. R., Stryhanyuk, H., and Richnow, H.‑H. (2016). Characterization of toluene and ethylbenzene biodegradation under nitrate-, iron(III)- and manganese(IV)-reducing conditions by compound-specific isotope analysis. Environ. Pollut. 211, 271–281. doi: 10.1016/j.envpol.2015.12.029

Douterelo, I., Sharpe, R. L., Husband, S., Fish, K. E., and Boxall, J. B. (2019). Understanding microbial ecology to improve management of drinking water distribution systems. WIREs Water 6:e01325. doi: 10.1002/wat2.1325

Drewniak, L., Stasiuk, R., Uhrynowski, W., and Sklodowska, A. (2015). *Shewanella* sp. O23s as a driving agent of a system utilizing dissimilatory arsenate-reducing bacteria responsible for self-cleaning of water contaminated with arsenic. Int. J. Mol. Sci. 16, 14409–14427. doi: 10.3390/ijms160714409

Ehrlich, H. L., and Newman, D. K. (2008). Geomicrobiology. Boca Raton, FL: CRC Press.

Elumalai, P., Parthipan, P., Narenkumar, J., Anandakumar, B., Madhavan, J., Oh, B.‑T., et al. (2019). Role of thermophilic bacteria (*Bacillus* and *Geobacillus*) on crude oil degradation and biocorrosion in oil reservoir environment. 3 Biotech 9, 1604. doi: 10.1007/s13205-019-1604-0

Emerson, D. (2000). “Microbial oxidation of Fe (II) and Mn (II) at circumneutral pH”, in Environmental Microbe‐Metal Interactions, ed. Lovley, D. R. (Washington, DC: ASM Press), 31-52.

Emerson, D., Garen, R. E., and Ghiorse, W. C. (1989). Formation of *Metallogenium*-like structures by a Mn-oxidizing fungus. Arch. Microbiol. 151, 223–231. doi: 10.1007/BF00413134

Faivre, D., and Schüler, D. (2008). Magnetotactic bacteria and magnetosomes. Chem. Rev. 108, 4875–4898. doi: 10.1021/cr078258w

Farda, B., Djebaili, R., Del Gallo, M., Ercole, C., Bellatreccia, F., and Pellegrini, M. (2022). The “Infernaccio” gorges: Microbial diversity of black deposits and isolation of manganese-solubilizing bacteria. Biology 11:1204. doi: 10.3390/biology11081204

Fernandes, S. O., Surya Prakash, L., Balan Binish, M., Padinchati Krishnan, K., and John Kurian, P. (2018). Changes in morphology and metabolism enable Mn-oxidizing bacteria from mid-oceanic ridge environment to counter metal-induced stress. J. Basic Microbiol. 58, 390–402. doi: 10.1002/jobm.201700580

Finneran, K. T., Johnsen, C. V., and Lovley, D. R. (2003*Rhodoferax ferrireducens* sp. nov., a psychrotolerant, facultatively anaerobic bacterium that oxidizes acetate with the reduction of Fe (III). Int. J. Syst. Evol. Microbiol. 53, 669–673. doi: 10.1099/ijs.0.02298-0

Flynn, T. M., Antonopoulos, D. A., Skinner, K. A., Brulc, J. M., Johnston, E., Boyanov, M. I., et al. (2021). Biogeochemical dynamics and microbial community development under sulfate- and iron-reducing conditions based on electron shuttle amendment. PLoS One 16:e0251883. doi: 10.1371/journal.pone.0251883

Francis, A. J., and Dodge, C. J. (1991). Dissolution of ferrites by Clostridium sp.. Geomicrobiol. J. 9, 27–40. doi: 10.1080/01490459109385983

Francis, C. A., Casciotti, K. L., and Tebo, B. M. (2002). Localization of Mn(II)-oxidizing activity and the putative multicopper oxidase, MnxG, to the exosporium of the marine *Bacillus* sp. strain SG-1. Arch. Microbiol. 178, 450–456. doi: 10.1007/s00203-002-0472-9

Francis, C. A., Co, E. M., and Tebo, B. M. (2001). Enzymatic manganese(II) oxidation by a marine alpha-proteobacterium. Appl. Environ. Microbiol. 67, 4024–4029. doi: 10.1128/AEM.67.9.4024-4029.2001

Francis, C. A., and Tebo, B. M. (2002). Enzymatic manganese (II) oxidation by metabolically dormant spores of diverse *Bacillus* species. Appl. Environ. Microbiol. 68, 874–880. doi: 10.1128/AEM.68.2.874-880.2002

Gao, H., Obraztova, A., Stewart, N., Popa, R., Fredrickson, J. K., Tiedje, J. M., et al. (2006). *Shewanella loihica* sp. nov., isolated from iron-rich microbial mats in the Pacific Ocean. Int. J. Syst. Evol. Microbiol. 56, 1911–1916. doi: 10.1099/ijs.0.64354-0

Gounot, A. M. (1994). Microbial oxidation and reduction of manganese: Consequences in groundwater and applications. FEMS Microbiol. Rev. 14, 339–349. doi: 10.1111/j.1574-6976.1994.tb00108.x

Grandbois, R., Yeager, C., Tani, Y., Xu, C., Zhang, S., Beaver, M., et al. (2017). Biogenic manganese oxides facilitate iodide oxidation at pH ≤ 5. Geomicrobiol. J. 35, 167–173. doi: 10.1080/01490451.2017.1338795

Greene, A. C., Patel, B. K. C., and Sheehy, A. J. (1997). *Deferribacter thermophilus* gen. nov., sp. nov., a novel thermophilic manganese- and iron-reducing bacterium isolated from a petroleum reservoir. Int. J. Syst. Bacteriol. 47, 505–509. doi: 10.1099/00207713-47-2-505

Greene, A. C., Patel, B. K. C., and Yacob, S. (2009). *Geoalkalibacter subterraneus* sp. nov., an anaerobic Fe(III)- and Mn(IV)-reducing bacterium from a petroleum reservoir, and emended descriptions of the family Desulfuromonadaceae and the genus *Geoalkalibacter*. Int. J. Syst. Evol. Microbiol. 59, 781–785. doi: 10.1099/ijs.0.001537-0

Guido, A., Calcagnile, M., Talà, A., Tredici, S. M., Belmonte, G., and Alifano, P. (2024). Microbial consortium involved in ferromanganese and francolite biomineralization in an anchialine environment (Zinzulùsa Cave, Castro, Italy). Sci. Total Environ. 936:173423. doi: 10.1016/j.scitotenv.2024.173423

Guo, F., Wang, M., Huang, M., Jiang, Y., Gao, Q., Zhu, D., et al. (2023). Manganese efflux achieved by MetA and MetB affects oxidative stress resistance and iron homeostasis in *Riemerella anatipestifer*. Appl. Environ. Microbiol. 89:e0183522. doi: 10.1128/aem.01835-22

Hallbeck, L. E.‑L., and Pedersen, K. (2015). “*Gallionella*”, in Bergey’s Manual of Systematics of Archaea and Bacteria, eds. P. de Vos, J. Chun, S. Dedysh, B. Hedlund, P. Kämpfer, F. A. Rainey, et al. (Hoboken, NJ: Wiley), 1–10.

Hammes, W. P., and Hertel, C. (2006). “The genera *Lactobacillus* and *Carnobacterium*”, in The Prokaryotes, eds. M. Dworkin, S. Falkow, E. Rosenberg, K.-H. Schleifer, and E. Stackebrandt (New York, NY: Springer), 320–403.

Hanert, H. H. (2006). “The genus *Siderocapsa* (and other iron- and manganese-oxidizing Eubacteria)”, in The Prokaryotes, eds. M. Dworkin, S. Falkow, E. Rosenberg, K.-H. Schleifer, and E. Stackebrandt (New York, NY: Springer), 1005–1015.

Hau, H. H., and Gralnick, J. A. (2007). Ecology and biotechnology of the genus Shewanella. Annu. Rev. Microbiol. 61, 237–258. doi: 10.1146/annurev.micro.61.080706.093257

Herschel, A. (1995). Auflösung und Ausfällung von Mangan in stehenden Oberflächengewässern [Dissertation]. Technische Universität Dresden, Dresden.

Hoover, R. L., Keffer, J. L., Polson, S. W., and Chan, C. S. (2023). Gallionellaceae pangenomic analysis reveals insight into phylogeny, metabolic flexibility, and iron oxidation mechanisms. mSystems 8:e0003823. doi: 10.1128/msystems.00038-23

Horvath, A. S., Garrick, L. V., and Moreau, J. W. (2014). Manganese-reducing *Pseudomonas fluorescens*-group bacteria control arsenic mobility in gold mining-contaminated groundwater. Environ. Earth Sci. 71, 4187–4198. doi: 10.1007/s12665-013-2809-x

Hou, D., Zhang, P., Wei, D., Zhang, J., Yan, B., Cao, L., et al. (2020). Simultaneous removal of iron and manganese from acid mine drainage by acclimated bacteria. J. Hazard. Mater. 396:122631. doi: 10.1016/j.jhazmat.2020.122631

Hou, Y., Cheng, K., Li, Z., Ma, X., Wei, Y., Zhang, L., et al. (2015). Biosorption of cadmium and manganese using free cells of *Klebsiella* sp. isolated from waste water. PLoS One 10:e0140962. doi: 10.1371/journal.pone.0140962

Huang, H., Zhao, Y., Xu, Z., Ding, Y., Zhang, W., and Wu, L. (2018). Biosorption characteristics of a highly Mn(II)-resistant *Ralstonia pickettii* strain isolated from Mn ore. PLoS One 13:e0203285. doi: 10.1371/journal.pone.0203285

Hungate, B., Danin, A., Pellerin, N. B., Stemmler, J., Kjellander, P., Adams, J. B., et al. (1987). Characterization of manganese-oxidizing (MnII→MnIV) bacteria from Negev Desert rock varnish: implications in desert varnish formation. Can. J. Microbiol. 33, 939–943. doi: 10.1139/m87-165

Huston, W. M., Naylor, J., Cianciotto, N. P., Jennings, M. P., and McEwan, A. G. (2008). Functional analysis of the multi-copper oxidase from *Legionella pneumophila*. Microbes Infect. 10, 497–503. doi: 10.1016/j.micinf.2008.01.011

Ivanova, N., Sikorski, J., Jando, M., Munk, C., Lapidus, A., Del Glavina Rio, T., et al. (2010). Complete genome sequence of *Geodermatophilus obscurus* type strain (G-20T). Stand. Genomic Sci. 2, 158–167. doi: 10.4056/sigs.711311

Jia, L., Wu, W., Zhou, Q., Li, Y., and Wu, W. (2022). New insights on the synergetic removal of nutrients and sulfonamides in solid carbon/manganese ore supported denitrification system: Water quality, microbial community and antibiotic resistance genes. Chem. Eng. J. 446:136992. doi: 10.1016/j.cej.2022.136992

Jiang, Z., Huang, X., Wang, S., Xiong, J., Xie, C., and Chen, Y. (2024). Divalent manganese stimulates the removal of nitrate by anaerobic sludge. RSC Adv. 14, 2447–2452. doi: 10.1039/D3RA07088C

Jin, Q., Zhang, L., Cao, K., Pan, X., Liu, Z., Guan, Y., et al. (2024). Iron-nitrogen coupling mediates soil microbial community composition and gene expression during the formation of rusty root in *Panax ginseng* from three northeast provinces in China. J. Environ. Chem. Eng. 12, 114068. doi: 10.1016/j.jece.2024.114068

Jin, Z., Liang, L., Zhao, Z., and Zhang, Y. (2024). Enhancing assimilatory sulfate reduction with ferrihydrite-humic acid coprecipitate in anaerobic sulfate-containing wastewater treatment. Bioresour. Technol. 411:131308. doi: 10.1016/j.biortech.2024.131308

Jofré, I., Matus, F., Mendoza, D., Nájera, F., and Merino, C. (2021). Manganese-oxidizing Antarctic bacteria (Mn-Oxb) release reactive oxygen species (ROS) as secondary Mn(II) oxidation mechanisms to avoid toxicity. Biology 10, 1004. doi: 10.3390/biology10101004

Johnson, A. H., and Stokes, J. L. (1966). Manganese oxidation by Sphaerotilus discophorus. J. Bacteriol. 91, 1543–1547. doi: 10.1128/jb.91.4.1543-1547.1966

Johnson, D. B. (2015). “*Leptospirillum*”, in Bergey’s Manual of Systematics of Archaea and Bacteria, eds. P. de Vos, J. Chun, S. Dedysh, B. Hedlund, P. Kämpfer, F. A. Rainey, et al. (Hoboken, NJ: Wiley), 1–8.

Johnson, D. B., and Pakostova, E. (2021). Dissolution of manganese (IV) oxide mediated by acidophilic bacteria, and demonstration that manganese (IV) can act as both a direct and indirect electron acceptor for iron-reducing *Acidithiobacillus* spp.*.* Geomicrobiol. J. 38, 570–576. doi: 10.1080/01490451.2021.1903624

Kanso, S., Greene, A. C., and Patel, B. K. C. (2002). *Bacillus subterraneus* sp. nov., an iron- and manganese-reducing bacterium from a deep subsurface Australian thermal aquifer. Int. J. Syst. Evol. Microbiol. 52, 869–874. doi: 10.1099/00207713-52-3-869

Kashefi, K., and Lovley, D. R. (2000). Reduction of Fe(III), Mn(IV), and toxic metals at 100°C by *Pyrobaculum islandicum*. Appl. Environ. Microbiol. 66, 1050–1056. doi: 10.1128/AEM.66.3.1050-1056.2000

Kato, S., and Ohkuma, M. (2021). A single bacterium capable of oxidation and reduction of iron at circumneutral pH. Microbiol. Spectr. 9:e0016121. doi: 10.1128/spectrum.00161-21

Kim, S.‑H., Harzman, C., Davis, J. K., Hutcheson, R., Broderick, J. B., Marsh, T. L., et al. (2012). Genome sequence of *Desulfitobacterium hafniense* DCB-2, a Gram-positive anaerobe capable of dehalogenation and metal reduction. BMC Microbiol. 12:21. doi: 10.1186/1471-2180-12-21

Klaveness, D. (1977). Morphology, distribution and significance of the manganese-accumulating microorganism *Metallogenium* in lakes. Hydrobiologia 56, 25–33. doi: 10.1007/BF00023282

Krieg, N. R., Staley, J. T., Brown, D., Hedlund, B. P., Paster, B. J., Ward, N. L., et al. (2010). Bergey’s Manual of Systematic Bacteriology: The Bacteroidetes, Spirochaetes, Tenericutes (Mollicutes), Acidobacteria, Fibrobacteres, Fusobacteria, Dictyoglomi, Gemmatimonadetes, Lentisphaerae, Verrucomicrobia, Chlamydiae, and Planctomycetes. New York, NY: Springer.

Kumari, A., and Natarajan, K. (2002). Development of a clean bioelectrochemical process for leaching of ocean manganese nodules. Miner. Eng. 15, 103–106. doi: 10.1016/S0892-6875(01)00209-6

Kurt, H. (2019). “Depth distribution of microbial diversity in lakes”, in Freshwater microbiology: Perspectives of bacterial dynamics in lake ecosystems, eds. S. Shafi, S. A. Bandh, and N. Shameem (San Diego, CA: Academic Press), 225–262.

Lan, J., Sun, Y., Chen, X., Zhan, W., Du, Y., Zhang, T. C., et al. (2021). Bio-leaching of manganese from electrolytic manganese slag by *Microbacterium trichothecenolyticum* Y1: Mechanism and characteristics of microbial metabolites. *Bioresour. Technol.* 319:124056. doi: 10.1016/j.biortech.2020.124056

Larsen, E. I., Sly, L. I., and McEwan, A. G. (1999). Manganese(II) adsorption and oxidation by whole cells and a membrane fraction of *Pedomicrobium* sp. ACM 3067. *Arch. Microbiol.* 171, 257–264. doi: 10.1007/s002030050708

Leonardo, M. R., Moser, D. P., Barbieri, E., Brantner, C. A., MacGregor, B. J., Paster, B. J., et al. (1999). *Shewanella pealeana* sp. nov., a member of the microbial community associated with the accessory nidamental gland of the squid *Loligo pealei*. *Int. J. Syst. Bacteriol.* 49, 1341–1351. doi: 10.1099/00207713-49-4-1341

Leu, A. O., Cai, C., McIlroy, S. J., Southam, G., Orphan, V. J., Yuan, Z., et al. (2020). Anaerobic methane oxidation coupled to manganese reduction by members of the Methanoperedenaceae. *ISME J.* 14, 1030–1041. doi: 10.1038/s41396-020-0590-x

Li, D., Zhang, J., Yu, J., and Wang, H. (2024). Synergistic denitrification and Mn cycle driven by sulfur autotrophy significantly enhanced nitrogen removal. *J. Environ. Chem. Eng.* 12:112917. doi: 10.1016/j.jece.2024.112917

Li, H., Wu, Y., Tang, Y., Fang, B., Luo, P., Yang, L., et al. (2022). A manganese-oxidizing bacterium-*Enterobacter hormaechei* strain DS02Eh01: Capabilities of Mn(II) immobilization, plant growth promotion and biofilm formation. *Environ. Pollut.* 309:119775. doi: 10.1016/j.envpol.2022.119775

Li, S., Li, C., Pei, J., Liu, R., Fang, J., Wei, Y., et al. (2024). *Tepidibacillus marianensis* sp. nov., a novel heterotrophic iron-reducing bacterium isolated from Mariana Trench sediment. *Int. J. Syst. Evol. Microbiol.* 74:006438. doi: 10.1099/ijsem.0.006438

Li, Y., Liu, J., Mo, Z., and Xu, Z. (2024). Bio-oxidization of Mn(II) in acidic wastewater by *Klebsiella* sp. strain M3 and its immobilization towards concomitant Sb(III). *Biomass Convers. Biorefin.* 14, 27099–27112. doi: 10.1007/s13399-022-03376-2

Liao, S., Zhou, J., Wang, H., Chen, X., Wang, H., and Wang, G. (2013). Arsenite oxidation using biogenic manganese oxides produced by a deep-sea manganese-oxidizing bacterium, *Marinobacter* sp. MnI7-9. *Geomicrobiol. J.* 30, 150–159. doi: 10.1080/01490451.2011.654379

Liebensteiner, M. G., Stams, A., and Lomans, B. P. (2014). (Per)chlorate reduction at high temperature: Physiological study of *Archaeoglobus fulgidus* and potential implications for novel souring mitigation strategies. *Int. Biodeterior. Biodegrad.* 96, 216–222. doi: 10.1016/j.ibiod.2014.07.019

Lin, W., Deng, A., Wang, Z., Li, Y., Wen, T., Wu, L.‑F., et al. (2014). Genomic insights into the uncultured genus ‘*Candidatus* Magnetobacterium’ in the phylum Nitrospirae. *ISME J.* 8, 2463–2477. doi: 10.1038/ismej.2014.94

Liu, J., Yao, J., Wang, F., Ni, W., Liu, X., Sunahara, G., et al. (2018). China’s most typical nonferrous organic-metal facilities own specific microbial communities. *Sci. Rep.* 8:12570. doi: 10.1038/s41598-018-30519-1

Liu, W. [Wen], Jiang, H., Yang, J., and Wu, G. (2018). Gammaproteobacterial diversity and carbon utilization in response to salinity in the lakes on the Qinghai–Tibetan Plateau. *Geomicrobiol. J.* 35, 392–403. doi: 10.1080/01490451.2017.1378951

Liu, W. [Wenbo], Xiao, H., Ma, H., Li, Y., Adyel, T. M., and Zhai, J. (2020). Reduction of methane emissions from manganese-rich constructed wetlands: Role of manganese-dependent anaerobic methane oxidation. *Chem. Eng. J.* 387:123402. doi: 10.1016/j.cej.2019.123402

Liu, Y., Song, X., Wang, Y., Hou, X., Wang, Y., and Cao, X. (2023). Adaptive evolutionary strategies of microbes to manganese in nitrogen-culturing sludge. *J. Clean. Prod.* 430:139666. doi: 10.1016/j.jclepro.2023.139666

Liu, Y., Wang, Y., Song, X., Hou, X., Cao, X., and Wang, Y. (2023). The evolution of nitrogen transformation microorganism consortium under continued manganese domestication conditions. *Sci. Total Environ.* 899:165656. doi: 10.1016/j.scitotenv.2023.165656

Losey, N. A., Stevenson, B. S., Busse, H.‑J., Damsté, J. S. S., Rijpstra, W. I. C., Rudd, S., et al. (2013). *Thermoanaerobaculum aquaticum* gen. nov., sp. nov., the first cultivated member of Acidobacteria subdivision 23, isolated from a hot spring. *Int. J. Syst. Evol. Microbiol.* 63, 4149–4157. doi: 10.1099/ijs.0.051425-0

Lovley, D. R. (1991). Dissimilatory Fe(III) and Mn(IV) reduction. *Microbiol. Rev.* 55, 259–287. doi: 10.1128/mr.55.2.259-287.1991

Lovley, D. R. (2000). “Fe(III) and Mn(IV) reduction” in Environmental Microbe-Metal Interaction, ed. D. R. Lovley (Washington, DC: ASM Press), 1–30.

Lovley, D. R., Giovannoni, S. J., White, D. C., Champine, J. E., Phillips, E. J., Gorby, Y. A., et al. (1993). *Geobacter metallireducens* gen. nov. sp. nov., a microorganism capable of coupling the complete oxidation of organic compounds to the reduction of iron and other metals. *Arch. Microbiol.* 159, 336–344. doi: 10.1007/BF00290916

Lovley, D. R., Holmes, D. E., and Nevin, K. P. (2004). “Dissimilatory Fe(III) and Mn(IV) reduction”, in Advances in Microbial Physiology, eds. R. K. Pool, and D. J. Kelly (London: Academic Press), 219–286.

Lovley, D. R., and Phillips, E. J. (1994). Novel processes for anaerobic sulfate production from elemental sulfur by sulfate-reducing bacteria. *Appl. Environ. Microbiol.* 60, 2394–2399. doi: 10.1128/aem.60.7.2394-2399.1994

Lovley, D. R., Phillips, E. J., and Lonergan, D. J. (1989). Hydrogen and formate oxidation coupled to dissimilatory reduction of iron or manganese by *Alteromonas putrefaciens*. *Appl. Environ. Microbiol.* 55, 700–706. doi: 10.1128/aem.55.3.700-706.1989

Loyaux-Lawniczak, S., Vuilleumier, S., and Geoffroy, V. A. (2019). Efficient reduction of iron oxides by *Paenibacillus* spp. strains isolated from tropical soils. *Geomicrobiol. J.* 36, 423–432. doi: 10.1080/01490451.2019.1566415

Luijten, M. L. G. C., Weelink, S. A. B., Godschalk, B., Langenhoff, A. A. M., van Eekert, M. H. A., Schraa, G., et al. (2004). Anaerobic reduction and oxidation of quinone moieties and the reduction of oxidized metals by halorespiring and related organisms. *FEMS Microbiol. Ecol.* 49, 145–150. doi: 10.1016/j.femsec.2004.01.015

MacRae, J. D., Lavine, I. N., McCaffery, K. A., and Ricupero, K. (2007). Isolation and characterization of NP4, arsenate-reducing *Sulfurospirillum* from Maine groundwater. *J. Environ. Eng.* 133, 81–88. doi: 10.1061/(ASCE)0733-9372(2007)133:1(81)

Mao, Q., Luo, Z., Yan, B., Zhou, Y., Luo, S., Yang, Y., et al. (2023). Manganese removal from acid mine drainage by a consortium of Mn-oxidizing bacteria in continuous stirred tank bioreactor: Long-term treatment and reactive mixture characterization. *ACS ES&T Water* 3, 1620–1629. doi: 10.1021/acsestwater.2c00412

Mao, Q., Wei, D., Yan, B., Luo, S., Seviour, T. W., Wei, Z., et al. (2022). Removal of manganese in acidic solutions utilizing *Achromobacter* sp. strain QBM-4 isolated from mine drainage. *Process Saf. Environ. Prot.* 165, 920–928. doi: 10.1016/j.psep.2022.04.002

Marcus, D. N., Pinto, A., Anantharaman, K., Ruberg, S. A., Kramer, E. L., Raskin, L., et al. (2017). Diverse manganese(II)‐oxidizing bacteria are prevalent in drinking water systems. *Environ. Microbiol. Rep.* 9, 120–128. doi: 10.1111/1758-2229.12508

Mariner, R., Johnson, D. B., and Hallberg, K. B. (2008). Characterisation of an attenuation system for the remediation of Mn(II) contaminated waters. *Hydrometallurgy* 94, 100–104. doi: 10.1016/j.hydromet.2008.05.024

Martin‐Carnahan, A., and Joseph, S. W. (2015). “*Aeromonas*”, in Bergey’s Manual of Systematics of Archaea and Bacteria, eds. P. de Vos, J. Chun, S. Dedysh, B. Hedlund, P. Kämpfer, F. A. Rainey, et al. (Hoboken, NJ: Wiley), 1–44.

Meng, L., Xie, L., Hirose, Y., Nishiuchi, T., and Yoshida, N. (2022). Reduced graphene oxide increases cells with enlarged outer membrane of *Citrifermentans bremense* and exopolysaccharides secretion. *Biosens. Bioelectron.* 218:114754. doi: 10.1016/j.bios.2022.114754

Miyajima, T. (1992). Biological manganese oxidation in a lake I: Occurrence and distribution of *Metallogenium* sp. and its kinetic properties. *Arch. Hydrobiol.* 124, 317–335.

Miyata, N., Suganuma, R., Sunouchi, K., Okano, K., Fuchida, S., Watanabe, M., et al. (2024). Biological Mn(II) oxidation under organic substrate-limited conditions and its application in mine drainage remediation. *Biochem. Eng. J.* 203:109187. doi: 10.1016/j.bej.2023.109187

Myers, C. R., and Myers, J. M. (1993). Role of menaquinone in the reduction of fumarate, nitrate, iron(III) and manganese(IV) by *Shewanella putrefaciens* MR-1. *FEMS Microbiol. Lett.* 114, 215–222. doi: 10.1111/j.1574-6968.1993.tb06576.x

Naik-Samant, S., and Furtado, I. (2019). Formation of rhodochrosite by *Haloferax alexandrinus* GUSF-1. *J. Clust. Sci.* 30, 1435–1441. doi: 10.1007/s10876-019-01586-9

Nakagawa, T., Iino, T., Suzuki, K.-I., and Harayama, S. (2006). *Ferrimonas futtsuensis* sp. nov. and *Ferrimonas kyonanensis* sp. nov., selenate-reducing bacteria belonging to the Gammaproteobacteria isolated from Tokyo Bay. *Int. J. Syst. Evol. Microbiol.* 56, 2639–2645. doi: 10.1099/ijs.0.64399-0

Nevin, K. P., and Lovley, D. R. (2002). Mechanisms for accessing insoluble Fe(III) oxide during dissimilatory Fe(III) reduction by *Geothrix fermentans*. *Appl. Environ. Microbiol.* 68, 2294–2299. doi: 10.1128/AEM.68.5.2294-2299.2002

Newsome, L., Solano Arguedas, A., Coker, V. S., Boothman, C., and Lloyd, J. R. (2020). Manganese and cobalt redox cycling in laterites; Biogeochemical and bioprocessing implications. *Chem. Geol.* 531:119330. doi: 10.1016/j.chemgeo.2019.119330

Niggemyer, A., Spring, S., Stackebrandt, E., and Rosenzweig, R. F. (2001). Isolation and characterization of a novel As(V)-reducing bacterium: Implications for arsenic mobilization and the genus *Desulfitobacterium*. *Appl. Environ. Microbiol.* 67, 5568–5580. doi: 10.1128/AEM.67.12.5568-5580.2001

Nolan, M., Sikorski, J., Davenport, K., Lucas, S., Del Rio, T. G., Tice, H., et al. (2010). Complete genome sequence of *Ferrimonas balearica* type strain (PAT). *Stand. Genomic Sci.* 3, 174–182. doi: 10.4056/sigs.1161239

Northup, D. E., Barns, S. M., Yu, L. E., Spilde, M. N., Schelble, R. T., Dano, K. E., et al. (2003). Diverse microbial communities inhabiting ferromanganese deposits in Lechuguilla and Spider Caves. *Environ. Microbiol.* 5, 1071–1086. doi: 10.1046/j.1462-2920.2003.00500.x

Novotnik, B., Zorz, J., Bryant, S., and Strous, M. (2019). The effect of dissimilatory manganese reduction on lactate fermentation and microbial community assembly. *Front. Microbiol.* 10:1007. doi: 10.3389/fmicb.2019.01007

Ogg, C. D., Greene, A. C., and Patel, B. K. C. (2010). *Thermovenabulum gondwanense* sp. nov., a thermophilic anaerobic Fe(III)-reducing bacterium isolated from microbial mats thriving in a Great Artesian Basin bore runoff channel. *Int. J. Syst. Evol. Microbiol.* 60, 1079–1084. doi: 10.1099/ijs.0.009886-0

Ogg, C. D., and Patel, B. K. C. (2010). *Fervidicella metallireducens* gen. nov., sp. nov., a thermophilic, anaerobic bacterium from geothermal waters. *Int. J. Syst. Evol. Microbiol.* 60, 1394–1400. doi: 10.1099/ijs.0.014670-0

Palmer, F. E., Staley, J. T., Murray, R. G. E., Counsell, T., and Adams, J. B. (1986). Identification of manganese‐oxidizing bacteria from desert varnish. *Geomicrobiol. J.* 4, 343–360. doi: 10.1080/01490458609385943

Palomo, A., Fowler, S. J., Gülay, A., Rasmussen, S., Sicheritz-Pontén, T., and Smets, B. F. (2016). Metagenomic analysis of rapid gravity sand filter microbial communities suggests novel physiology of *Nitrospira* spp.. *ISME J.* 10, 2569–2581. doi: 10.1038/ismej.2016.63

Park, J. H., Kim, B.-S., and Chon, C.-M. (2018). Characterization of iron and manganese minerals and their associated microbiota in different mine sites to reveal the potential interactions of microbiota with mineral formation. *Chemosphere* 191, 245–252. doi: 10.1016/j.chemosphere.2017.10.050

Park, S.-J., Kang, C.-H., Nam, Y.-D., Bae, J.-W., Park, Y.-H., Quan, Z.-X., et al. (2006). *Oceanisphaera donghaensis* sp. nov., a halophilic bacterium from the East Sea, Korea. *Int. J. Syst. Evol. Microbiol.* 56, 895–898. doi: 10.1099/ijs.0.64116-0

Peng, Q., Shaaban, M., Wu, Y., Hu, R., Wang, B., and Wang, J. (2016). The diversity of iron reducing bacteria communities in subtropical paddy soils of China. *Appl. Soil Ecol.* 101, 20–27. doi: 10.1016/j.apsoil.2016.01.012

Piazza, A., Ciancio Casalini, L., Pacini, V. A., Sanguinetti, G., Ottado, J., and Gottig, N. (2019). Environmental bacteria involved in manganese(II) oxidation and removal from groundwater. *Front. Microbiol.* 10:119. doi: 10.3389/fmicb.2019.00119

Pierra, M., Carmona-Martínez, A. A., Trably, E., Godon, J.-J., and Bernet, N. (2015). Specific and efficient electrochemical selection of *Geoalkalibacter subterraneus* and *Desulfuromonas acetoxidans* in high current-producing biofilms. *Bioelectrochemistry* 106, 221–225. doi: 10.1016/j.bioelechem.2015.02.003

Pringsheim, E. G. (1949). Iron bacteria. *Biol. Rev. Camb. Philos. Soc.* 24, 200–245. doi: 10.1111/j.1469-185X.1949.tb00575.x

Prozorov, T., Perez-Gonzalez, T., Valverde-Tercedor, C., Jimenez-Lopez, C., Yebra-Rodriguez, A., Körnig, A., et al. (2014). Manganese incorporation into the magnetosome magnetite: magnetic signature of doping. *Eur. J. Mineral.* 26, 457–471. doi: 10.1127/0935-1221/2014/0026-2388

Rajasabapathy, R., Mohandass, C., Dastager, S. G., Liu, Q., Li, W.-J., and Colaço, A. (2015). *Citreicella manganoxidans* sp. nov., a novel manganese oxidizing bacterium isolated from a shallow water hydrothermal vent in Espalamaca (Azores). *Antonie Van Leeuwenhoek* 108, 1433–1439. doi: 10.1007/s10482-015-0597-x

Ren, B., Li, C., Zhang, X., and Zhang, Z. (2019). Fe(II)-dosed ceramic membrane bioreactor for wastewater treatment: Nutrient removal, microbial community and membrane fouling analysis. *Sci. Total Environ.* 664, 116–126. doi: 10.1016/j.scitotenv.2019.02.019

Ren, F., Zhang, L., Song, L., Xu, S., Xi, L., Huang, L., et al. (2014). *Fulvimarina manganoxydans* sp. nov., isolated from a deep-sea hydrothermal plume in the south-west Indian Ocean. *Int. J. Syst. Evol. Microbiol.* 64, 2920–2925. doi: 10.1099/ijs.0.060558-0

Richardson, L. L., Aguilar, C., and Nealson, K. H. (1988). Manganese oxidation in pH and O_2_ microenvironments produced by phytoplankton. *Limnol. Oceanogr.* 33, 352–363. doi: 10.4319/lo.1988.33.3.0352

Rivadeneyra, A., Gonzalez-Martinez, A., Portela, G. R., Martin-Ramos, D. J., Gonzalez-Lopez, J., and Rivadeneyra, M. A. (2017). Biomineralisation of carbonate and sulphate by the halophilic bacterium *Halomonas maura* at different manganese concentrations. *Extremophiles* 21, 1049–1056. doi: 10.1007/s00792-017-0965-8

Rosenberg, E., DeLong, E. F., Lory, S., Stackebrandt, E., and Thompson, F. L. (2014a). The Prokaryotes: Alphaproteobacteria and Betaproteobacteria. Berlin: Springer.

Rosenberg, E., DeLong, E. F., Lory, S., Stackebrandt, E., and Thompson, F. L. (2014b). The Prokaryotes: Deltaproteobacteria and Epsilonproteobacteria. Berlin: Springer.

Rosenberg, E., DeLong, E. F., Lory, S., Stackebrandt, E., and Thompson, F. L. (2014c). The Prokaryotes: Other Major Lineages of Bacteria and The Archaea. Berlin: Springer.

Sani, R. K., Peyton, B. M., Smith, W. A., Apel, W. A., and Petersen, J. N. (2002). Dissimilatory reduction of Cr(VI), Fe(III), and U(VI) by *Cellulomonas* isolates. *Appl. Microbiol. Biotechnol.* 60, 192–199. doi: 10.1007/s00253-002-1069-6

Sass, H., and Cypionka, H. (2004). Isolation of sulfate-reducing bacteria from the terrestrial deep subsurface and description of *Desulfovibrio cavernae* sp. nov.. *Syst. Appl. Microbiol.* 27, 541–548. doi: 10.1078/0723202041748181

Senko, J. M., Zhang, G., McDonough, J. T., Bruns, M. A., and Burgos, W. D. (2009). Metal reduction at low pH by a *Desulfosporosinus* species: Implications for the biological treatment of acidic mine drainage. *Geomicrobiol. J.* 26, 71–82. doi: 10.1080/01490450802660193

Sharak Genthner, B. R., and Devereux, R. (2015). “*Desulfomicrobium*”, in Bergey’s Manual of Systematics of Archaea and Bacteria, eds. P. de Vos, J. Chun, S. Dedysh, B. Hedlund, P. Kämpfer, F. A. Rainey, et al. (Hoboken, NJ: Wiley), 1–9.

Sharma, M., Khurana, H., Singh, D. N., and Negi, R. K. (2021). The genus *Sphingopyxis*: Systematics, ecology, and bioremediation potential - A review. *J. Environ. Manage.* 280:111744. doi: 10.1016/j.jenvman.2020.111744

Shoiful, A., Kambara, H., Cao, L. T. T., Matsushita, S., Kindaichi, T., Aoi, Y., et al. (2020). Mn(II) oxidation and manganese-oxide reduction on the decolorization of an azo dye. *Int. Biodeterior. Biodegradation* 146:104820. doi: 10.1016/j.ibiod.2019.104820

Shrestha, R., Cerna, K., Spanek, R., Bartak, D., Cernousek, T., and Sevcu, A. (2022). The effect of low-pH concrete on microbial community development in bentonite suspensions as a model for microbial activity prediction in future nuclear waste repository. *Sci. Total Environ.* 808:151861. doi: 10.1016/j.scitotenv.2021.151861

Si, M., Zhao, C., Burkinshaw, B., Zhang, B., Wei, D., Wang, Y., et al. (2017). Manganese scavenging and oxidative stress response mediated by type VI secretion system in *Burkholderia thailandensis*. *Proc. Natl. Acad. Sci. U.S.A.* 114, E2233–E2242. doi: 10.1073/pnas.1614902114

Siering, P. L., and Ghiorse, W. C. (1997a). Development and application of 16S rRNA-targeted probes for detection of iron- and manganese-oxidizing sheathed bacteria in environmental samples. *Appl. Environ. Microbiol.* 63, 644–651. doi: 10.1128/aem.63.2.644-651.1997

Siering, P. L., and Ghiorse, W. C. (1997b). PCR detection of a putative manganese oxidation gene (*mofA*) in environmental samples and assessment of *mofA* gene homology among diverse manganese‐oxidizing bacteria. *Geomicrobiol. J.* 14, 109–125. doi: 10.1080/01490459709378038

Singh, R., Grigg, J. C., Qin, W., Kadla, J. F., Murphy, M. E. P., and Eltis, L. D. (2013). Improved manganese-oxidizing activity of DypB, a peroxidase from a lignolytic bacterium. *ACS Chem. Biol.* 8, 700–706. doi: 10.1021/cb300608x

Sjöberg, S., Callac, N., Allard, B., Smittenberg, R. H., and Dupraz, C. (2018). Microbial communities inhabiting a rare earth element enriched birnessite-type manganese deposit in the Ytterby Mine, Sweden. *Geomicrobiol. J.* 35, 657–674. doi: 10.1080/01490451.2018.1444690

Slobodkina, G. B., Kolganova, T. V., Chernyh, N. A., Querellou, J., Bonch-Osmolovskaya, E. A., and Slobodkin, A. I. (2009). *Deferribacter autotrophicus* sp. nov., an iron(III)-reducing bacterium from a deep-sea hydrothermal vent. *Int. J. Syst. Evol. Microbiol.* 59, 1508–1512. doi: 10.1099/ijs.0.006767-0

Sly, L. I., Arunpairojana, V., and Hodgkinson, M. C. (1988). *Pedomicrobium manganicum* from drinking-water distribution systems with manganese-related “dirty water” problems. *Syst. Appl. Microbiol.* 11, 75–84. doi: 10.1016/S0723-2020(88)80051-1

Song, F., Zhang, G., Li, H., Ma, L., and Yang, N. (2022). Comparative transcriptomic analysis of *Stenotrophomonas* sp. MNB17 revealed mechanisms of manganese tolerance at different concentrations and the role of histidine biosynthesis in manganese removal. *Ecotoxicol. Environ. Saf.*, 244:114056. doi: [10.1016/j.ecoenv.2022.114056](https://doi.org/10.1016/j.ecoenv.2022.114056)

Stolz, J. F., Oremland, R. S., Paster', B. J., Dewhirst, F. E., and Vandamme, P. (2015). “*Sulfurospirillum*”, in Bergey’s Manual of Systematics of Archaea and Bacteria, eds. P. de Vos, J. Chun, S. Dedysh, B. Hedlund, P. Kämpfer, F. A. Rainey, et al. (Hoboken, NJ: Wiley), 1–7.

Straub, K. L., Hanzlik, M., and Buchholz-Cleven, B. E. (1998). The use of biologically produced ferrihydrite for the isolation of novel iron-reducing bacteria. *Syst. Appl. Microbiol.* 21, 442–449. doi: 10.1016/S0723-2020(98)80054-4

Su, G., Zopfi, J., Yao, H., Steinle, L., Niemann, H., and Lehmann, M. F. (2020). Manganese/iron‐supported sulfate‐dependent anaerobic oxidation of methane by archaea in lake sediments. *Limnol. Oceanogr.* 65, 863–875. doi: 10.1002/lno.11354

Su, J., Bao, P., Bai, T., Deng, L., Wu, H., Liu, F., et al. (2013). Cota, a multicopper oxidase from *Bacillus pumilus* WH4, exhibits manganese-oxidase activity. *PLoS One* 8:e60573. doi: 10.1371/journal.pone.0060573

Subari, F., Kamaruzzaman, M. A., Sheikh Abdullah, S. R., Hasan, H. A., and Othman, A. R. (2018). Simultaneous removal of ammonium and manganese in slow sand biofilter (SSB) by naturally grown bacteria from lake water and its diverse microbial community. *J. Environ. Chem. Eng.* 6, 6351–6358. doi: 10.1016/j.jece.2018.09.053

Sugio, T., Tsujita, Y., Hirayama, K., Inagaki, K., and Tano, T. (1988). Mechanism of tetravalent manganese reduction with elemental sulfur by *Thiobacillus ferrooxidans*. *Agric. Biol. Chem.* 52, 185–190. doi: 10.1080/00021369.1988.10868644

Sujith, P. P., and Bharathi, P. A. L. (2011). “Manganese oxidation by bacteria: Biogeochemical aspects”, in Molecular Biomineralization: Aquatic Organisms Forming Extraordinary Materials, ed. W. E. G. Müller (Berlin: Springer), 49–76.

Sujith, P. P., Mourya, B. S., Krishnamurthi, S., Meena, R. M., and Loka Bharathi, P. A. (2014). Mobilization of manganese by basalt associated Mn(II)-oxidizing bacteria from the Indian Ridge System. *Chemosphere* 95, 486–495. doi: 10.1016/j.chemosphere.2013.09.103

Sun, Y., Zhang, Y., Li, W., Zhang, W., Xu, Z., Dai, M., et al. (2021). Combination of the endophytic manganese-oxidizing bacterium *Pantoea eucrina* SS01 and biogenic Mn oxides: An efficient and sustainable complex in degradation and detoxification of malachite green. *Chemosphere* 280:130785. doi: 10.1016/j.chemosphere.2021.130785

Sylvan, J. B., Hoffman, C. L., Momper, L. M., Toner, B. M., Amend, J. P., and Edwards, K. J. (2015). *Bacillus rigiliprofundi* sp. nov., an endospore-forming, Mn-oxidizing, moderately halophilic bacterium isolated from deep subseafloor basaltic crust. *Int. J. Syst. Evol. Microbiol.* 65, 1992–1998. doi: 10.1099/ijs.0.000211

Takeda, M., Kamagata, Y., Ghiorse, W. C., Hanada, S., and Koizumi, J.-I. (2002). *Caldimonas manganoxidans* gen. nov., sp. nov., a poly(3-hydroxybutyrate)-degrading, manganese-oxidizing thermophile. *Int. J. Syst. Evol. Microbiol.* 52, 895–900. doi: 10.1099/00207713-52-3-895

Tang, W., Wu, L., Gong, J., Ye, G., and Zeng, X. (2014). Screening, identification, and removal dynamics of a novel iron-manganese removal strain. *Biomed. Mater. Eng.* 24, 2049–2056. doi: 10.3233/BME-141014

Tang, W., Xia, J., Zeng, X., Wu, L., and Ye, G. (2014). Biological characteristics and oxidation mechanism of a new manganese-oxidizing bacteria FM-2. *Biomed. Mater. Eng.* 24, 703–709. doi: 10.3233/BME-130858

Tebo, B. M., Bargar, J. R., Clement, B. G., Dick, G. J., Murray, K. J., Parker, D., et al. (2004). Biogenic manganese oxides: Properties and mechanisms of formation. *Annu. Rev. Earth Planet. Sci.* 32, 287–328. doi: 10.1146/annurev.earth.32.101802.120213

Tebo, B. M., Johnson, H. A., McCarthy, J. K., and Templeton, A. S. (2005). Geomicrobiology of manganese(II) oxidation. *Trends Microbiol.* 13, 421–428. doi: 10.1016/j.tim.2005.07.009

Templeton, A. S., Staudigel, H., and Tebo, B. M. (2005). Diverse Mn(II)-oxidizing bacteria isolated from submarine basalts at Loihi Seamount. *Geomicrobiol. J.* 22, 127–139. doi: 10.1080/01490450590945951

Thatoi, H., Rath, S., and Kheti, N. K. (2023). Optimisation of manganese peroxidase (MnP) activity of *Enterobacter wuhouensis* using response surface method and evaluation of its Maillard reaction products along with lignin degradation ability. *Indian J. Microbiol.* 63, 604–620. doi: 10.1007/s12088-023-01120-6

Therdkiattikul, N., Ratpukdi, T., Kidkhunthod, P., Chanlek, N., and Siripattanakul-Ratpukdi, S. (2020). Manganese-contaminated groundwater treatment by novel bacterial isolates: Kinetic study and mechanism analysis using synchrotron-based techniques. *Sci. Rep.* 10:13391. doi: 10.1038/s41598-020-70355-w

Thevenieau, F., Fardeau, M.-L., Ollivier, B., Joulian, C., and Baena, S. (2007). *Desulfomicrobium thermophilum* sp. nov., a novel thermophilic sulphate-reducing bacterium isolated from a terrestrial hot spring in Colombia. *Extremophiles* 11, 295–303. doi: 10.1007/s00792-006-0039-9

Tikhonova, T. V., Osipov, E. M., Dergousova, N. I., Boyko, K. M., Elizarov, I. M., Gavrilov, S. N., et al. (2023). Extracellular Fe(III) reductase structure reveals a modular organization enabling S-layer insertion and electron transfer to insoluble substrates. *Structure* 31, 174–184.e3. doi: 10.1016/j.str.2022.12.010

Timofeeva, Y. O., Martynenko, E. S., Sidorenko, M. L., Kim, A. V., and Kazarin, V. M. (2024). Taxonomic composition of cultured Fe- and Mn-oxidizing bacteria and microbial abundance in Fe–Mn nodules of different sizes. *Microbiology* 93, 293–304. doi: 10.1134/S0026261723603676

Tong, H., Li, J., Chen, M., Fang, Y., Yi, X., Dong, L., et al. (2023). Iron oxidation coupled with nitrate reduction affects the acetate-assimilating microbial community structure elucidated by stable isotope probing in flooded paddy soil. *Soil Biol. Biochem.* 183:109059. doi: 10.1016/j.soilbio.2023.109059

Valdivia Santibáñez, M. V. (2009). The Role of Microbial Processes in Soil Phosphorus Dynamics [Dissertation]. Faculty of the Graduate School of Cornell University, Cornell.

Van Nguyen, K., Ha, M.-G., Shin, S., Seo, M., Jang, J., Jo, S., et al. (2018). Electrochemical effect on bioleaching of arsenic and manganese from tungsten mine wastes using *Acidithiobacillus* spp.. *J. Environ. Manage.* 223, 852–859. doi: 10.1016/j.jenvman.2018.06.040

Van Waasbergen, L. G., Hildebrand, M., and Tebo, B. M. (1996). Identification and characterization of a gene cluster involved in manganese oxidation by spores of the marine *Bacillus* sp. strain SG-1. *J. Bacteriol.* 178, 3517–3530. doi: 10.1128/jb.178.12.3517-3530.1996

Van Waasbergen, L. G., Hoch, J. A., and Tebo, B. M. (1993). Genetic analysis of the marine manganese-oxidizing *Bacillus* sp. Strain SG-1: Protoplast transformation, Tn917 mutagenesis, and identification of chromosomal loci involved in manganese oxidation. *J. Bacteriol.* 175, 7594–7603. doi: 10.1128/jb.175.23.7594-7603.1993

Vandieken, V., Mußmann, M., Niemann, H., and Jørgensen, B. B. (2006). *Desulfuromonas svalbardensis* sp. nov. and *Desulfuromusa ferrireducens* sp. nov., psychrophilic, Fe(III)-reducing bacteria isolated from Arctic sediments, Svalbard. *Int. J. Syst. Evol. Microbiol.* 56, 1133–1139. doi: 10.1099/ijs.0.63639-0

Vandieken, V., Niemann, H., Engelen, B., and Cypionka, H. (2017). *Marinisporobacter balticus* gen. nov., sp. nov., *Desulfosporosinus nitroreducens* sp. nov. and *Desulfosporosinus fructosivorans* sp. nov., new spore-forming bacteria isolated from subsurface sediments of the Baltic Sea. *Int. J. Syst. Evol. Microbiol.* 67, 1887–1893. doi: 10.1099/ijsem.0.001883

Vandieken, V., Pester, M., Finke, N., Hyun, J.-H., Friedrich, M. W., Loy, A., et al. (2012). Three manganese oxide-rich marine sediments harbor similar communities of acetate-oxidizing manganese-reducing bacteria. *ISME J.* 6, 2078–2090. doi: 10.1038/ismej.2012.41

Venkateswaran, K., Dollhopf, M. E., Aller, R., Stackebrandt, E., and Nealson, K. H. (1998). *Shewanella amazonensis* sp. nov., a novel metal-reducing facultative anaerobe from Amazonian shelf muds. *Int. J. Syst. Bacteriol.* 48, 965–972. doi: 10.1099/00207713-48-3-965

Vigliotta, G., Nutricati, E., Carata, E., Tredici, S. M., de Stefano, M., Pontieri, P., et al. (2007). *Clonothrix fusca* Roze 1896, a filamentous, sheathed, methanotrophic γ-Proteobacterium. *Appl. Environ. Microbiol.* 73, 4272–4280. doi: 10.1128/AEM.02751-06

Villemur, R., Lanthier, M., Beaudet, R., and Lépine, F. (2006). The *Desulfitobacterium* genus. *FEMS Microbiol. Rev.* 30, 706–733. doi: [10.1111/j.1574-6976.2006.00029.x](https://doi.org/10.1111/j.1574-6976.2006.00029.x)

Visser, M., Stams, A. J. M., Frutschi, M., and Bernier-Latmani, R. (2016). Phylogenetic comparison of *Desulfotomaculum* species of subgroup 1a and description of *Desulfotomaculum reducens* sp. nov.. *Int. J. Syst. Evol. Microbiol*. 66, 762–767. doi: [10.1099/ijsem.0.000786](https://doi.org/10.1099/ijsem.0.000786)

Wan, W., Xing, Y., Qin, X., Li, X., Liu, S., Luo, et al. (2020). A manganese-oxidizing bacterial consortium and its biogenic Mn oxides for dye decolorization and heavy metal adsorption. *Chemosphere*, 253:126627. doi: [10.1016/j.chemosphere.2020.126627](https://doi.org/10.1016/j.chemosphere.2020.126627)

Wang, Q., Xiao, M., Fang, K., Du, X., Wang, Z., and Lu, X. (2024). Enhancing manganese removal through pre-deposition of powdered activated carbon-manganese oxide (PAC-MnOx) film on gravity-driven ceramic membrane: The impact of biofilm integrity and fluid dynamics. *Sep. Purif. Technol.* 342:127051. doi: [10.1016/j.seppur.2024.127051](https://doi.org/10.1016/j.seppur.2024.127051)

Wang, Y., Tsang, Y. F., Wang, H., Sun, Y., Song, Y., Pan, X., et al. (2020). Effective stabilization of arsenic in contaminated soils with biogenic manganese oxide (BMO) materials. *Environ. Pollut.* 258:113481. doi: [10.1016/j.envpol.2019.113481](https://doi.org/10.1016/j.envpol.2019.113481)

Wang, Z. [Zhiyong], Wang, J., Liu, J., Chen, H., Li, M., and Li, L. (2017). Mechanistic insights into manganese oxidation of a soil-borne Mn(II)-oxidizing *Escherichia coli* strain by global proteomic and genetic analyses. *Sci. Rep.* 7:1352. doi: 10.1038/s41598-017-01552-3

Wang, Z. [Zhongzhong], Yin, Q., Gu, M., He, K., and Wu, G. (2018). Enhanced azo dye Reactive Red 2 degradation in anaerobic reactors by dosing conductive material of ferroferric oxide. *J. Hazard. Mater.* 357, 226–234. doi: 10.1016/j.jhazmat.2018.06.005

Weelink, S. A. B., van Doesburg, W., Saia, F. T., Rijpstra, W. I. C., Röling, W. F. M., Smidt, H., et al. (2009). A strictly anaerobic betaproteobacterium *Georgfuchsia toluolica* gen. nov., sp. nov. degrades aromatic compounds with Fe(III), Mn(IV) or nitrate as an electron acceptor. *FEMS Microbiol. Ecol.* 70, 575–585. doi: 10.1111/j.1574-6941.2009.00778.x

Wunder, L. C., Breuer, I., Willis-Poratti, G., Aromokeye, D. A., Henkel, S., Richter-Heitmann, T., et al. (2024). Manganese reduction and associated microbial communities in Antarctic surface sediments. *Front. Microbiol.* 15:1398021. doi: 10.3389/fmicb.2024.1398021

Wurzel, E. B. (2000). The reduction of particulate manganese oxides by Carnobacteria in Lake Mendota, Wisconsin. *J. of Undergrad. Res.* 8, 169–174.

Xiao, M., Long, Z., Fu, X., and Zou, L. (2024). ). Versatile applications and mechanisms of genus *Exiguobacterium* in bioremediating heavy metals and organic pollutants: A review. *Int. Biodeterior. Biodegradation* 194:105884. doi: 10.1016/j.ibiod.2024.105884

Xiao, Z., Jiang, Q., Li, Y., Zhou, J., Chen, D., and Xia, T. (2022). Enhanced microbial nitrate reduction using natural manganese oxide ore as an electron donor. *J. Environ. Manage.* 306:114497. doi: 10.1016/j.jenvman.2022.114497

Xiong, J., Xu, Y., LI, Y., and Zeng, X.‑C. (2023). A novel Mn- and Fe-oxides-reducing bacterium with high activity to drive mobilization and release of arsenic from soils. *Water* 15:2337. doi: [10.3390/w15132337](https://doi.org/10.3390/w15132337)

Xu, X., Song, F., Zhang, G., Ma, L., and Yang, N. (2024). Proteomic insights into the response of *Halomonas* sp. Mnb13 to excess Mn(Ⅱ) and the role of H_2_S in Mn(Ⅱ) resistance. *Environ. Res.* 246:118157. doi: 10.1016/j.envres.2024.118157

Yabuuchi, E., Kawamura, Y., and Ezaki, T. (2015). “*Ralstonia*”, in Bergey’s Manual of Systematics of Archaea and Bacteria, eds. P. de Vos, J. Chun, S. Dedysh, B. Hedlund, P. Kämpfer, F. A. Rainey, et al. (Hoboken, NJ: Wiley), 1–12.

Yang, F., Li, J., Wang, H., Xiao, X., Bai, R., and Zhao, F. (2023). Visible light induces bacteria to produce superoxide for manganese oxidation. *Front. Environ. Sci. Eng.* 17:19. doi: 10.1007/s11783-023-1619-y

Yang, W., Zhang, Z., Zhang, Z., Chen, H., Liu, J., Ali, M., et al. (2013). Population structure of manganese-oxidizing bacteria in stratified soils and properties of manganese oxide aggregates under manganese–complex medium enrichment. *PLoS One* 8:e73778. doi: 10.1371/journal.pone.0073778

Yang, Y., Li, Y., and Sun, Q. (2014). Archaeal and bacterial communities in acid mine drainage from metal-rich abandoned tailing ponds, Tongling, China. *Trans. Nonferrous Met. Soc. China* 24, 3332–3342. doi: 10.1016/S1003-6326(14)63474-9

Yin, Y., Li, D., Wang, Y., Xu, Z., Xu, G., Zhao, Z., et al. (2019). Concurrent Removal of Mn(II) and Cr(VI) by *Achromobacter* sp. TY3-4. *Geomicrobiol. J.* 36, 317–325. doi: 10.1080/01490451.2018.1544324

Yu, C., Turner, S., Huotari, S., Chen, N., Shchukarev, A., Österholm, P., et al. (2024). Manganese cycling and transport in boreal estuaries impacted by acidic Mn-rich drainage. *Geochim. Cosmochim. Acta* 365, 136–157. doi: 10.1016/j.gca.2023.12.004

Yu, H., and Leadbetter, J. R. (2020). Bacterial chemolithoautotrophy via manganese oxidation. *Nature*, 583, 453–458. doi: 10.1038/s41586-020-2468-5

Yu, L., He, D., Yang, L., Rensing, C., Zeng, R. J., and Zhou, S. (2022). Anaerobic methane oxidation coupled to ferrihydrite reduction by *Methanosarcina barkeri*. *Sci. Total Environ.* 844:157235. doi: 10.1016/j.scitotenv.2022.157235

Zakaria, Z., Fadzil, F. N. M., Mohamad, M. A. N., Hamid, A. A. A., Chowdhury, A. J. K., and Harumain, Z. A. S. (2024). Metagenomic analysis of bacterial communities in heavy metal leachate-contaminated soils at Jalan Lipis Sanitary Landfill, Pahang, Malaysia. *Desal. Water Treat.* 319:100512. doi: 10.1016/j.dwt.2024.100512

Zakharova, Y. R., Parfenova, V. V., Granina, L. Z., Kravchenko, O. S., and Zemskaya, T. I. (2010). Distribution of iron- and manganese-oxidizing bacteria in the bottom sediments of Lake Baikal. *Inland Water Biol.* 3, 313–321. doi: 10.1134/s1995082910040036

Zapffe, C. (1933). The history of manganese in water supplies and methods for its removal. *J. Am. Water Works Assoc.* 25, 655–676. doi: 10.1002/j.1551-8833.1933.tb18262.x

Zavarzina, D. G., Sokolova, T. G., Tourova, T. P., Chernyh, N. A., Kostrikina, N. A., and Bonch-Osmolovskaya, E. A. (2007). *Thermincola ferriacetica* sp. nov., a new anaerobic, thermophilic, facultatively chemolithoautotrophic bacterium capable of dissimilatory Fe(III) reduction. *Extremophiles* 11, 1–7. doi: 10.1007/s00792-006-0004-7

Zavarzina, D. G., Tourova, T. P., Kuznetsov, B. B., Bonch-Osmolovskaya, E. A., and Slobodkin, A. I. (2002). *Thermovenabulum ferriorganovorum* gen. nov., sp. nov., a novel thermophilic, anaerobic, endospore-forming bacterium. *Int. J. Syst. Evol. Microbiol.* 52, 1737–1743. doi: 10.1099/00207713-52-5-1737

Zeng, X., Zhang, M., Liu, Y., and Tang, W. (2018). Manganese(II) oxidation by the multi-copper oxidase CopA from *Brevibacillus* *panacihumi* MK-8. *Enzyme Microb. Technol.* 117, 79–83. doi: 10.1016/j.enzmictec.2018.04.011

Zhang, H., Liu, F., Zheng, S., Chen, L., Zhang, X., and Gong, J. (2020). The differentiation of iron-reducing bacterial community and iron-reduction activity between riverine and marine sediments in the Yellow River estuary. *Mar. Life Sci. Technol.* 2, 87–96. doi: 10.1007/s42995-019-00001-6

Zhang, L.-M., Liu, F., Tan, W.-F., Feng, X.-H., Zhu, Y.-G., and He, J. (2008). Microbial DNA extraction and analyses of soil iron–manganese nodules. *Soil Biol. Biochem.* 40, 1364–1369. doi: 10.1016/j.soilbio.2007.01.004

Zhang, Y., Tang, Y., Qin, Z., Luo, P., Ma, Z., Tan, M., et al. (2019). A novel manganese oxidizing bacterium-*Aeromonas hydrophila* strain DS02: Mn(II) oxidization and biogenic Mn oxides generation. *J. Hazard. Mater.* 367, 539–545. doi: 10.1016/j.jhazmat.2019.01.012

Zhao, X., Liu, B., Wang, X., Chen, C., Ren, N., and Xing, D. (2020). Single molecule sequencing reveals response of manganese-oxidizing microbiome to different biofilter media in drinking water systems. *Water Res.* 171:115424. doi: 10.1016/j.watres.2019.115424

Zhao, X., Wang, X., Liu, B., Xie, G., and Xing, D. (2018). Characterization of manganese oxidation by *Brevibacillus* at different ecological conditions. *Chemosphere* 205, 553–558. doi: 10.1016/j.chemosphere.2018.04.130

Zhou, H., Pan, H., Xu, J., Xu, W., and Liu, L. (2016). Acclimation of a marine microbial consortium for efficient Mn(II) oxidation and manganese containing particle production. *J. Hazard. Mater.* 304, 434–440. doi: 10.1016/j.jhazmat.2015.11.019

Zhou, L., Zeng, Y., Xu, C., Al-Dhabi, N. A., Wang, S., Sun, S., et al. (2024). Exogenous paths regulate electron transfer enhancing sediment phosphorus immobilization. *Sci. Total Environ.* 951:175689. doi: 10.1016/j.scitotenv.2024.175689

Zhu, J., Yan, X., Zhou, L., Li, N., Liao, C., and Wang, X. (2022). Insight of bacteria and archaea in Feammox community enriched from different soils. *Environ. Res.* 203:111802. doi: 10.1016/j.envres.2021.111802
